# BET & ELF Quantum Topological Analysis of Neutral 2-Aza-Cope Rearrangement of γ-Alkenyl Nitrones

**DOI:** 10.3390/molecules22081371

**Published:** 2017-08-19

**Authors:** Pedro Merino, Maria A. Chiacchio, Laura Legnani, Tomás Tejero

**Affiliations:** 1 Instituto de Biocomputación y Física de Sistemas Complejos (BIFI), Universidad de Zaragoza, Zaragoza, 50009 Aragón, Spain; 2 Dipartimento di Scienze del Farmaco, Università di Catania, 95124 Catania, Italy; ma.chiacchio@unict.it; 3 Dipartimento di Chimica, Università di Pavia, Via Taramelli 12, 27100 Pavia, Italy; laura.legnani@unipv.it; 4 Instituto de Síntesis Química y Catalisis Homogénea (ISQCH), Universidad de Zaragoza-CSIC, Zaragoza, 50009 Aragón, Spain; ttejero@unizar.es

**Keywords:** nitrones, 2-aza-Cope rearrangement, MEDT, ELF analysis, BET analysis

## Abstract

The 2-Aza-Cope rearrangement of γ-alkenyl nitrones is a rare example of the neutral thermal 2-aza-Cope process that usually takes place with cationic species. During the rearrangement, a redistribution of bonds and electronic density occurs in one kinetic step. However, the introduction of substituents with different steric requirements and electronic features might alter the activation energies and the synchronicity of the reaction. The electron localization function (ELF) analysis and its application to Bonding Evolution Theory (BET) analysis within the context of Molecular Electron Density Theory (MEDT) is an excellent tool to monitor the electron density along the reaction coordinate and thus investigate in detail bond breaking and formation and the corresponding energy barriers. By analyzing topological ELF calculations of seventeen 2-aza-Cope nitrone rearrangements with selected substituents, the main factors influencing the synchronicity of the process were investigated. This MEDT study results revealed that the rearrangement is a non-polar process mostly influenced by steric factors rather than by electronic ones, and confirms the pseudoradical character of the process rather than any pericyclic electron-reorganization.

## 1. Introduction

The topological analysis of the gradient field of the electron localization function (ELF) [1] developed by Silvi and Savin [2] added a novel perspective to chemical bonding and constitutes a powerful tool for validating the Molecular Electron Density Theory (MEDT) developed by Domingo [3,4]. MEDT theory introduces the concept of global electron density transfer (GEDT) and ELF analysis allows following the evolution of electron density, thus correlating the GEDT with the course of the reaction. Since MEDT is based on the concept that the capability for changes in electron density is responsible for molecular reactivity, the study of such changes along the reaction coordinate (ELF analysis) is a very valuable tool for validating MEDT. ELF analysis has been widely used for studying electron localization/delocalization [5] and for describing the molecular mechanism of organic reactions [6], particularly in terms of electron density transfers [7]. Some recent examples include Ene-like [8], Mannich-type [9,10], Diels–Alder [11], and (3 + 2) cycloaddition reactions [12,13].

The combination of ELF analysis with Thom’s catastrophe theory (CT) [14] results in Bonding Evolution Theory (BET) [15], a powerful tool for understanding chemical reactivity [16] that represents an enhanced applicability of the topological analysis of ELF [17]. There are a number of studies using BET that demonstrate its utility [18,19,20,21,22,23,24].

By monitoring the electron density along the reaction, ELF analysis allows estimation of the bonding changes along a chemical process [9,10]. In this context, we have recently reported the use of ELF for evaluating the synchronicity of organic reactions using the dual reactivity between oximes and alkenes as a case study [8].

**Scheme 1:**
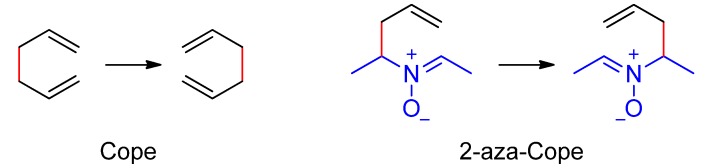
Degenerated Cope rearrangements.

Rearrangements are defined by the IUPAC as “degenerated” when the principal product is indistinguishable (in the absence of isotopic labelling) from the principal reactant [25]. The Cope rearrangement is a typical example [26,27]. By using the BET analysis, Polo and Andrés characterized the mechanism of the reaction and studied the changes induced by the presence of two, three, and four cyano groups in the parent structure [28]. Inclusion of a heteroatom (i.e., hetero-Cope [29] or Claisen [30,31] rearrangements) breaks the symmetry and the rearrangement loss degeneration. The resulting asynchronicity of the process can be evidenced by an ELF analysis [32,33,34]. An exception to the loss of degeneration upon inclusion of a heteroatom is the neutral 2-aza-Cope rearrangement of nitrones (Scheme 1), suggested by Hoffmann in 1986 [35], and confirmed by us more than twenty years later [36,37].

In effect, in 2-aza-Cope rearrangement, product and reactant are also indistinguishable and the formation of the bonds is symmetrical, as for the Cope rearrangement. However, although the two fragments involved in the process, i.e., the allylic moiety and the nitrone function, are isoelectronic (four electrons for three atoms), the presence of nitrogen and oxygen atoms in the latter should induce different electronic properties that might be affected by the presence of substituents. The transition structure (TS) analysis of the 2-aza-Cope reaction was reported in our previous paper with special focus on cyclic nitrones to explain the experimentally observed results on the competitive intramolecular dipolar cycloaddition [37]. Herein, we applied the ELF analysis to study in detail the neutral 2-aza-Cope rearrangement of nitrones by comparing up to 17 differently substituted substrates. A detailed analysis of the course of the reaction in each case will be performed. The influence of the substitution pattern and the electronic features of the substituents on the synchronicity of the reaction will also be discussed.

## 2. Computational Methods

All of the calculations were performed using the Gaussian09 program [38]. Molecular geometries were optimized with the B3LYP functional [39,40] in conjunction with 6-31G(d) basis set [41,42,43]. Analytical second derivatives of the energy were calculated to classify the nature of every stationary point, to determine the harmonic vibrational frequencies, and to provide zero-point vibrational energy corrections. The thermal and entropic contributions to the free energies were also obtained from the vibrational frequency calculations, using the unscaled frequencies. All TSs were characterized by one imaginary frequency and were confirmed to connect to reactants and products by intrinsic reaction coordinate (IRC) calculations [44,45]. The IRC paths were traced using the second order González–Schlegel integration method [46,47]. The electronic structures of stationary points were analyzed by the topological analysis of the gradient field of electron localization function (ELF) [48,49]. The ELF study was performed with the TopMod program [50] using the corresponding wavefunctions of the all structures of the IRC. Structural representations were generated using CYLView [51].

## 3. Results

**Scheme 2:**
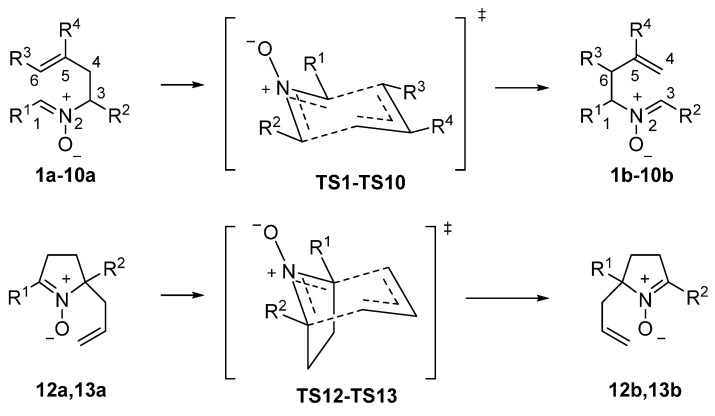
2-Aza-Cope rearrangement of γ-Alkenyl Nitrones (see Table 1).

We chose for the study nitrones **1a**–**13a** (Scheme 2). Nitrones **1a**–**3a** were selected for evaluating the electronic influence of different aromatic substituents at the nitrone carbon(s). Similarly, nitrones **4a**–**6a** will allow estimating the effects for alkyl substituents with different electronic features. Nitrones **7a**–**9a** include substituents at the terminal position of the allyl moiety, comprising a bulky, electron-donating and electron-attractor substituents to study steric and electronic effects during the rearrangement. During the rearrangement, the allylic fragment acts as the nucleophilic part of the molecule and the nitrone moiety as the electrophilic one. This is in agreement with the fact that the process is catalyzed by Brønsted acids. Protonation of the nitrone oxygen renders the nitrone moiety more electrophilic, moving the rearrangement from neutral to cationic 2-aza-Cope and increasing the reaction rate [37]. Consequently, a transfer of electron density from the allylic fragment towards the nitrone function is expected. Nitrone **10** is symmetrical but an electron-donating group has been placed at the allylic fragment in an attempt of stimulating the transfer of charge from the allylic fragment towards the nitrone function.

With the aim of increasing the polarity of the process, we selected nitrone **11**, bearing an electron-withdrawing group at the nitrone moiety and an electron-donating group at the allylic fragment. Nitrones **7**–**9** and **11** present two possible isomers at the allylic double bond that will be considered separately. In all cases, we have only considered the most stable (*Z*)-isomer of the nitrone functional group. Finally, we have also studied two representative cyclic substrates, i.e., nitrones **12** and **13**, the latter having a quaternary center.

The 2-aza-Cope rearrangement of nitrones takes place as a classical Cope rearrangement, that is in one step through a single TS with concomitant breaking and formation of the corresponding bonds and migration of the nitrone function and allylic double bond. All the TSs **TS01**–**TS13** were located and characterized. The corresponding absolute and relative energy barriers are collected in Table 1.

To evaluate the intramolecular transfer of electron density, we divided the alkenyl nitrone in two fragments, i.e., the allylic fragment, comprising the three allylic carbon atoms (C4, C5, and C6) and their substituents, and the nitrone fragment composed by C1, N2, and C3 atoms and their substituents. The global electron density transfer (GEDT) was calculated as the difference between the allylic fragment and the nitrone fragment [52]. In most cases, the obtained positive values confirmed the transfer of electron density from the allylic fragment towards the nitrone one. Only in the case of nitrone **9** a negative value, but close to zero, was obtained. In any case, all GEDT values were found to be lower than 0.2 e, confirming the low polar character of the rearrangement. The highest value was 0.17 e, corresponding to nitrone **11**, the most favorable option for an internal density transfer from the allylic fragment to the nitrone one. Nitrones **12** and **13** also present very low values point to apolar processes.

**Figure 1:**
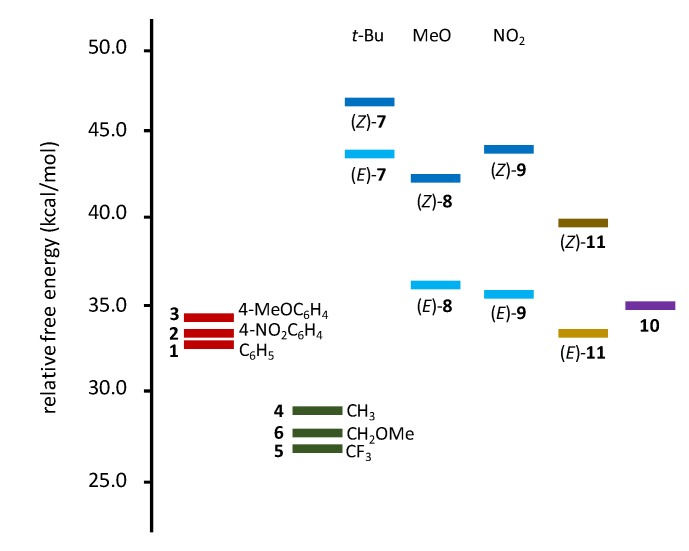
Energy barriers for the 2-aza-Cope rearrangement of nitrones **1**–**11**. For nitrones **1**–**6**, aromatic (red series) and aliphatic (green series) substituents correspond to one side of the nitrone fragment. For nitrones **7**–**9** (R^1^ = R^2^ = Ph), (*E*)-isomers (cyan) have lower barriers than (*Z*)-isomers (blue); substituents correspond to allylic fragment.

However, although a trend can be observed that substituents rendering the nitrone fragment more electrophilic (e.g., trifluoromethyl) lead to lower energy barriers, there is no clear correlation between the energy barriers and GEDT, as is found in the case of polar reactions. [4] Indeed, no clear correlation between electronic effects and energy barriers are found. For instance, nitrones **4** and **5**, in which the only difference is the replacement of the methyl group in **4** by a trifluoromethyl group in **5**, have similar energy barriers, as well as nitrone **6** with a methoxymethyl group instead the methyl group. In the case of nitrones **7**–**9**, little differences were observed (e.g., 36.9 and 36.0 kcal/mol for (*E*)-**8** and (*E*)-**9**, respectively) despite their substituents (methoxy and nitro groups) having rather different electronic features. On the other hand, for the same nitrones, important differences were found between E/Z isomers at the allylic fragment (e.g., 36.9 and 42.4 kcal/mol for (*E*)-**8** and (*Z*)-**8**, respectively, and 36.0 and 43.0 kcal/mol for (*E*)-**9** and (*Z*)-**9**, respectively). These data suggest that the rearrangement is much more sensitive to steric effects than to the electron-withdrawing/donating capacity of substituents (electronic effects). In fact, whereas no difference (just 0.6 kcal/mol) is found between sterically identical but electronically different nitrones **2** and **3**, a difference of 4.1 kcal/mol is observed between the energy barriers of nitrones **1** and **4**, in which the phenyl group has been replaced by a methyl group. Indeed, nitrone **4** is one of those presenting a low barrier. The lowest barrier is observed for nitrone **5,** which present similar steric requirements and, in addition, increase the electrophilic character of the nitrone fragment. Nitrone **6** also has a low barrier due to the similarity with nitrone **4**. On the other hand, the highest energy barriers correspond to both isomers of nitrone **7** which is the most sterically hindered. Figure 1 summarizes the above observations, evidencing the lower barriers of aliphatic substituents (green series) with respect of aromatic ones (red series) in the case of unsubstituted allylic fragments. For nitrones **7**–**9** with two phenyl rings at the nitrone fragment and different substituents at the allylic fragment, (*Z*)-isomers (cyan series) are more stable than (*E*)-isomers (blue series). Nitrones **12** and **13** (not shown in Figure 1) have intermediate values of 34.4 and 30.9 kcal/mol, respectively.

The intrinsic features of the rearrangement point to a process in which one bond is breaking while the other is forming, but the concertedness of the process should be analyzed in depth because bond breaking and forming might not be simultaneous. The introduction of substituents might alter the synchronicity of the process. A first analysis of the distances C1–C6 and C3–C4 of the TSs reflected that both bonds only are strictly formed at the same time in the case of symmetrical nitrones by obvious reasons. For the rest of the nitrones, the bond forming/breaking distances are different depending on the substituents (Table 1). As in the case of energy barriers, a clear correlation between the differences of bond forming/breaking distances and the electronic nature of the substituents was not found, reinforcing the idea that steric factors govern the rearrangement. Several observations further support this hypothesis, i.e., (i) very similar distances are observed for nitrones **1**–**3**, independently of the electronic features of substituents, (ii) the longest distances correspond to the most sterically hindered nitrones (*Z*)-**7**, whereas the shortest distances are for the less hindered nitrones **10**, and (iii) the differences observed in distances at TSs for unsymmetrical nitrones do not depend on the electronics of the adjacent substituent, but rather on its steric requirements. A clear example is given by nitrone **13** with two substituents electronically very different but with similar steric requirements. According to a steric dependence, distances at the TS are the same (1.75 Å).

**Figure 2:**
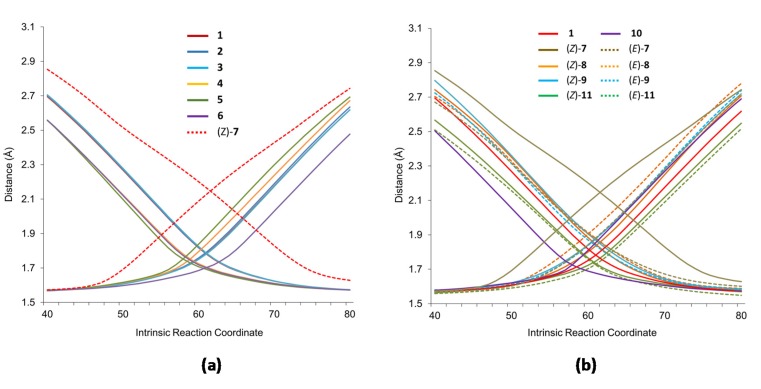
Evolution of breaking/forming bonds along the reaction coordinate (20 points before and after the TSs, **P61** are given) of the 2-aza-Cope rearrangement of γ-alkenyl nitrones **1**–**6** (and (*Z*)-**7**) (**a**), and **7**–**11** and **1** (**b**). Ascending and descending trajectories correspond to breaking (C3–C4) and forming (C1–C6) bonds, respectively.

Figure 2 shows a comparison of the evolution of distances along the reaction coordinate for nitrones **1**–**6** (left) and nitrones **7**–**11** (right). The evolution of distances for (*Z*)-**7**, the most hindered case, and **1** in left and right diagrams, respectively, have also been included as a reference. In all cases, IRCs have 121 points and the TS corresponds to point 61. Figure 2 confirms the strong dependence on the rearrangement of steric factors rather than electronic ones.

The ELF analysis can be used to analyze more in detail the synchronicity [8] of this rearrangement and to detect subtle differences that are not evident with the only consideration of atom distances in the transition structure. With this aim, we performed ELF analyses of all the IRC analyses of the rearrangements listed in Table 1 (for details on basin populations and numerical data see supplementary information).

**Figure 3:**
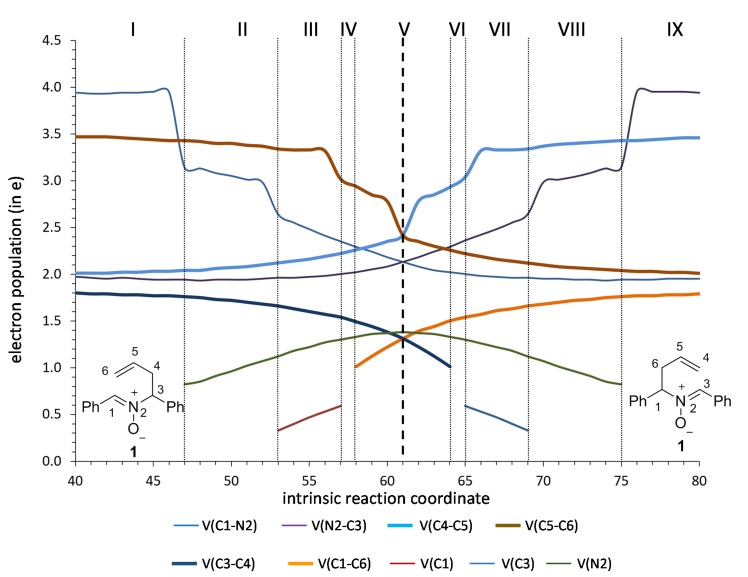
Graphical representation of the basin-population changes along the 2-aza-Cope rearrangement of nitrone **1**. Only atoms/bonds that change their electron density and a zoom of 40 points before and after the transition structure is shown in all Figures. For the complete representations, see supplementary information.

**Figure 4:**
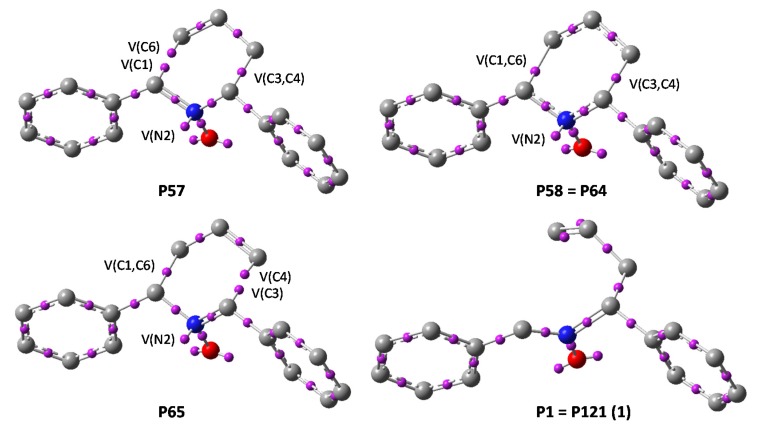
ELF attractor positions for the most relevant points of the IRC associated with the 2-aza-Cope rearrangement of nitrone **1**. Representation of TS (**P61**) is essentially the same that **P58=P64**, the differences being in distances and basins populations but no substantial differences are appreciated in descriptors positions.

As expected, the ELF analysis for nitrone **1** was completely symmetrical, corresponding to a one-step process (Figure 3). We carried out a BET analysis for the degenerated rearrangement of nitrone **1**, identifying nine differentiated ELF structural stability domains (SSD). SSDs VI, VII, VIII, and IX are complementary to SSDs I, II, III, and IV, respectively, by virtue of the symmetry of the process. The population of the most significant valence basins are listed in Table 2. A schematic picture of the attractor positions of the ELF for the relevant points along the IRC is given in Figure 4, as well as a graphic representation of the basin-population changes along the reaction path.

SSD I, 3.654 Å ≥ d(C1–C6) > 2.405 Å and 1.552 Å ≤ d(C3–C4) < 1.590 Å begins at nitrone **1** being a minimum connecting itself with **TS1**. ELF analysis shows monosynaptic basins V1(O3) and V2(O3), and disynaptic basins V(C1,N2) and V(N2,O3) associated with nitrone oxygen lone pairs, the C=N double bond, and the N–O bond of the nitrone framework. Two disynaptic basins V1(C5,C6) and V2(C5,C6), as well as one disynaptic basin V(C4,C5) associated with C5=C6 double bond and C4–C5 bond of the allylic system were also observed. Disynaptic basin V(C3–C4) corresponded to the C3–C4 single bond. Populations of those basins (see Table 2 and supplementary information for full data) correspond to the expected bond order. At the end of this SSD, some change in electronic density is observed since disynaptic basin V(C1,N2) starts depopulation, going from 3.95 e to 3.14 e. This change indicates that electron density starts to migrate from C1=N2 double bond.

SSD II 2.405 Å ≥ d(C1–C6) > 2.128 Å and 1.590 Å ≤ d(C3–C4) < 1.634 Å starts at point 47 of the IRC when a monosynaptic basin V(N2) appears. Depopulation of basin V(C1,N2) goes from 3.14 e to 2.35 e. This basin corresponds to the C1=N2 double bond that is going to be transformed into a single bond. This topological change corroborates the displacement of electron density in the nitrone region towards the nitrone nitrogen. In fact, the electron density of the V(N2) basin proceeds from the depopulation of the V(C1,N2). The monosynaptic basin V(N2) suggests the formation of a pseudoradical center at the heteroatom. At the end of this phase, the C1–N2 bond is essentially a single bond. Disynaptic basins V(C3,N2) and V(C4,C5) start to increase their populations slightly and most of electron density is received by the pseudoradical center at N2.

SSD III, 2.128 Å ≥ d(C1–C6) > 1.944 Å and 1.634 Å ≤ d(C3–C4) < 1.685 Å, starts at point 54 of the IRC when new V(C1) monosynaptic basin is created. Electron density of this basin is mainly reached through the depopulation of V(C1,N2) disynaptic basin which continues its depopulation. The disynaptic basin V(C1,C6) also suffers a progressive depopulation that continues during this SSD. The observed topological changes represent a continuation of the electron density migration towards the TS, the most important observation being the appearance of a pseudoradical center at C1 with a population of 0.33 e that is increased up to 0.59 e at the end of the SSD.

SSD IV, 1.944 Å ≥ d(C1–C6) > 1.899 Å and 1.685 Å ≤ d(C3–C4) < 1.702 Å, begins at point 57 of the IRC when new V(C6) monosynaptic basin is created prior to the formation of the C1–C6 single bond. Monosynaptic basins V(C1) and V(C6) reach populations of 0.59 e and 0.20 e, respectively, revealing the formation of C1 and C6 pseudoradical centers, which is the most important topological change of this SSD.

SSD V, 1.899 Å ≥ d(C1–C6) > 1.702 Å and 1.702 Å ≤ d(C3–C4) < 1.890 Å, begins at point 58 of the IRC when monosynaptic basin V(C1) and V(C6) are merged into a new V(C1,C6) disynaptic basin, indicating that formation of the new C1–C6 single bond has already begun at a length of 1.899 Å. This SSD contains the TS of the reaction and the observed topological changes reveal the formation of a new C1–C6 bond and breaking of the C3–C4 bond. Also, while populations of V(C1,N2) and V(C3,N2) disynaptic basins (2.13 e in both cases) are close to a single bond character at TS, just in the middle of this SSD, the population at monosynaptic V(N2) basin reach a maximum with a value of 1.38 e. This topological change indicates migration of electron density from the C1–N2 bond to the C3–N2 bond, but through an intermediate pseudoradical at N2.

SSD VI, 1.702 Å ≥ d(C1–C6) > 1.685 Å and 1.890 Å ≤ d(C3–C4) < 1.944 Å, begins at point 64 of the IRC when new V(C3) and V(C4) monosynaptic basins are created, indicating breaking of the C3–C4 single bond. With populations of 0.33 e and 0.20 e for V(C3) and V(C4), this topological change indicates the formation of pseudoradical centers at C3 and C4. At the same time, disynaptic basins V(N2,C3) and V(C4,C5) start to increase their population. This SSD is equivalent to SSD IV.

SSD VII, 1.685 Å ≥ d(C1–C6) > 1.633 Å and 1.944 Å ≤ d(C3–C4) < 2.129 Å, begins at point 65 of the IRC with the disappearance of monosynaptic basin V(C4), while V(C3) is becoming depopulated. Disynaptic basin V(C4,C5) is close to its maximum value (3.34 e), compatible with the formation of C4=C5 double bond, which is essentially formed at the end of this SSD. These topological changes indicate the continuation of electron density migration, this SSD being equivalent to SSD III.

SSD VIII, 1.633 Å ≥ d(C1–C6) > 1.590 Å and 2.129 Å ≤ d(C3–C4) < 2.405 Å, begins at point 69 of the IRC when monosynaptic basin V(C3) disappears and V(N2,C3) continues increasing the population in this SSD equivalent to II.

SSD IX, 1.590 Å ≥ d(C1–C6) > 1.552 Å and 2.405 Å ≤ d(C3–C4) < 3.654 Å, begins at point 75 of the IRC when monosynaptic basin V(N2) disappears and disynaptic basin V(N2,C3) increases its population to the maximum, at which time V(C1,N2) and V(C5,C6) reach their minimum population with C1–N2 single bonds. This topological change indicates the complete formation of the N2=C3 double bond as well as C1–N2 and C5–C6 single bonds, leading to the starting nitrone **1**.

**Scheme 3:**
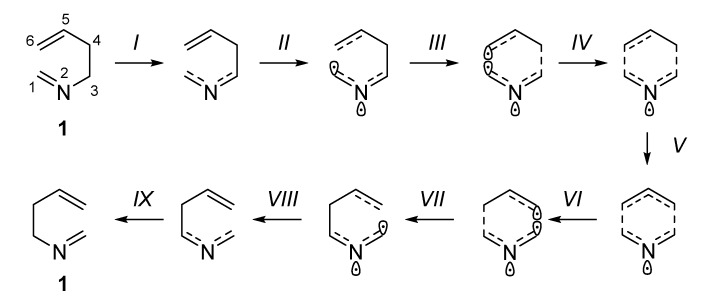
Lewis structures for the 2-Aza-Cope rearrangement of γ-Alkenyl Nitrones (see Table 2).

In summary, the BET analysis characterizes the 2-aza-Cope rearrangement of nitrones by the Lewis structures illustrated in Scheme 3.

According to the analysis, the rearrangement is topologically defined by nine differentiated SSDs starting with the depopulation of the C1=N2 double bond along SSDs I to V and the C5=C6 double bond along SSDs II to V. As a consequence of those depopulations, pseudoradical centers appear at C1 and C6 during SSD IV that become a new C1–C6 bond at SSD V. The opposite takes paces during SSDs VI (formation of pseudoradical centers at C1 and C6) and VII (breaking of the C1–C6 bond). Migration of the double bond from C1=N2 to C3=N2 involves formation of a pseudoradical center at N2 during SSDs II to VIII. A comparison with the BET analysis of the parent Cope rearrangement reported by Polo and Andrés showed several similarities between the two mechanisms. Both reactions are characterized by nine SSDs, and in both cases processes takes place in the vicinity of the transition structure. However, in the case of the 2-aza-Cope rearrangement, the presence of the nitrogen atom breaks the symmetry and introduces differences with respect to the classical Cope rearrangement. Among these differences are the presence of a pseudoradical center at the nitrogen atom, which is formed during the initial stages of the reaction, and a higher similarity in some points with the Cope rearrangement of cyano derivatives, rather than with the unsubstituted substrate.

Silvi and co-workers recently reported that the electron density transfers observed along the reaction pathway are always correlated with the deformations of the molecular geometry [7]. The case of 2-aza-Cope rearrangement is not an exception. The most evident correlation is the typical bending of colinear C1–N2 and C3–N2 bonds observed at point 57, implying a density transfer from the C1=N2 double bond toward the new non-bonding domain on top of N2. The opposite is observed at the end of phase VIII, when the formation of the new C3=N2 double bond is being completed. The same effect, although to a less extent, is observed in the allylic fragment.

**Figure 5:**
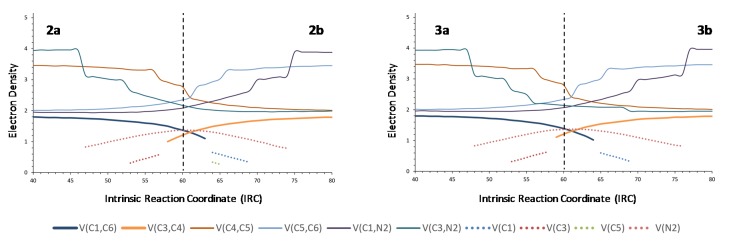
ELF analyses for the 2-aza-Cope rearrangement of nitrones **2** and **3**.

We then performed the ELF analyses of nitrones **2** and **3** bearing electron-attractor and electron-donor groups, respectively. A minimal asynchronicity could be detected in the formation/breaking of the C1–C6 and C3–C4 bonds involved in the rearrangement but the analyses were essentially identical to that of nitrone **1** (Figure 5), indicating that the process was scarcely sensitive to electronic factors.

**Figure 6:**
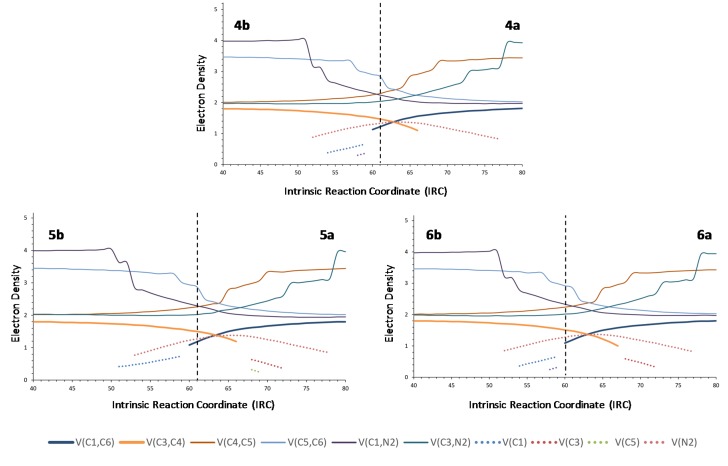
ELF analyses for the 2-aza-Cope rearrangement of nitrones **4**–**6**.

On the other hand, replacement of one aromatic ring by a methyl group (nitrone **4**) resulted in a clearly different ELF profile, which was found to be not so symmetrical like those of nitrones **1**–**3** (Figure 6). In fact, the C1–C6 bond is formed later than C3–C4 is broken, at a stage taking place after the TS. Replacement of the methyl group by a trifluoromethyl group (nitrone **5**) or a methoxymethyl group (nitrone **6**) provides almost identical profiles, reinforcing the idea that the process is sensitive to steric factors but not to electronic factors.

**Figure 7:**
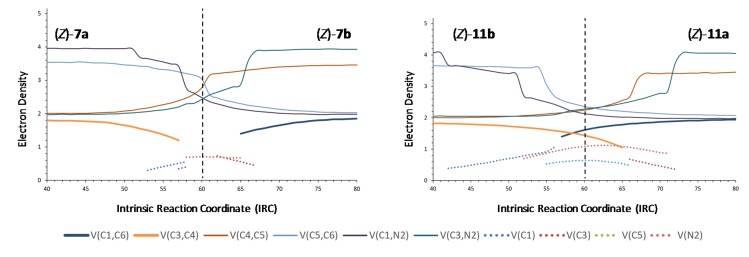
ELF analyses for the 2-aza-Cope rearrangement of nitrones (*Z*)-**7** and (*Z*)-**11**.

From the comparison between the BET analysis of the rearrangement of nitrone **1** and the ELF analyses of rearrangements of nitrones **2**–**6**, it becomes evident that substituents at the nitrone fragment exert little electronic influence, but exert a certain steric effect, although it is not very important. In order to study the effect of substituents at the allylic fragment, we carried out ELF analyses of nitrones **7**–**10**. Almost identical graphics were obtained for nitrones **8** and **9** (see supplementary information), although it can be appreciated that a slightly higher asynchronicity in forming C1–C6 and breaking C3–C4 bonds than for nitrones substituted at the nitrone fragment, the effect being more evident in (*Z*)-isomers. In the case of nitrone **10**, the most favorable for electron density transfer from the allylic fragment to the nitrone one, a similar minimal asynchronicity was observed. On the contrary, nitrone **7**, substituted with a *tert*-butyl group and, in particular, the (*Z*)-isomer showed the higher asynchronicity (Figure 7), demonstrating definitively the steric but not electronic dependence of the thermal neutral 2-aza-Cope rearrangements.

Nitrone **11**, in agreement with the above results, only showed little asynchronicity (more evident for the (*Z*)-isomer), despite bearing donor and attractor groups directly linked to the allylic and nitrone fragments, respectively. The ELF analysis of cyclic nitrones **12** and **13** also supported the steric dependence of the process: rearrangement of nitrone **13** resulted more asynchronous as a consequence of the presence of a quaternary center with more steric requirements than rearrangement of nitrone **12**.

## 4. Conclusions

In conclusion, the thermal neutral 2-aza-Cope rearrangement can be considered as a one-step process whose synchronicity depends, to some extent, on the steric requirements of the substituents, rather than their electronic features. BET and ELF analyses evaluate the evolution of electron density along the reaction coordinate and that evolution can depend of several factors including stereo- and electronic effects or a combination of both (stereoelectronic). By studying which factors (and to what extent) influence changes in electron density during the reaction, the main ones can be determined that can affect the studied process. In the case of 2-aza-Cope rearrangement of nitrones, ELF analyses of diversely substituted nitrones confirmed the steric dependence of the reaction. In fact, essentially no changes have been observed in the evolution of electron density (that tell us how bonds are formed and broken) for nitrones substituted with electronically very different substituents. On the other hand, clearly visible changes in the evolution of electron density are observed when substituents with very different steric requirement are introduced. According to the GEDT, the process is identified as non-polar and only in a few cases with a particular substitution a low polar process is detected. The BET analysis settles the pseudoradical character of the process, confirming the absence of a pericyclic electron-reorganization. This conclusion can be extended to all the cases studied, independent of the substituents.

## Figures and Tables

**Table 1:** 2-Aza-Cope rearrangement of γ-Alkenyl Nitrones ^a^.

Nitrone ^b^	R^1^	R^2^	R^3^	R^4^	Barrier ^c^	GEDT ^d^	C1–C6 ^e^	C3–C4 ^e^
**1**	Ph	Ph	H	H	32.7	0.09	1.78	1.78
**2**	Ph	4-NO_2_C_6_H_4_	H	H	32.9	0.11	1.79	1.78
**3**	Ph	4-MeOC_6_H_4_	H	H	33.5	0.08	1.78	1.79
**4**	Ph	Me	H	H	28.6	0.06	1.71	1.84
**5**	Ph	CF_3_	H	H	26.8	0.11	1.69	1.88
**6**	Ph	CH_2_OMe	H	H	27.5	0.08	1.70	1.85
(*E*)-**7**	Ph	Ph	tBu	H	43.6	0.10	1.88	1.88
(*Z*)-**7**	Ph	Ph	tBu	H	46.5	0.10	2.17	2.13
(*E*)-**8**	Ph	Ph	OMe	H	36.9	0.11	1.88	1.94
(*Z*)-**8**	Ph	Ph	OMe	H	42.4	0.05	1.84	1.84
(*E*)-**9**	Ph	Ph	NO_2_	H	36.0	−0.02	1.84	1.88
(*Z*)-**9**	Ph	Ph	NO_2_	H	43.0	−0.08	1.87	1.88
**10**	Ph	Ph	H	OMe	34.1	0.17	1.86	1.68
(*E*)-**11**	NO_2_	Me	OMe	H	31.9	0.14	1.66	1.79
(*Z*)-**11**	NO_2_	Me	OMe	H	39.1	0.08	1.66	1.79
**12**	H	COOMe			34.4	0.07	1.79	1.70
**13**	CH_2_OMe	COOMe			30.9	0.08	1.75	1.75

^a^ Referred to **a** series. ^b^ All nitrones considered as the most stable (*Z*)-isomers. (*E*)- and (*Z*)- refers to configuration of the alkenyl moiety. ^c^ Given in kcal/mol. ^d^ Defined as the difference in electron charge between the nitrone and allyl fragments at TS. Given in e. ^e^ Distances in angstrom.

**Table 2:** Valence basin populations calculated from the ELF of the IRC points, defining the nine SSDs characterizing the molecular 2-aza-Cope rearrangement of **1**. The stationary points **1** and **TS1** are also included. Distances are given in Å.

	P1f ^a^		P47		P53		P57		P58		P64		P65		P69		P75		P121 ^a^	TS ^b^
		*I*		*II*		*III*		*IV*		*V*		*VI*		*VII*		*VIII*		*IX*		
**d(C1,C6)**	3.654		2.405		2.128		1.944		1.899		1.702		1.685		1.633		1.590		1.552	1.781
**d(C3,C4)**	1.552		1.590		1.634		1.685		1.702		1.890		1.944		2.129		2.405		3.654	1.782
**d(C1,N2)**	1.316		1.323		1.346		1.376		1.385		1.434		1.44		1.461		1.483		1.506	1.411
**d(N2,C3)**	1.506		1.483		1.461		1.441		1.434		1.385		1.376		1.347		1.323		1.316	1.412
**d(C5,C6)**	1.333		1.343		1.362		1.389		1.397		1.448		1.454		1.476		1.494		1.506	1.424
**d(C4,C5)**	1.506		1.494		1.475		1.454		1.447		1.397		1.389		1.362		1.342		1.333	1.424
**V(C1,C6)**									1.01		1.50		1.54		1.66		1.76		1.85	1.31
**V(C3,C4)**	1.85		1.76		1.66		1.54		1.49		1.01									1.31
**V1(C4,C5)**	1.99		2.04		2.12		2.22		2.26		2.93		3.04		3.34		1.67		1.75	2.42
**V2(C4,C5)**																			1.76	
**V1(C5,C6)**	1.75		1.67		3.34		3.02		2.94		2.26		2.22		2.12		2.04		1.99	2.42
**V2(C5,C6)**	1.76		1.76																	
**V(C1,N2)**	3.96		3.14		2.64		2.35		2.29		2.02		2.00		1.96		1.94		1.98	2.13
**V(C3,N2)**	1.98		1.94		1.96		2.00		2.02		2.29		2.36		2.64		3.14		3.96	2.13
**V(C1)**					0.33				0.59											
**V(N2)**			0.82		1.12		1.30		1.33		1.33		1.30		1.12		0.82			1.38
**V(C3)**													0.33		0.59					
**V(C4)**													0.20							
**V(C5)**									0.29											
**V(C6)**																				0.29

^a^ Corresponding to nitrone **1**. ^b^ Corresponding to **P61**.

## Supplementary Materials

The following are available online at www.mdpi.com/1420-3049/22/8/1371/s1, Energy data; full ELF analyses including graphics and basins populations. Transition structures.
