# Supplementary material for: BET & ELF Quantum Topological Analysis of Neutral 2-Aza-Cope Rearrangement of γ-Alkenyl Nitrones
Source: Molecules. 2017 Aug 19;22(8):1371. doi: 10.3390/molecules22081371 (PMC6151987; doi:10.3390/molecules22081371)
Supplement: Supplementary file 1 [file molecules-22-01371-s001.pdf]

# BET & ELF Quantum Topological Analysis of Neutral 2-Aza-Cope Rearrangement of $\gamma$-Alkenyl Nitrones

Pedro Merino,[a*] Maria A. Chiacchio,[b] Laura Legnani,[c] Tomas Tejero,[d]

[a] *Instituto de Biocomputación y Física de Sistemas Complejos (BIFI), Universidad de Zaragoza, Zaragoza, 50009, Spain*

[b] *Dipartimento di Scienze del Farmaco, Università di Catania, Catania, Italy*

[c] *Dipartimento di Chimica, Università di Pavia, Via Taramelli 12, 27100 Pavia, Italy*

[d] *Instituto de Síntesis Química y Catalisis Homogénea (ISQCH), Universidad de Zaragoza-CSIC, Zaragoza, 50009, Spain.*

e-mail: pmerino@unizar.es

http://www.pmerino.com

## Electronic Supporting Information

Transition structures ........................................................................................S2

Electronic populations ........................................................................................S4

Graphical representations of ELF analyses...........................................................S21

Cartesian coordinates........................................................................................ S

## Transition Structures

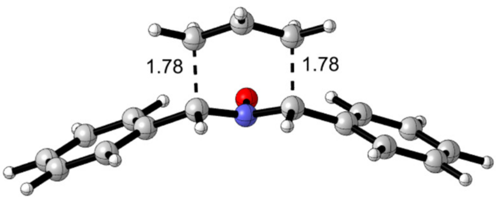


**TS01**

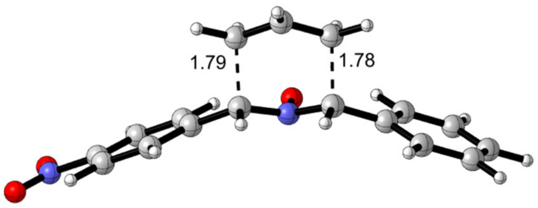


**TS02**

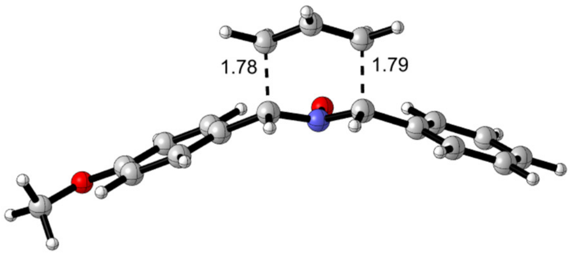


**TS03**

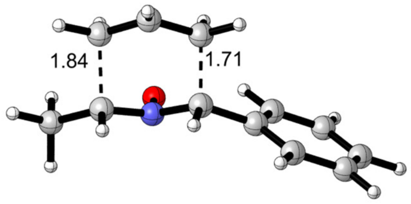


**TS04**

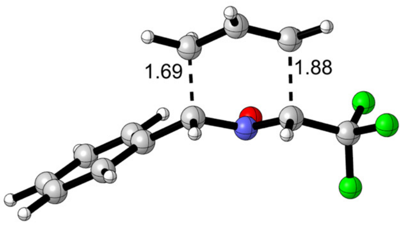


**TS05**

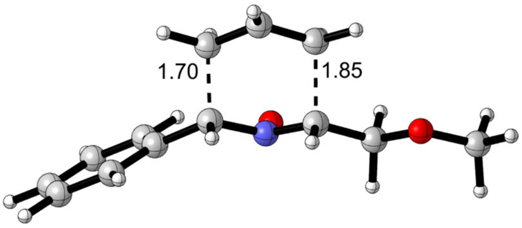


**TS06**

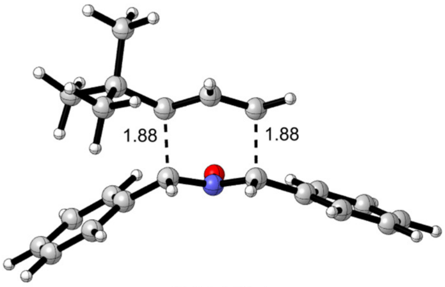


(*E*)-**TS07**

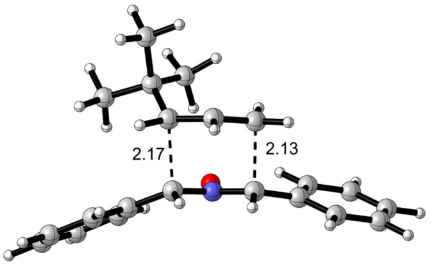


(*Z*)-**TS07**

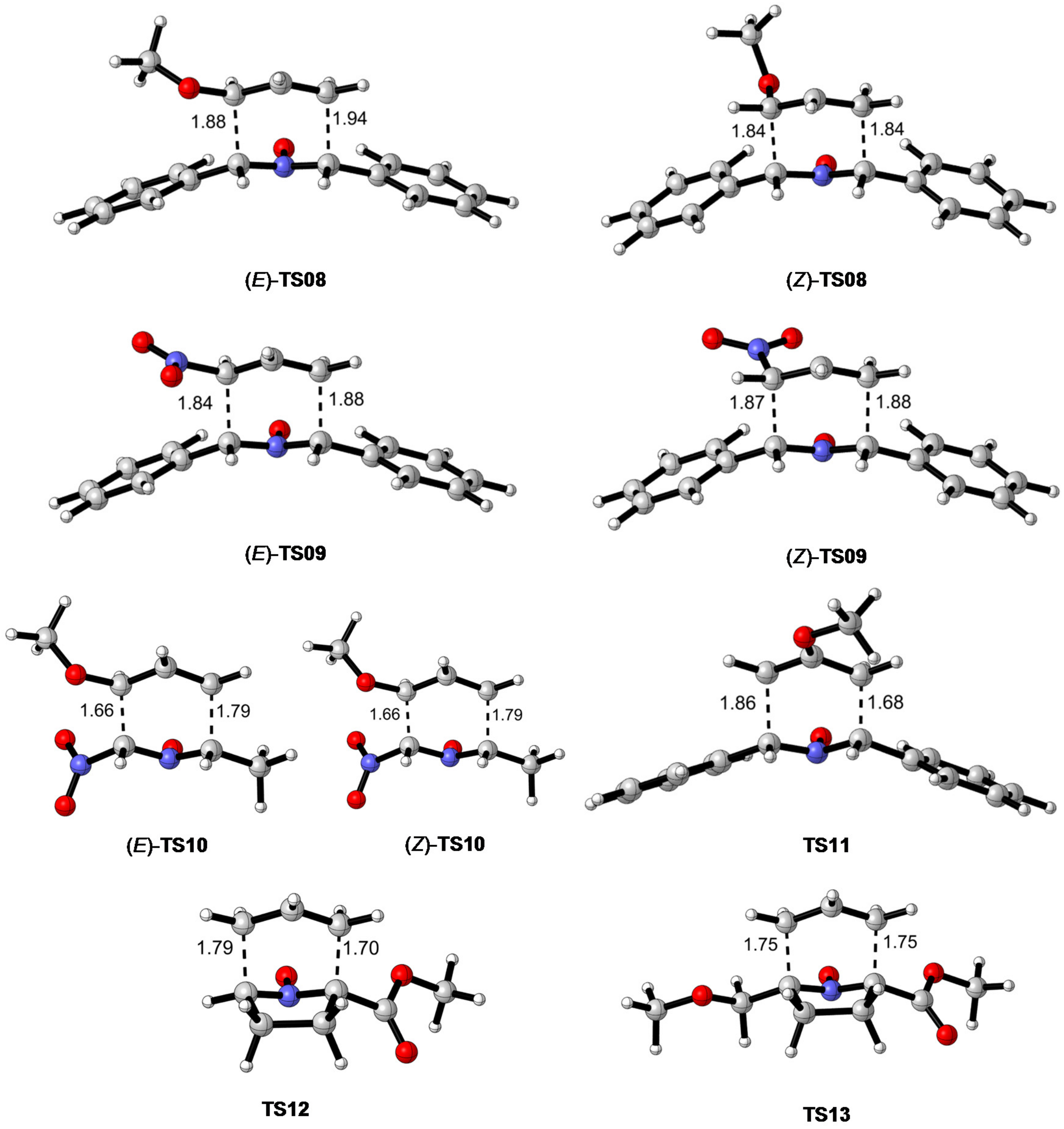


**Figure S2.** Transition structures for 2-aza-Cope rearrangements

**Electronic populations** (only 25 points before and 25 points after the TS are listed)

Table S1. Electronic population of basins for the rearrangement of nitrone **1**

| IRC | V(C1,C6) | V(C3,C4) | V1(C4,C5) | V2(C4C5) | V1C5,C6) | V2(C5,C6) | V1(C1,N2) | V2(C1,N2) | V1(C3,N2) | V2(C3,N2) | V(N2) | V(C1) | V(C3) | V(C4) | V(C5) | V(C6) |
|---|---|---|---|---|---|---|---|---|---|---|---|---|---|---|---|---|
| 36 | - | 1.81 | 2.00 | - | 1.77 | 1.71 | 3.94 | - | 1.96 | - | - | - | - | - | - | - |
| 37 | - | 1.81 | 2.00 | - | 1.77 | 1.71 | 3.94 | - | 1.96 | - | - | - | - | - | - | - |
| 38 | - | 1.81 | 2.01 | - | 1.77 | 1.71 | 3.93 | - | 1.97 | - | - | - | - | - | - | - |
| 39 | - | 1.80 | 2.01 | - | 1.76 | 1.71 | 3.93 | - | 1.96 | - | - | - | - | - | - | - |
| 40 | - | 1.80 | 2.01 | - | 1.77 | 1.70 | 3.94 | - | 1.97 | - | - | - | - | - | - | - |
| 41 | - | 1.79 | 2.01 | - | 1.77 | 1.70 | 3.93 | - | 1.96 | - | - | - | - | - | - | - |
| 42 | - | 1.79 | 2.01 | - | 1.77 | 1.70 | 3.93 | - | 1.95 | - | - | - | - | - | - | - |
| 43 | - | 1.78 | 2.02 | - | 1.77 | 1.69 | 3.94 | - | 1.96 | - | - | - | - | - | - | - |
| 44 | - | 1.78 | 2.02 | - | 1.77 | 1.68 | 3.94 | - | 1.95 | - | - | - | - | - | - | - |
| 45 | - | 1.77 | 2.03 | - | 1.76 | 1.68 | 3.95 | - | 1.94 | - | - | - | - | - | - | - |
| 46 | - | 1.77 | 2.03 | - | 1.76 | 1.67 | 3.95 | - | 1.94 | - | - | - | - | - | - | - |
| 47 | - | 1.76 | 2.04 | - | 1.67 | 1.76 | 3.14 | - | 1.94 | - | 0.82 | - | - | - | - | - |
| 48 | - | 1.75 | 2.04 | - | 1.66 | 1.76 | 3.13 | - | 1.93 | - | 0.85 | - | - | - | - | - |
| 49 | - | 1.73 | 2.06 | - | 1.65 | 1.75 | 3.08 | - | 1.94 | - | 0.91 | - | - | - | - | - |
| 50 | - | 1.72 | 2.07 | - | 1.64 | 1.76 | 3.05 | - | 1.94 | - | 0.96 | - | - | - | - | - |
| 51 | - | 1.70 | 2.08 | - | 1.62 | 1.76 | 3.01 | - | 1.94 | - | 1.02 | - | - | - | - | - |
| 52 | - | 1.68 | 2.10 | - | 1.60 | 1.77 | 2.98 | - | 1.95 | - | 1.07 | - | - | - | - | - |
| 53 | - | 1.66 | 2.12 | - | 3.34 | - | 2.64 | - | 1.96 | - | 1.12 | 0.33 | - | - | - | - |
| 54 | - | 1.63 | 2.14 | - | 3.33 | - | 2.55 | - | 1.96 | - | 1.18 | 0.40 | - | - | - | - |
| 55 | - | 1.60 | 2.16 | - | 3.33 | - | 2.48 | - | 1.97 | - | 1.22 | 0.47 | - | - | - | - |
| 56 | - | 1.57 | 2.19 | - | 3.32 | - | 2.41 | - | 1.98 | - | 1.27 | 0.53 | - | - | - | - |
| 57 | - | 1.54 | 2.22 | - | 3.02 | - | 2.35 | - | 2.00 | - | 1.30 | 0.59 | - | - | 0.29 | - |
| 58 | 1.01 | 1.49 | 2.26 | - | 2.94 | - | 2.29 | - | 2.02 | - | 1.33 | - | - | - | - | - |
| 59 | 1.12 | 1.44 | 2.30 | - | 2.85 | - | 2.24 | - | 2.05 | - | 1.36 | - | - | - | - | - |
| 60 | 1.22 | 1.38 | 2.35 | - | 2.78 | - | 2.18 | - | 2.08 | - | 1.37 | - | - | - | - | - |
| 61 | 1.31 | 1.31 | 2.42 | - | 2.42 | - | 2.13 | - | 2.13 | - | 1.38 | - | - | - | - | 0.29 |
| 62 | 1.39 | 1.22 | 2.78 | - | 2.35 | - | 2.08 | - | 2.18 | - | 1.37 | - | - | - | - | - |
| 63 | 1.44 | 1.12 | 2.85 | - | 2.30 | - | 2.04 | - | 2.24 | - | 1.36 | - | - | - | - | - |
| 64 | 1.50 | 1.01 | 2.93 | - | 2.26 | - | 2.02 | - | 2.29 | - | 1.33 | - | - | - | - | - |
| 65 | 1.54 | - | 3.04 | - | 2.22 | - | 2.00 | - | 2.36 | - | 1.30 | - | 0.33 | 0.20 | - | - |
| 66 | 1.57 | - | 3.32 | - | 2.19 | - | 1.98 | - | 2.42 | - | 1.26 | - | 0.40 | - | - | - |
| 67 | 1.61 | - | 3.33 | - | 2.16 | - | 1.97 | - | 2.48 | - | 1.22 | - | 0.47 | - | - | - |
| 68 | 1.63 | - | 3.33 | - | 2.14 | - | 1.96 | - | 2.55 | - | 1.18 | - | 0.53 | - | - | - |
| 69 | 1.66 | - | 3.34 | - | 2.12 | - | 1.96 | - | 2.64 | - | 1.12 | - | 0.59 | - | - | - |
| 70 | 1.68 | - | 1.60 | 1.77 | 2.10 | - | 1.95 | - | 2.98 | - | 1.07 | - | - | - | - | - |
| 71 | 1.70 | - | 1.62 | 1.77 | 2.08 | - | 1.95 | - | 3.01 | - | 1.01 | - | - | - | - | - |
| 72 | 1.72 | - | 1.64 | 1.76 | 2.07 | - | 1.94 | - | 3.04 | - | 0.96 | - | - | - | - | - |
| 73 | 1.73 | - | 1.65 | 1.76 | 2.06 | - | 1.94 | - | 3.08 | - | 0.91 | - | - | - | - | - |
| 74 | 1.75 | - | 1.66 | 1.76 | 2.05 | - | 1.93 | - | 3.13 | - | 0.85 | - | - | - | - | - |
| 75 | 1.76 | - | 1.67 | 1.76 | 2.04 | - | 1.94 | - | 3.14 | - | 0.82 | - | - | - | - | - |
| 76 | 1.77 | - | 1.67 | 1.76 | 2.03 | - | 1.94 | - | 3.95 | - | - | - | - | - | - | - |
| 77 | 1.77 | - | 1.68 | 1.76 | 2.03 | - | 1.94 | - | 3.95 | - | - | - | - | - | - | - |

| | | | | | | | | | | | | | | | | |
|---|---|---|---|---|---|---|---|---|---|---|---|---|---|---|---|---|
| 78 | 1.78 | - | 1.69 | 1.76 | 2.02 | - | 1.95 | - | 3.95 | - | - | - | - | - | - | - |
| 79 | 1.78 | - | 1.69 | 1.77 | 2.02 | - | 1.95 | - | 3.95 | - | - | - | - | - | - | - |
| 80 | 1.79 | - | 1.70 | 1.76 | 2.01 | - | 1.95 | - | 3.94 | - | - | - | - | - | - | - |
| 81 | 1.79 | - | 1.70 | 1.77 | 2.01 | - | 1.96 | - | 3.94 | - | - | - | - | - | - | - |
| 82 | 1.80 | - | 1.70 | 1.77 | 2.01 | - | 1.97 | - | 3.93 | - | - | - | - | - | - | - |
| 83 | 1.80 | - | 1.71 | 1.76 | 2.01 | - | 1.97 | - | 3.93 | - | - | - | - | - | - | - |
| 84 | 1.81 | - | 1.71 | 1.77 | 2.01 | - | 1.97 | - | 3.94 | - | - | - | - | - | - | - |
| 85 | 1.81 | - | 1.71 | 1.77 | 2.00 | - | 1.96 | - | 3.94 | - | - | - | - | - | - | - |
| 86 | 1.81 | - | 1.72 | 1.77 | 2.00 | - | 1.96 | - | 3.94 | - | - | - | - | - | - | - |

**Table S2.** Electronic population of basins for the rearrangement of nitrones **2**

| IRC | V(C1,C6) | V(C3,C4) | V1(C4,C5) | V2(C4C5) | V1C5,C6) | V2(C5,C6) | V1(C1,N2) | V2(C1,N2) | V1(C3,N2) | V2(C3,N2) | V(N2) | V(C1) | V(C3) | V(C4) | V(C5) | V(C6) |
|---|---|---|---|---|---|---|---|---|---|---|---|---|---|---|---|---|
| 36 | 1.81 | - | 1.71 | 1.76 | 2.00 | - | 1.96 | - | 3.95 | - | - | - | - | - | - | - |
| 37 | 1.80 | - | 1.71 | 1.76 | 2.01 | - | 1.96 | - | 3.95 | - | - | - | - | - | - | - |
| 38 | 1.80 | - | 1.71 | 1.77 | 2.01 | - | 1.95 | - | 3.95 | - | - | - | - | - | - | - |
| 39 | 1.80 | - | 1.70 | 1.77 | 2.00 | - | 1.95 | - | 3.95 | - | - | - | - | - | - | - |
| 40 | 1.80 | - | 1.76 | 1.70 | 2.01 | - | 1.95 | - | 3.94 | - | - | - | - | - | - | - |
| 41 | 1.79 | - | 1.70 | 1.76 | 2.01 | - | 1.94 | - | 3.96 | - | - | - | - | - | - | - |
| 42 | 1.78 | - | 1.69 | 1.76 | 2.01 | - | 1.94 | - | 3.95 | - | - | - | - | - | - | - |
| 43 | 1.78 | - | 1.68 | 1.76 | 2.02 | - | 1.94 | - | 3.96 | - | - | - | - | - | - | - |
| 44 | 1.77 | - | 1.68 | 1.77 | 2.02 | - | 1.94 | - | 3.96 | - | - | - | - | - | - | - |
| 45 | 1.77 | - | 1.68 | 1.76 | 2.02 | - | 1.94 | - | 3.96 | - | - | - | - | - | - | - |
| 46 | 1.76 | - | 1.67 | 1.76 | 2.03 | - | 1.94 | - | 3.95 | - | - | - | - | - | - | - |
| 47 | 1.75 | - | 1.76 | 1.66 | 2.03 | - | 1.94 | - | 3.14 | - | - | - | - | - | - | - |
| 48 | 1.74 | - | 1.76 | 1.65 | 2.04 | - | 1.93 | - | 3.10 | - | - | - | - | - | - | - |
| 49 | 1.73 | - | 1.76 | 1.64 | 2.05 | - | 1.94 | - | 3.06 | - | - | - | - | - | - | - |
| 50 | 1.71 | - | 1.76 | 1.62 | 2.06 | - | 1.94 | - | 3.02 | - | - | - | - | - | - | - |
| 51 | 1.69 | - | 1.76 | 1.61 | 2.08 | - | 1.94 | - | 2.99 | - | - | - | - | - | - | - |
| 52 | 1.67 | - | 1.77 | 1.58 | 2.09 | - | 1.94 | - | 2.97 | - | - | - | - | - | - | - |
| 53 | 1.65 | - | 3.32 | - | 2.12 | - | 1.95 | - | 2.65 | - | - | - | 0.31 | - | - | - |
| 54 | 1.63 | - | 3.31 | - | 2.13 | - | 1.95 | - | 2.56 | - | - | - | 0.39 | - | - | - |
| 55 | 1.60 | - | 3.31 | - | 2.15 | - | 1.97 | - | 2.48 | - | - | - | 0.45 | - | - | - |
| 56 | 1.58 | - | 3.31 | - | 2.18 | - | 1.98 | - | 2.42 | - | - | - | 0.52 | - | - | - |
| 57 | 1.54 | - | 3.01 | - | 2.21 | - | 2.00 | - | 2.35 | - | - | - | 0.59 | 0.28 | - | - |
| 58 | 1.50 | 1.00 | 2.92 | - | 2.25 | - | 2.02 | - | 2.29 | - | - | - | - | - | - | - |
| 59 | 1.43 | 1.11 | 2.84 | - | 2.30 | - | 2.05 | - | 2.23 | - | - | - | - | - | - | - |
| 60 | 1.37 | 1.21 | 2.77 | - | 2.35 | - | 2.08 | - | 2.17 | - | - | - | - | - | - | - |
| 61 | 1.30 | 1.30 | 2.43 | - | 2.43 | - | 2.14 | - | 2.12 | - | - | - | - | - | - | 0.26 |
| 62 | 1.21 | 1.37 | 2.35 | - | 2.77 | - | 2.19 | - | 2.07 | - | - | - | - | - | - | - |
| 63 | 1.10 | 1.43 | 2.30 | - | 2.84 | - | 2.25 | - | 2.04 | - | - | - | - | - | - | - |
| 64 | - | 1.49 | 2.25 | - | 2.93 | - | 2.30 | - | 2.02 | - | - | 0.65 | - | - | 0.34 | - |
| 65 | - | 1.53 | 2.22 | - | 3.02 | - | 2.36 | - | 2.00 | - | - | 0.59 | - | - | 0.27 | - |
| 66 | - | 1.57 | 2.18 | - | 3.30 | - | 2.43 | - | 1.99 | - | - | 0.52 | - | - | - | - |
| 67 | - | 1.60 | 2.16 | - | 3.31 | - | 2.49 | - | 1.98 | - | - | 0.46 | - | - | - | - |
| 68 | - | 1.63 | 2.14 | - | 3.31 | - | 2.56 | - | 1.97 | - | - | 0.40 | - | - | - | - |
| 69 | - | 1.66 | 2.11 | - | 3.32 | - | 2.65 | - | 1.97 | - | - | 0.33 | - | - | - | - |
| 70 | - | 1.68 | 2.10 | - | 1.77 | 1.58 | 2.99 | - | 1.97 | - | 0.83 | - | - | - | - | - |

| | | | | | | | | | | | | | | | | |
|---|---|---|---|---|---|---|---|---|---|---|---|---|---|---|---|---|
| 71 | - | 1.70 | 2.08 | - | 1.76 | 1.62 | 3.01 | - | 1.97 | - | 0.88 | - | - | - | - | - |
| 72 | - | 1.72 | 2.07 | - | 1.75 | 1.63 | 3.06 | - | 1.96 | - | 0.93 | - | - | - | - | - |
| 73 | - | 1.73 | 2.06 | - | 1.75 | 1.64 | 3.09 | - | 1.97 | - | 0.99 | - | - | - | - | - |
| 74 | - | 1.74 | 2.05 | - | 1.76 | 1.65 | 3.12 | - | 1.97 | - | 1.04 | - | - | - | - | - |
| 75 | - | 1.75 | 2.04 | - | 1.66 | 1.76 | 3.89 | - | 1.98 | - | 1.09 | - | - | - | - | - |
| 76 | - | 1.76 | 2.03 | - | 1.67 | 1.76 | 3.89 | - | 1.97 | - | 1.14 | - | - | - | - | - |
| 77 | - | 1.77 | 2.03 | - | 1.67 | 1.76 | 3.89 | - | 1.98 | - | 1.19 | - | - | - | - | - |
| 78 | - | 1.78 | 2.02 | - | 1.68 | 1.76 | 3.89 | - | 1.98 | - | 1.23 | - | - | - | - | - |
| 79 | - | 1.79 | 2.02 | - | 1.69 | 1.76 | 3.88 | - | 1.98 | - | 1.27 | - | - | - | - | - |
| 80 | - | 1.79 | 2.01 | - | 1.69 | 1.76 | 3.88 | - | 1.99 | - | 1.30 | - | - | - | - | - |
| 81 | - | 1.79 | 2.01 | - | 1.69 | 1.76 | 3.87 | - | 1.99 | - | 1.33 | - | - | - | - | - |
| 82 | - | 1.80 | 2.01 | - | 1.70 | 1.76 | 3.88 | - | 1.98 | - | 1.36 | - | - | - | - | - |
| 83 | - | 1.80 | 2.02 | - | 1.70 | 1.77 | 3.87 | - | 1.99 | - | 1.37 | - | - | - | - | - |
| 84 | - | 1.81 | 2.01 | - | 1.71 | 1.76 | 3.88 | - | 1.99 | - | 1.37 | - | - | - | - | - |
| 85 | - | 1.81 | 2.00 | - | 1.71 | 1.76 | 3.88 | - | 1.99 | - | 1.36 | - | - | - | - | - |
| 86 | - | 1.81 | 2.01 | - | 1.71 | 1.77 | 3.88 | - | 2.00 | - | 1.34 | - | - | - | - | - |

**Table S3**. Electronic population of basins for the rearrangement of nitrones **3**

| IRC | V(C1,C6) | V(C3,C4) | V1(C4,C5) | V2(C4C5) | V1C5,C6) | V2(C5,C6) | V1(C1,N2) | V2(C1,N2) | V1(C3,N2) | V2(C3,N2) | V(N2) | V(C1) | V(C3) | V(C4) | V(C5) | V(C6) |
|---|---|---|---|---|---|---|---|---|---|---|---|---|---|---|---|---|
| 36 | 1.82 | - | 1.71 | 1.77 | 2.01 | - | 1.96 | | 3.94 | | - | - | - | - | - | - |
| 37 | 1.81 | - | 1.71 | 1.77 | 2.01 | - | 1.96 | | 3.93 | | - | - | - | - | - | - |
| 38 | 1.81 | - | 1.70 | 1.77 | 2.01 | - | 1.97 | | 3.93 | | - | - | - | - | - | - |
| 39 | 1.81 | - | 1.70 | 1.77 | 2.02 | - | 1.96 | | 3.94 | | - | - | - | - | - | - |
| 40 | 1.80 | - | 1.70 | 1.77 | 2.02 | - | 1.96 | | 3.93 | | - | - | - | - | - | - |
| 41 | 1.80 | - | 1.70 | 1.77 | 2.01 | - | 1.97 | | 3.93 | | - | - | - | - | - | - |
| 42 | 1.79 | - | 1.70 | 1.77 | 2.01 | - | 1.96 | | 3.93 | | - | - | - | - | - | - |
| 43 | 1.79 | - | 1.69 | 1.76 | 2.02 | - | 1.96 | | 3.93 | | - | - | - | - | - | - |
| 44 | 1.78 | - | 1.69 | 1.77 | 2.02 | - | 1.95 | | 3.95 | | - | - | - | - | - | - |
| 45 | 1.78 | - | 1.68 | 1.76 | 2.02 | - | 1.95 | | 3.95 | | - | - | - | - | - | - |
| 46 | 1.77 | - | 1.67 | 1.76 | 2.03 | - | 1.95 | | 3.93 | | - | - | - | - | - | - |
| 47 | 1.76 | - | 1.67 | 1.76 | 2.04 | - | 1.95 | | 3.93 | | - | - | - | - | - | - |
| 48 | 1.75 | - | 1.66 | 1.76 | 2.04 | - | 1.95 | | 3.13 | | 0.83 | - | - | - | - | - |
| 49 | 1.74 | - | 1.76 | 1.65 | 2.05 | - | 1.95 | | 3.09 | | 0.88 | - | - | - | - | - |
| 50 | 1.72 | - | 1.76 | 1.64 | 2.06 | - | 1.95 | | 3.05 | | 0.94 | - | - | - | - | - |
| 51 | 1.71 | - | 1.76 | 1.62 | 2.08 | - | 1.95 | | 3.01 | | 0.99 | - | - | - | - | - |
| 52 | 1.69 | - | 1.78 | 1.59 | 2.09 | - | 1.95 | | 2.99 | | 1.04 | - | - | - | - | - |
| 53 | 1.66 | - | 3.33 | - | 2.11 | - | 1.95 | | 2.65 | | 1.10 | - | 0.32 | - | - | - |
| 54 | 1.64 | - | 3.33 | - | 2.13 | - | 1.96 | | 2.57 | | 1.15 | - | 0.38 | - | - | - |
| 55 | 1.61 | - | 3.33 | - | 2.15 | - | 1.97 | | 2.49 | | 1.20 | - | 0.45 | - | - | - |
| 56 | 1.58 | - | 3.32 | - | 2.18 | - | 1.98 | | 2.22 | | 1.25 | - | 0.52 | - | - | - |
| 57 | 1.55 | - | 3.04 | - | 2.21 | - | 1.99 | | 2.20 | | 1.29 | - | 0.58 | 0.27 | - | - |
| 58 | 1.50 | - | 2.95 | - | 2.24 | - | 2.01 | | 2.18 | | 1.33 | - | 0.64 | 0.34 | - | - |
| 59 | 1.45 | 1.11 | 2.88 | - | 2.29 | - | 2.04 | | 2.16 | | 1.35 | - | - | - | - | - |
| 60 | 1.39 | 1.21 | 2.80 | - | 2.34 | - | 2.07 | | 2.14 | | 1.36 | - | - | - | - | - |
| 61 | 1.32 | 1.30 | 2.43 | - | 2.41 | - | 2.11 | | 2.12 | | 1.37 | - | - | - | - | 0.30 |

| | | | | | | | | | | | | | | | | |
|---|---|---|---|---|---|---|---|---|---|---|---|---|---|---|---|---|
| 62 | 1.23 | 1.37 | 2.36 | - | 2.78 | - | 2.17 | | 2.11 | | 1.37 | - | - | - | - | - |
| 63 | 1.14 | 1.44 | 2.30 | - | 2.85 | - | 2.22 | | 2.10 | | 1.36 | - | - | - | - | - |
| 64 | 1.02 | 1.49 | 2.25 | - | 2.93 | - | 2.28 | | 2.09 | | 1.34 | - | - | - | - | - |
| 65 | - | 1.54 | 2.21 | - | 3.01 | - | 2.33 | | 2.08 | | 1.31 | 0.60 | - | - | 0.30 | - |
| 66 | - | 1.57 | 2.18 | - | 3.32 | - | 2.40 | | 2.08 | | 1.28 | 0.53 | - | - | - | - |
| 67 | - | 1.60 | 2.15 | - | 3.33 | - | 2.46 | | 2.08 | | 1.24 | 0.47 | - | - | - | - |
| 68 | - | 1.63 | 2.13 | - | 3.32 | - | 2.54 | | 2.07 | | 1.20 | 0.40 | - | - | - | - |
| 69 | - | 1.66 | 2.12 | - | 3.34 | - | 2.62 | | 1.95 | | 1.15 | 0.32 | - | - | - | - |
| 70 | - | 1.69 | 2.09 | - | 1.78 | 1.59 | 2.96 | | 1.95 | | 1.10 | - | - | - | - | - |
| 71 | - | 1.71 | 2.08 | - | 1.77 | 1.62 | 2.98 | | 1.95 | | 1.05 | - | - | - | - | - |
| 72 | - | 1.72 | 2.07 | - | 1.76 | 1.63 | 3.01 | | 1.94 | | 1.00 | - | - | - | - | - |
| 73 | - | 1.73 | 2.06 | - | 1.76 | 1.65 | 3.04 | | 1.94 | | 0.95 | - | - | - | - | - |
| 74 | - | 1.74 | 2.05 | - | 1.76 | 1.66 | 3.08 | | 1.94 | | 0.90 | - | - | - | - | - |
| 75 | - | 1.76 | 2.04 | - | 1.76 | 1.66 | 3.13 | | 1.94 | | 0.85 | - | - | - | - | - |
| 76 | - | 1.76 | 2.03 | - | 1.77 | 1.67 | 3.14 | | 1.94 | | 0.82 | - | - | - | - | - |
| 77 | - | 1.77 | 2.03 | - | 1.68 | 1.77 | 3.95 | | 1.95 | | - | - | - | - | - | - |
| 78 | - | 1.78 | 2.02 | - | 1.69 | 1.77 | 3.96 | | 1.95 | | - | - | - | - | - | - |
| 79 | - | 1.79 | 2.02 | - | 1.69 | 1.77 | 3.96 | | 1.95 | | - | - | - | - | - | - |
| 80 | - | 1.79 | 2.01 | - | 1.69 | 1.77 | 3.96 | | 1.95 | | - | - | - | - | - | - |
| 81 | - | 1.80 | 2.01 | - | 1.70 | 1.77 | 3.96 | | 1.95 | | - | - | - | - | - | - |
| 82 | - | 1.80 | 2.01 | - | 1.77 | 1.70 | 3.95 | | 1.95 | | - | - | - | - | - | - |
| 83 | - | 1.80 | 2.01 | - | 1.77 | 1.70 | 3.96 | | 1.95 | | - | - | - | - | - | - |
| 84 | - | 1.81 | 2.01 | - | 1.77 | 1.71 | 3.96 | | 1.95 | | - | - | - | - | - | - |
| 85 | - | 1.81 | 2.01 | - | 1.77 | 1.71 | 3.96 | | 1.95 | | - | - | - | - | - | - |
| 86 | - | 1.81 | 2.01 | - | 1.77 | 1.71 | 3.96 | | 1.96 | | - | - | - | - | - | - |

**Table S4**. Electronic population of basins for the rearrangement of nitrones **4**

| **IRC** | **V(C1,C6)** | **V(C3,C4)** | **V1(C4,C5)** | **V2(C4C5)** | **V1C5,C6)** | **V2(C5,C6)** | **V1(C1,N2)** | **V2(C1,N2)** | **V1(C3,N2)** | **V2(C3,N2)** | **V(N2)** | **V(C1)** | **V(C3)** | **V(C4)** | **V(C5)** | **V(C6)** |
|---|---|---|---|---|---|---|---|---|---|---|---|---|---|---|---|---|
| 36 | - | 1.82 | 2.01 | - | 1.72 | 1.77 | 3.98 | | 1.98 | | - | - | - | - | - | - |
| 37 | - | 1.81 | 2.01 | - | 1.72 | 1.76 | 3.98 | | 1.98 | | - | - | - | - | - | - |
| 38 | - | 1.81 | 2.01 | - | 1.72 | 1.76 | 3.98 | | 1.98 | | - | - | - | - | - | - |
| 39 | - | 1.81 | 2.01 | - | 1.77 | 1.71 | 3.98 | | 1.98 | | - | - | - | - | - | - |
| 40 | - | 1.80 | 2.01 | - | 1.71 | 1.76 | 3.98 | | 1.97 | | - | - | - | - | - | - |
| 41 | - | 1.80 | 2.01 | - | 1.71 | 1.76 | 3.98 | | 1.97 | | - | - | - | - | - | - |
| 42 | - | 1.80 | 2.02 | - | 1.70 | 1.76 | 3.98 | | 1.98 | | - | - | - | - | - | - |
| 43 | - | 1.79 | 2.02 | - | 1.70 | 1.76 | 3.98 | | 1.97 | | - | - | - | - | - | - |
| 44 | - | 1.79 | 2.02 | - | 1.70 | 1.76 | 3.98 | | 1.97 | | - | - | - | - | - | - |
| 45 | - | 1.78 | 2.03 | - | 1.69 | 1.76 | 3.99 | | 1.97 | | - | - | - | - | - | - |
| 46 | - | 1.78 | 2.03 | - | 1.69 | 1.76 | 4.00 | | 1.97 | | - | - | - | - | - | - |
| 47 | - | 1.77 | 2.04 | - | 1.68 | 1.75 | 3.99 | | 1.96 | | - | - | - | - | - | - |
| 48 | - | 1.76 | 2.05 | - | 1.67 | 1.75 | 4.00 | | 1.96 | | - | - | - | - | - | - |
| 49 | - | 1.75 | 2.05 | - | 1.67 | 1.75 | 4.01 | | 1.96 | | - | - | - | - | - | - |
| 50 | - | 1.74 | 2.06 | - | 1.66 | 1.75 | 4.03 | | 1.96 | | - | - | - | - | - | - |
| 51 | - | 1.72 | 2.07 | - | 1.65 | 1.75 | 4.02 | | 1.96 | | - | - | - | - | - | - |
| 52 | - | 1.71 | 2.08 | - | 1.63 | 1.75 | 3.18 | | 1.97 | | 0.88 | - | - | - | - | - |

| | | | | | | | | | | | | | | | | |
|---|---|---|---|---|---|---|---|---|---|---|---|---|---|---|---|---|
| 53 | - | 1.69 | 2.09 | - | 1.62 | 1.76 | 3.13 | | 1.97 | | 0.95 | - | - | - | - | - |
| 54 | - | 1.67 | 2.11 | - | 3.35 | - | 2.72 | | 1.97 | | 1.01 | 0.38 | - | - | - | - |
| 55 | - | 1.65 | 2.13 | - | 3.35 | - | 2.63 | | 1.97 | | 1.07 | 0.44 | - | - | - | - |
| 56 | - | 1.63 | 2.14 | - | 3.35 | - | 2.55 | | 1.97 | | 1.13 | 0.49 | - | - | - | - |
| 57 | - | 1.61 | 2.16 | - | 3.35 | - | 2.48 | | 1.98 | | 1.18 | 0.54 | - | - | - | - |
| 58 | - | 1.57 | 2.19 | - | 3.05 | - | 2.41 | | 2.00 | | 1.22 | 0.60 | 1.57 | - | 0.30 | - |
| 59 | - | 1.55 | 2.21 | - | 2.97 | - | 2.36 | | 2.00 | | 1.27 | 0.66 | 1.55 | - | 0.36 | - |
| 60 | 1.13 | 1.51 | 2.25 | - | 2.90 | - | 2.30 | | 2.02 | | 1.30 | - | - | - | - | - |
| 61 | 1.22 | 1.47 | 2.29 | - | 2.84 | - | 2.24 | | 2.04 | | 1.33 | - | - | - | - | - |
| 62 | 1.31 | 1.42 | 2.36 | - | 2.49 | - | 2.19 | | 2.07 | | 1.36 | - | - | - | - | 0.24 |
| 63 | 1.39 | 1.36 | 2.41 | - | 2.42 | - | 2.14 | | 2.10 | | 1.37 | - | - | - | - | 0.25 |
| 64 | 1.46 | 1.28 | 2.51 | - | 2.35 | - | 2.08 | | 2.15 | | 1.37 | - | - | - | - | 0.22 |
| 65 | 1.51 | 1.20 | 2.84 | - | 2.27 | - | 2.05 | | 2.20 | | 1.36 | - | - | - | - | - |
| 66 | 1.56 | 1.10 | 2.91 | - | 2.23 | - | 2.02 | | 2.25 | | 1.34 | - | - | - | - | - |
| 67 | 1.59 | - | 2.98 | - | 2.20 | - | 2.00 | | 2.31 | | 1.31 | - | 0.64 | 0.34 | - | - |
| 68 | 1.62 | - | 3.07 | - | 2.18 | - | 1.99 | | 2.38 | | 1.26 | - | 0.58 | 0.27 | - | - |
| 69 | 1.65 | - | 3.34 | - | 2.15 | - | 1.99 | | 2.44 | | 1.22 | - | 0.52 | - | - | - |
| 70 | 1.67 | - | 3.34 | - | 2.13 | - | 1.98 | | 2.51 | | 1.17 | - | 0.46 | - | - | - |
| 71 | 1.69 | - | 3.34 | - | 2.11 | - | 1.97 | | 2.58 | | 1.12 | - | 0.40 | - | - | - |
| 72 | 1.71 | - | 3.35 | - | 2.10 | - | 1.97 | | 2.67 | | 1.07 | - | 0.33 | - | - | - |
| 73 | 1.73 | - | 1.60 | 1.78 | 2.08 | - | 1.97 | | 3.01 | | 1.03 | - | - | - | - | - |
| 74 | 1.74 | - | 1.62 | 1.76 | 2.07 | - | 1.97 | | 3.04 | | 0.97 | - | - | - | - | - |
| 75 | 1.75 | - | 1.64 | 1.76 | 2.06 | - | 1.96 | | 3.06 | | 0.92 | - | - | - | - | - |
| 76 | 1.77 | - | 1.65 | 1.77 | 2.05 | - | 1.97 | | 3.10 | | 0.87 | - | - | - | - | - |
| 77 | 1.78 | - | 1.66 | 1.76 | 2.04 | - | 1.96 | | 3.15 | | 0.82 | - | - | - | - | - |
| 78 | 1.79 | - | 1.67 | 1.77 | 2.03 | - | 1.97 | | 3.93 | | - | - | - | - | - | - |
| 79 | 1.80 | - | 1.67 | 1.77 | 2.03 | - | 1.97 | | 3.94 | | - | - | - | - | - | - |
| 80 | 1.81 | - | 1.68 | 1.76 | 2.02 | - | 1.97 | | 3.93 | | - | - | - | - | - | - |
| 81 | 1.81 | - | 1.69 | 1.77 | 2.02 | - | 1.97 | | 3.93 | | - | - | - | - | - | - |
| 82 | 1.81 | - | 1.69 | 1.77 | 2.01 | - | 1.97 | | 3.93 | | - | - | - | - | - | - |
| 83 | 1.82 | - | 1.69 | 1.77 | 2.01 | - | 1.97 | | 3.94 | | - | - | - | - | - | - |
| 84 | 1.82 | - | 1.70 | 1.76 | 2.01 | - | 1.97 | | 3.93 | | - | - | - | - | - | - |
| 85 | 1.83 | - | 1.70 | 1.76 | 2.01 | - | 1.97 | | 3.93 | | - | - | - | - | - | - |
| 86 | 1.83 | - | 1.71 | 1.77 | 2.01 | - | 1.97 | | 3.93 | | - | - | - | - | - | - |

**Table S5.** Electronic population of basins for the rearrangement of nitrones **5**

| IRC | V(C1,C6) | V(C3,C4) | V1(C4,C5) | V2(C4C5) | V1C5,C6) | V2(C5,C6) | V1(C1,N2) | V2(C1,N2) | V1(C3,N2) | V2(C3,N2) | V(N2) | V(C1) | V(C3) | V(C4) | V(C5) | V(C6) |
|---|---|---|---|---|---|---|---|---|---|---|---|---|---|---|---|---|
| 36 | - | 1.81 | 2.02 | | 3.46 | | 4.00 | | 2.03 | | - | - | - | - | - | - |
| 37 | - | 1.81 | 2.02 | | 3.46 | | 4.00 | | 2.03 | | - | - | - | - | - | - |
| 38 | - | 1.81 | 2.02 | | 3.45 | | 4.00 | | 2.03 | | - | - | - | - | - | - |
| 39 | - | 1.81 | 2.02 | | 3.45 | | 3.99 | | 2.03 | | - | - | - | - | - | - |
| 40 | - | 1.80 | 2.02 | | 3.45 | | 3.99 | | 2.03 | | - | - | - | - | - | - |
| 41 | - | 1.80 | 2.02 | | 3.45 | | 3.99 | | 2.03 | | - | - | - | - | - | - |
| 42 | - | 1.80 | 2.02 | | 3.44 | | 3.99 | | 2.03 | | - | - | - | - | - | - |
| 43 | - | 1.79 | 2.02 | | 3.44 | | 4.00 | | 2.02 | | - | - | - | - | - | - |
| 44 | - | 1.78 | 2.02 | | 3.44 | | 4.00 | | 2.02 | | - | - | - | - | - | - |
| 45 | - | 1.78 | 2.03 | | 3.42 | | 4.00 | | 2.02 | | - | - | - | - | - | - |

| | | | | | | | | | | | | |
|---|---|---|---|---|---|---|---|---|---|---|---|---|
| 46 | - | 1.77 | 2.03 | 3.42 | 4.00 | 2.02 | - | - | - | - | - | - |
| 47 | - | 1.77 | 2.03 | 3.41 | 4.01 | 2.02 | - | - | - | - | - | - |
| 48 | - | 1.76 | 2.04 | 3.40 | 4.01 | 2.01 | - | - | - | - | - | - |
| 49 | - | 1.75 | 2.05 | 3.40 | 4.03 | 2.01 | - | - | - | - | - | - |
| 50 | - | 1.74 | 2.05 | 3.38 | 4.04 | 2.00 | - | - | - | - | - | - |
| 51 | - | 1.73 | 2.06 | 3.38 | 3.63 | 2.00 | - | 0.42 | - | - | - | - |
| 52 | - | 1.72 | 2.07 | 3.36 | 3.63 | 2.00 | - | 0.44 | - | - | - | - |
| 53 | - | 1.70 | 2.08 | 3.35 | 2.85 | 2.00 | 0.77 | 0.48 | - | - | - | - |
| 54 | - | 1.69 | 2.10 | 3.33 | 2.78 | 1.99 | 0.84 | 0.51 | - | - | - | - |
| 55 | - | 1.67 | 2.11 | 3.31 | 2.69 | 1.99 | 0.91 | 0.55 | - | - | - | - |
| 56 | - | 1.64 | 2.13 | 3.28 | 2.61 | 1.99 | 0.98 | 0.60 | - | - | - | - |
| 57 | - | 1.62 | 2.15 | 3.29 | 2.54 | 2.00 | 1.05 | 0.64 | - | - | - | - |
| 58 | - | 1.59 | 2.17 | 3.28 | 2.48 | 2.00 | 1.11 | 0.69 | 1.59 | - | - | - |
| 59 | - | 1.57 | 2.20 | 3.02 | 2.41 | 2.00 | 1.18 | 0.74 | 1.57 | - | 0.23 | - |
| 60 | 1.08 | 1.53 | 2.23 | 2.94 | 2.35 | 2.01 | 1.23 | - | - | - | - | - |
| 61 | 1.18 | 1.50 | 2.26 | 2.87 | 2.29 | 2.02 | 1.27 | - | - | - | - | - |
| 62 | 1.28 | 1.46 | 2.30 | 2.50 | 2.24 | 2.05 | 1.31 | - | - | - | - | 0.30 |
| 63 | 1.36 | 1.41 | 2.35 | 2.42 | 2.18 | 2.07 | 1.35 | - | - | - | - | 0.32 |
| 64 | 1.43 | 1.35 | 2.40 | 2.35 | 2.11 | 2.14 | 1.37 | - | - | - | - | 0.32 |
| 65 | 1.50 | 1.28 | 2.78 | 2.29 | 2.06 | 2.17 | 1.38 | - | - | - | - | - |
| 66 | 1.55 | 1.19 | 2.84 | 2.25 | 2.03 | 2.21 | 1.38 | - | - | - | - | - |
| 67 | 1.59 | 1.07 | 2.92 | 2.22 | 2.01 | 2.26 | 1.37 | - | - | - | - | - |
| 68 | 1.62 | - | 2.98 | 2.18 | 1.99 | 2.32 | 1.34 | - | 0.63 | 0.32 | - | - |
| 69 | 1.64 | - | 3.07 | 2.16 | 1.98 | 2.38 | 1.30 | - | 0.57 | 0.25 | - | - |
| 70 | 1.67 | - | 3.33 | 2.13 | 1.97 | 2.44 | 1.25 | - | 0.50 | - | - | - |
| 71 | 1.69 | - | 3.34 | 2.11 | 1.96 | 2.52 | 1.21 | - | 0.43 | - | - | - |
| 72 | 1.71 | - | 3.33 | 2.09 | 1.95 | 2.60 | 1.16 | - | 0.36 | - | - | - |
| 73 | 1.73 | - | 3.36 | 2.08 | 1.95 | 2.98 | 1.10 | - | - | - | - | - |
| 74 | 1.74 | - | 3.38 | 2.06 | 1.94 | 3.00 | 1.05 | - | - | - | - | - |
| 75 | 1.76 | - | 3.39 | 2.05 | 1.94 | 3.03 | 1.00 | - | - | - | - | - |
| 76 | 1.77 | - | 3.40 | 2.04 | 1.94 | 3.06 | 0.95 | - | - | - | - | - |
| 77 | 1.78 | - | 3.41 | 2.03 | 1.94 | 3.10 | 0.89 | - | - | - | - | - |
| 78 | 1.79 | - | 3.42 | 2.03 | 1.94 | 3.13 | 0.85 | - | - | - | - | - |
| 79 | 1.80 | - | 3.43 | 2.02 | 1.95 | 3.95 | - | - | - | - | - | - |
| 80 | 1.80 | - | 3.44 | 2.02 | 1.95 | 3.96 | - | - | - | - | - | - |
| 81 | 1.81 | - | 3.45 | 2.01 | 1.95 | 3.96 | - | - | - | - | - | - |
| 82 | 1.82 | - | 3.45 | 2.01 | 1.95 | 3.96 | - | - | - | - | - | - |
| 83 | 1.82 | - | 3.46 | 2.00 | 1.95 | 3.97 | - | - | - | - | - | - |
| 84 | 1.83 | - | 3.46 | 2.00 | 1.95 | 3.97 | - | - | - | - | - | - |
| 85 | 1.83 | - | 3.47 | 2.00 | 1.95 | 3.97 | - | - | - | - | - | - |
| 86 | 1.83 | - | 3.47 | 2.00 | 1.95 | 3.96 | - | - | - | - | - | - |

**Table S6.** Electronic population of basins for the rearrangement of nitrones **6**

| IRC | V(C1,C6) | V(C3,C4) | V1(C4,C5) | V2(C4C5) | V1C5,C6) | V2(C5,C6) | V1(C1,N2) | V2(C1,N2) | V1(C3,N2) | V2(C3,N2) | V(N2) | V(C1) | V(C3) | V(C4) | V(C5) | V(C6) |
|---|---|---|---|---|---|---|---|---|---|---|---|---|---|---|---|---|
| 36 | - | 1.81 | 2.01 | - | 1.72 | 1.76 | 3.97 | | 1.99 | | - | - | - | - | - | - |
| 37 | - | 1.81 | 2.01 | - | 1.72 | 1.77 | 3.98 | | 1.99 | | - | - | - | - | - | - |
| 38 | - | 1.81 | 2.01 | - | 1.72 | 1.77 | 3.98 | | 1.99 | | - | - | - | - | - | - |
| 39 | - | 1.81 | 2.01 | - | 1.71 | 1.77 | 3.98 | | 1.99 | | - | - | - | - | - | - |
| 40 | - | 1.80 | 2.01 | - | 1.76 | 1.70 | 3.97 | | 1.99 | | - | - | - | - | - | - |
| 41 | - | 1.80 | 2.02 | - | 1.76 | 1.70 | 3.98 | | 1.99 | | - | - | - | - | - | - |
| 42 | - | 1.80 | 2.01 | - | 1.76 | 1.70 | 3.98 | | 1.98 | | - | - | - | - | - | - |
| 43 | - | 1.79 | 2.02 | - | 1.76 | 1.70 | 3.98 | | 1.98 | | - | - | - | - | - | - |
| 44 | - | 1.79 | 2.02 | - | 1.76 | 1.69 | 3.98 | | 1.98 | | - | - | - | - | - | - |
| 45 | - | 1.78 | 2.03 | - | 1.76 | 1.69 | 3.99 | | 1.97 | | - | - | - | - | - | - |
| 46 | - | 1.78 | 2.03 | - | 1.76 | 1.68 | 3.99 | | 1.97 | | - | - | - | - | - | - |
| 47 | - | 1.77 | 2.03 | - | 1.76 | 1.68 | 4.00 | | 1.97 | | - | - | - | - | - | - |
| 48 | - | 1.76 | 2.04 | - | 1.75 | 1.67 | 4.00 | | 1.96 | | - | - | - | - | - | - |
| 49 | - | 1.75 | 2.05 | - | 1.75 | 1.66 | 4.01 | | 1.96 | | - | - | - | - | - | - |
| 50 | - | 1.74 | 2.05 | - | 1.75 | 1.66 | 4.02 | | 1.96 | | - | - | - | - | - | - |
| 51 | - | 1.72 | 2.06 | - | 1.65 | 1.75 | 4.03 | | 1.97 | | - | - | - | - | - | - |
| 52 | - | 1.71 | 2.08 | - | 1.64 | 1.75 | 3.21 | | 1.96 | | 0.85 | - | - | - | - | - |
| 53 | - | 1.70 | 2.09 | - | 1.75 | 1.62 | 3.17 | | 1.96 | | 0.91 | - | - | - | - | - |
| 54 | - | 1.68 | 2.10 | - | 1.65 | 1.72 | 2.78 | | 1.97 | | 0.97 | 0.36 | - | - | - | - |
| 55 | - | 1.66 | 2.12 | - | 3.33 | - | 2.68 | | 1.97 | | 1.03 | 0.43 | - | - | - | - |
| 56 | - | 1.64 | 2.14 | - | 3.34 | - | 2.61 | | 1.97 | | 1.09 | 0.48 | - | - | - | - |
| 57 | - | 1.61 | 2.16 | - | 3.33 | - | 2.53 | | 1.98 | | 1.15 | 0.54 | - | - | - | - |
| 58 | - | 1.58 | 2.19 | - | 3.07 | - | 2.45 | | 1.99 | | 1.20 | 0.60 | - | - | 0.25 | - |
| 59 | - | 1.55 | 2.21 | - | 2.99 | - | 2.39 | | 2.00 | | 1.24 | 0.66 | - | - | 0.32 | - |
| 60 | 1.09 | 1.51 | 2.24 | - | 2.92 | - | 2.33 | | 2.02 | | 1.28 | - | - | - | - | - |
| 61 | 1.19 | 1.48 | 2.27 | - | 2.85 | - | 2.27 | | 2.03 | | 1.32 | - | - | - | - | - |
| 62 | 1.29 | 1.43 | 2.32 | - | 2.47 | - | 2.22 | | 2.06 | | 1.34 | - | - | - | - | 0.32 |
| 63 | 1.37 | 1.37 | 2.37 | - | 2.39 | - | 2.16 | | 2.10 | | 1.36 | - | - | - | - | 0.33 |
| 64 | 1.44 | 1.31 | 2.44 | - | 2.33 | - | 2.10 | | 2.15 | | 1.37 | - | - | - | - | 0.32 |
| 65 | 1.50 | 1.22 | 2.82 | - | 2.28 | - | 2.06 | | 2.20 | | 1.36 | - | - | - | - | - |
| 66 | 1.54 | 1.12 | 2.88 | - | 2.24 | - | 2.04 | | 2.26 | | 1.34 | - | - | - | - | - |
| 67 | 1.58 | 1.00 | 2.96 | - | 2.21 | - | 2.02 | | 2.32 | | 1.31 | - | - | - | - | - |
| 68 | 1.61 | - | 3.04 | - | 2.18 | - | 2.01 | | 2.37 | | 1.27 | - | 0.59 | 0.28 | - | - |
| 69 | 1.64 | - | 3.32 | - | 2.16 | - | 2.00 | | 2.44 | | 1.22 | - | 0.53 | - | - | - |
| 70 | 1.66 | - | 3.33 | - | 2.14 | - | 1.99 | | 2.51 | | 1.18 | - | 0.47 | - | - | - |
| 71 | 1.68 | - | 3.33 | - | 2.12 | - | 1.98 | | 2.59 | | 1.13 | - | 0.40 | - | - | - |
| 72 | 1.70 | - | 3.34 | - | 2.10 | - | 1.97 | | 2.67 | | 1.09 | - | 0.33 | - | - | - |
| 73 | 1.72 | - | 1.78 | 1.58 | 2.08 | - | 1.97 | | 3.02 | | 1.03 | - | - | - | - | - |
| 74 | 1.74 | - | 1.77 | 1.61 | 2.07 | - | 1.97 | | 3.04 | | 0.98 | - | - | - | - | - |
| 75 | 1.75 | - | 1.76 | 1.63 | 2.06 | - | 1.97 | | 3.06 | | 0.93 | - | - | - | - | - |
| 76 | 1.76 | - | 1.76 | 1.64 | 2.05 | - | 1.97 | | 3.11 | | 0.87 | - | - | - | - | - |
| 77 | 1.78 | - | 1.76 | 1.65 | 2.04 | - | 1.97 | | 3.14 | | 0.82 | - | - | - | - | - |
| 78 | 1.79 | - | 1.66 | 1.76 | 2.04 | - | 1.98 | | 3.92 | | - | - | - | - | - | - |
| 79 | 1.79 | - | 1.67 | 1.76 | 2.03 | - | 1.98 | | 3.94 | | - | - | - | - | - | - |

| | | | | | | | | | | | | | | | | |
|---|---|---|---|---|---|---|---|---|---|---|---|---|---|---|---|---|
| 80 | 1.80 | - | 1.76 | 1.67 | 2.03 | - | 1.97 | | 3.94 | | - | - | - | - | - | - |
| 81 | 1.81 | - | 1.76 | 1.68 | 2.02 | - | 1.97 | | 3.94 | | - | - | - | - | - | - |
| 82 | 1.81 | - | 1.76 | 1.68 | 2.02 | - | 1.97 | | 3.94 | | - | - | - | - | - | - |
| 83 | 1.82 | - | 1.77 | 1.69 | 2.01 | - | 1.97 | | 3.93 | | - | - | - | - | - | - |
| 84 | 1.82 | - | 1.77 | 1.70 | 2.01 | - | 1.97 | | 3.94 | | - | - | - | - | - | - |
| 85 | 1.82 | - | 1.76 | 1.70 | 2.01 | - | 1.98 | | 3.93 | | - | - | - | - | - | - |
| 86 | 1.83 | - | 1.76 | 1.70 | 2.01 | - | 1.98 | | 3.93 | | - | - | - | - | - | - |

**Table S7.** Electronic population of basins for the rearrangement of nitrones (*Z*)-**7**

| IRC | V(C1,C6) | V(C3,C4) | V1(C4,C5) | V2(C4C5) | V1(C5,C6) | V2(C5,C6) | V1(C1,N2) | V2(C1,N2) | V1(C3,N2) | V2(C3,N2) | V(N2) | V(C1) | V(C3) | V(C4) | V(C5) | V(C6) |
|---|---|---|---|---|---|---|---|---|---|---|---|---|---|---|---|---|
| 36 | - | 1.81 | 2.01 | - | 1.81 | 1.76 | 3.95 | - | 1.97 | - | - | - | - | - | - | - |
| 37 | - | 1.81 | 2.01 | - | 1.81 | 1.76 | 3.95 | - | 1.98 | - | - | - | - | - | - | - |
| 38 | - | 1.81 | 2.01 | - | 1.81 | 1.75 | 3.95 | - | 1.98 | - | - | - | - | - | - | - |
| 39 | - | 1.80 | 2.01 | - | 1.81 | 1.74 | 3.95 | - | 1.98 | - | - | - | - | - | - | - |
| 40 | - | 1.80 | 2.01 | - | 1.81 | 1.73 | 3.96 | - | 1.97 | - | - | - | - | - | - | - |
| 41 | - | 1.79 | 2.01 | - | 1.81 | 1.73 | 3.96 | - | 1.97 | - | - | - | - | - | - | - |
| 42 | - | 1.79 | 2.01 | - | 1.82 | 1.72 | 3.95 | - | 1.98 | - | - | - | - | - | - | - |
| 43 | - | 1.79 | 2.01 | - | 1.81 | 1.72 | 3.95 | - | 1.97 | - | - | - | - | - | - | - |
| 44 | - | 1.78 | 2.01 | - | 1.82 | 1.73 | 3.95 | - | 1.98 | - | - | - | - | - | - | - |
| 45 | - | 1.77 | 2.01 | - | 1.82 | 1.72 | 3.96 | - | 1.98 | - | - | - | - | - | - | - |
| 46 | - | 1.76 | 2.02 | - | 1.82 | 1.70 | 3.95 | - | 1.98 | - | - | - | - | - | - | - |
| 47 | - | 1.75 | 2.03 | - | 1.82 | 1.70 | 3.95 | - | 1.98 | - | - | - | - | - | - | - |
| 48 | - | 1.73 | 2.04 | - | 1.83 | 1.68 | 3.95 | - | 1.99 | - | - | - | - | - | - | - |
| 49 | - | 1.69 | 2.05 | - | 1.83 | 1.66 | 3.95 | - | 1.99 | - | - | - | - | - | - | - |
| 50 | - | 1.65 | 2.08 | - | 1.82 | 1.64 | 3.95 | - | 2.01 | - | - | - | - | - | - | - |
| 51 | - | 1.61 | 2.10 | - | 1.82 | 1.63 | 3.95 | - | 2.02 | - | - | - | - | - | - | - |
| 52 | - | 1.55 | 2.14 | - | 1.82 | 1.60 | 3.69 | - | 2.04 | - | - | 0.26 | - | - | - | - |
| 53 | - | 1.50 | 2.17 | - | 1.82 | 1.59 | 3.65 | - | 2.06 | - | - | 0.30 | - | - | - | - |
| 54 | - | 1.44 | 2.22 | - | 3.35 | - | 3.60 | - | 2.09 | - | - | 0.36 | - | - | - | - |
| 55 | - | 1.37 | 2.27 | - | 3.32 | - | 3.55 | - | 2.11 | - | - | 0.41 | - | - | - | - |
| 56 | - | 1.29 | 2.32 | - | 3.30 | - | 3.49 | - | 2.15 | - | - | 0.46 | - | - | - | - |
| 57 | - | 1.20 | 2.39 | - | 3.25 | - | 3.42 | - | 2.20 | - | - | 0.51 | - | - | - | - |
| 58 | - | - | 2.47 | - | 3.20 | - | 2.76 | - | 2.29 | - | 0.53 | 0.56 | 0.73 | 0.38 | - | - |
| 59 | - | - | 2.59 | - | 3.15 | - | 2.61 | - | 2.31 | - | 0.70 | 0.61 | 0.67 | 0.33 | - | - |
| 60 | - | - | 2.78 | - | 3.03 | - | 2.48 | - | 2.43 | - | 0.71 | 0.66 | 0.61 | 0.26 | - | - |
| 61 | - | - | 3.15 | - | 2.57 | - | 2.36 | - | 2.56 | - | 0.70 | 0.71 | 0.56 | - | 0.35 | - |
| 62 | - | - | 3.20 | - | 2.47 | - | 2.29 | - | 2.64 | - | 0.70 | 0.75 | 0.50 | - | 0.40 | - |
| 63 | - | - | 3.23 | - | 2.40 | - | 2.23 | - | 2.72 | - | 0.68 | 0.81 | 0.44 | - | 0.44 | - |
| 64 | - | - | 3.25 | - | 2.35 | - | 2.17 | - | 2.80 | - | 0.67 | 0.85 | 0.38 | - | 0.47 | - |
| 65 | 1.40 | - | 3.28 | - | 2.30 | - | 2.13 | - | 2.87 | - | 0.67 | - | 0.33 | - | - | - |
| 66 | 1.46 | - | 1.75 | 1.56 | 2.25 | - | 2.10 | - | 3.59 | - | - | - | 0.28 | - | - | - |
| 67 | 1.52 | - | 1.78 | 1.55 | 2.22 | - | 2.07 | - | 3.88 | - | - | - | - | - | - | - |
| 68 | 1.56 | - | 1.77 | 1.59 | 2.19 | - | 2.05 | - | 3.89 | - | - | - | - | - | - | - |
| 69 | 1.61 | - | 1.77 | 1.61 | 2.16 | - | 2.03 | - | 3.90 | - | - | - | - | - | - | - |
| 70 | 1.65 | - | 1.77 | 1.63 | 2.13 | - | 2.01 | - | 3.90 | - | - | - | - | - | - | - |

| | | | | | | | | | | | | | | | | |
|---|---|---|---|---|---|---|---|---|---|---|---|---|---|---|---|---|
| 71 | 1.69 | - | 1.77 | 1.64 | 2.11 | - | 2.00 | - | 3.91 | - | - | - | - | - | - | - |
| 72 | 1.73 | - | 1.77 | 1.65 | 2.09 | - | 1.99 | - | 3.92 | - | - | - | - | - | - | - |
| 73 | 1.75 | - | 1.77 | 1.66 | 2.08 | - | 1.99 | - | 3.93 | - | - | - | - | - | - | - |
| 74 | 1.78 | - | 1.77 | 1.67 | 2.07 | - | 1.98 | - | 3.93 | - | - | - | - | - | - | - |
| 75 | 1.80 | - | 1.76 | 1.68 | 2.05 | - | 1.97 | - | 3.93 | - | - | - | - | - | - | - |
| 76 | 1.81 | - | 1.68 | 1.77 | 2.04 | - | 1.97 | - | 3.94 | - | - | - | - | - | - | - |
| 77 | 1.83 | - | 1.76 | 1.69 | 2.04 | - | 1.98 | - | 3.94 | - | - | - | - | - | - | - |
| 78 | 1.83 | - | 1.76 | 1.69 | 2.03 | - | 1.98 | - | 3.93 | - | - | - | - | - | - | - |
| 79 | 1.84 | - | 1.76 | 1.70 | 2.03 | - | 1.98 | - | 3.93 | - | - | - | - | - | - | - |
| 80 | 1.85 | - | 1.76 | 1.70 | 2.03 | - | 1.98 | - | 3.93 | - | - | - | - | - | - | - |
| 81 | 1.85 | - | 1.75 | 1.71 | 2.03 | - | 1.98 | - | 3.93 | - | - | - | - | - | - | - |
| 82 | 1.86 | - | 1.76 | 1.71 | 2.03 | - | 1.99 | - | 3.93 | - | - | - | - | - | - | - |
| 83 | 1.86 | - | 1.75 | 1.71 | 2.03 | - | 1.98 | - | 3.94 | - | - | - | - | - | - | - |
| 84 | 1.86 | - | 1.75 | 1.72 | 2.03 | - | 1.98 | - | 3.94 | - | - | - | - | - | - | - |
| 85 | 1.86 | - | 1.76 | 1.72 | 2.02 | - | 1.98 | - | 3.94 | - | - | - | - | - | - | - |
| 86 | 1.87 | - | 1.76 | 1.72 | 2.02 | - | 1.98 | - | 3.94 | - | - | - | - | - | - | - |

**Table S8.** Electronic population of basins for the rearrangement of nitrones (*E*)-**7**

| IRC | V(C1,C6) | V(C3,C4) | V1(C4,C5) | V2(C4C5) | V1C5,C6) | V2(C5,C6) | V1(C1,N2) | V2(C1,N2) | V1(C3,N2) | V2(C3,N2) | V(N2) | V(C1) | V(C3) | V(C4) | V(C5) | V(C6) |
|---|---|---|---|---|---|---|---|---|---|---|---|---|---|---|---|---|
| 36 | - | 1.81 | 2.00 | | 3.54 | | 3.91 | - | 1.98 | - | - | - | - | - | - | - |
| 37 | - | 1.80 | 2.01 | | 3.54 | | 3.92 | - | 1.97 | - | - | - | - | - | - | - |
| 38 | - | 1.80 | 2.01 | | 3.53 | | 3.92 | - | 1.98 | - | - | - | - | - | - | - |
| 39 | - | 1.79 | 2.01 | | 3.53 | | 3.92 | - | 1.97 | - | - | - | - | - | - | - |
| 40 | - | 1.79 | 2.01 | | 3.52 | | 3.93 | - | 1.97 | - | - | - | - | - | - | - |
| 41 | - | 1.79 | 2.01 | | 3.53 | | 3.92 | - | 1.97 | - | - | - | - | - | - | - |
| 42 | - | 1.79 | 2.01 | | 3.52 | | 3.92 | - | 1.97 | - | - | - | - | - | - | - |
| 43 | - | 1.79 | 2.01 | | 3.51 | | 3.93 | - | 1.97 | - | - | - | - | - | - | - |
| 44 | - | 1.78 | 2.02 | | 3.51 | | 3.93 | - | 1.97 | - | - | - | - | - | - | - |
| 45 | - | 1.77 | 2.02 | | 3.50 | | 3.93 | - | 1.97 | - | - | - | - | - | - | - |
| 46 | - | 1.77 | 2.03 | | 3.49 | | 3.94 | - | 1.97 | - | - | - | - | - | - | - |
| 47 | - | 1.76 | 2.03 | | 3.48 | | 3.94 | - | 1.97 | - | - | - | - | - | - | - |
| 48 | - | 1.75 | 2.04 | | 3.48 | | 3.95 | - | 1.97 | - | - | - | - | - | - | - |
| 49 | - | 1.74 | 2.05 | | 3.46 | | 3.13 | - | 1.97 | - | 0.84 | - | - | - | - | - |
| 50 | - | 1.72 | 2.06 | | 3.45 | | 3.10 | - | 1.97 | - | 0.89 | - | - | - | - | - |
| 51 | - | 1.70 | 2.07 | | 3.43 | | 3.06 | - | 1.97 | - | 0.94 | - | - | - | - | - |
| 52 | - | 1.68 | 2.09 | | 3.42 | | 3.04 | - | 1.98 | - | 0.99 | - | - | - | - | - |
| 53 | - | 1.65 | 2.12 | | 3.38 | | 2.66 | - | 1.98 | - | 1.05 | 0.36 | - | - | - | - |
| 54 | - | 1.61 | 2.14 | | 3.37 | | 2.58 | - | 1.99 | - | 1.09 | 0.43 | - | - | - | - |
| 55 | - | 1.56 | 2.18 | | 3.36 | | 2.51 | - | 2.01 | - | 1.14 | 0.50 | - | - | - | - |
| 56 | - | 1.51 | 2.21 | | 3.35 | | 2.44 | - | 2.03 | - | 1.17 | 0.56 | - | - | - | - |
| 57 | - | 1.45 | 2.26 | | 3.04 | | 2.38 | - | 2.05 | - | 1.21 | 0.62 | - | - | 0.29 | - |
| 58 | - | 1.38 | 2.32 | | 2.93 | | 2.32 | - | 2.09 | - | 1.22 | 0.69 | - | - | 0.36 | - |
| 59 | 1.16 | 1.30 | 2.38 | | 2.85 | | 2.27 | - | 2.13 | - | 1.24 | - | - | - | pop. | - |
| 60 | 1.26 | 1.20 | 2.47 | | 2.76 | | 2.21 | - | 2.18 | - | 1.24 | - | - | - | pop. | - |
| 61 | 1.34 | 1.10 | 2.80 | | 2.44 | | 2.15 | - | 2.26 | - | 1.23 | - | - | - | pop. | - |
| 62 | 1.42 | - | 2.89 | | 2.37 | | 2.09 | - | 2.32 | - | 1.22 | - | 0.64 | 0.34 | pop. | - |
| 63 | 1.48 | - | 2.99 | | 2.31 | | 2.05 | - | 2.39 | - | 1.20 | - | 0.58 | 0.28 | pop. | - |

| | | | | | | | | | | | | | | |
|---|---|---|---|---|---|---|---|---|---|---|---|---|---|---|
| 64 | 1.54 | - | 3.29 | 2.26 | 2.02 | - | 2.45 | - | 1.18 | - | 0.51 | - | pop. | - |
| 65 | 1.59 | - | 3.31 | 2.22 | 2.00 | - | 2.52 | - | 1.14 | - | 0.44 | - | pop. | - |
| 66 | 1.63 | - | 3.31 | 2.19 | 1.98 | - | 2.60 | - | 1.10 | - | 0.38 | - | pop. | - |
| 67 | 1.66 | - | 3.35 | 2.16 | 1.97 | - | 2.69 | - | 1.05 | - | 0.29 | - | pop. | - |
| 68 | 1.69 | - | 3.36 | 2.13 | 1.96 | - | 3.00 | - | 1.00 | - | - | - | pop. | - |
| 69 | 1.72 | - | 3.38 | 2.11 | 1.95 | - | 3.03 | - | 0.95 | - | - | - | pop. | - |
| 70 | 1.74 | - | 3.40 | 2.09 | 1.95 | - | 3.07 | - | 0.90 | - | - | - | pop. | - |
| 71 | 1.76 | - | 3.40 | 2.08 | 1.94 | - | 3.12 | - | 0.84 | - | - | - | pop. | - |
| 72 | 1.78 | - | 3.42 | 2.07 | 1.94 | - | 3.13 | - | 0.81 | - | - | - | - | - |
| 73 | 1.79 | - | 3.44 | 2.06 | 1.95 | - | 3.94 | - | - | - | - | - | - | - |
| 74 | 1.81 | - | 3.44 | 2.05 | 1.95 | - | 3.93 | - | - | - | - | - | - | - |
| 75 | 1.82 | - | 3.45 | 2.05 | 1.95 | - | 3.93 | - | - | - | - | - | - | - |
| 76 | 1.82 | - | 3.46 | 2.04 | 1.95 | - | 3.93 | - | - | - | - | - | - | - |
| 77 | 1.83 | - | 3.46 | 2.04 | 1.95 | - | 3.93 | - | - | - | - | - | - | - |
| 78 | 1.84 | - | 3.47 | 2.04 | 1.95 | - | 3.93 | - | - | - | - | - | - | - |
| 79 | 1.84 | - | 3.47 | 2.03 | 1.95 | - | 3.93 | - | - | - | - | - | - | - |
| 80 | 1.85 | - | 3.47 | 2.03 | 1.95 | - | 3.93 | - | - | - | - | - | - | - |
| 81 | 1.85 | - | 3.47 | 2.03 | 1.95 | - | 3.94 | - | - | - | - | - | - | - |
| 82 | 1.85 | - | 3.47 | 2.03 | 1.95 | - | 3.94 | - | - | - | - | - | - | - |
| 83 | 1.86 | - | 3.48 | 2.03 | 1.95 | - | 3.94 | - | - | - | - | - | - | - |
| 84 | 1.86 | - | 3.48 | 2.03 | 1.96 | - | 3.94 | - | - | - | - | - | - | - |
| 85 | 1.86 | - | 3.48 | 2.02 | 1.96 | - | 3.94 | - | - | - | - | - | - | - |
| 86 | 1.86 | - | 3.49 | 2.02 | 1.96 | - | 3.95 | - | - | - | - | - | - | - |

**Table S9.** Electronic population of basins for the rearrangement of nitrones (*Z*)-**8**

| IRC | V(C1,C6) | V(C3,C4) | V1(C4,C5) | V2(C4C5) | V1C5,C6) | V2(C5,C6) | V1(C1,N2) | V2(C1,N2) | V1(C3,N2) | V2(C3,N2) | V(N2) | V(C1) | V(C3) | V(C4) | V(C5) | V(C6) |
|---|---|---|---|---|---|---|---|---|---|---|---|---|---|---|---|---|
| 36 | - | 1,82 | 2,00 | - | 1,83 | 1,86 | 3,96 | - | 1,97 | | - | - | - | - | - | - |
| 37 | - | 1,81 | 1,99 | - | 1,83 | 1,85 | 3,95 | - | 1,97 | | - | - | - | - | - | - |
| 38 | - | 1,81 | 2,00 | - | 1,82 | 1,85 | 3,96 | - | 1,96 | | - | - | - | - | - | - |
| 39 | - | 1,81 | 2,00 | - | 1,82 | 1,86 | 3,96 | - | 1,95 | | - | - | - | - | - | - |
| 40 | - | 1,80 | 2,01 | - | 1,81 | 1,87 | 3,96 | - | 1,96 | | - | - | - | - | - | - |
| 41 | - | 1,80 | 2,01 | - | 1,81 | 1,87 | 3,96 | - | 1,96 | | - | - | - | - | - | - |
| 42 | - | 1,80 | 2,01 | - | 1,81 | 1,87 | 3,96 | - | 1,96 | | - | - | - | - | - | - |
| 43 | - | 1,80 | 2,01 | - | 1,80 | 1,87 | 3,95 | - | 1,96 | | - | - | - | - | - | - |
| 44 | - | 1,79 | 2,01 | - | 1,80 | 1,87 | 3,95 | - | 1,96 | | - | - | - | - | - | - |
| 45 | - | 1,79 | 2,02 | - | 1,87 | 1,79 | 3,95 | - | 1,97 | | - | - | - | - | - | - |
| 46 | - | 1,78 | 2,02 | - | 1,88 | 1,79 | 3,94 | - | 1,97 | | - | - | - | - | - | - |
| 47 | - | 1,77 | 2,02 | - | 1,88 | 1,78 | 3,95 | - | 1,97 | | - | - | - | - | - | - |
| 48 | - | 1,76 | 2,02 | - | 1,88 | 1,77 | 3,95 | - | 1,97 | | - | - | - | - | - | - |
| 49 | - | 1,75 | 2,02 | - | 1,89 | 1,76 | 3,95 | - | 1,98 | | - | - | - | - | - | - |
| 50 | - | 1,73 | 2,03 | - | 1,90 | 1,76 | 3,96 | - | 1,98 | | - | - | - | - | - | - |
| 51 | - | 1,72 | 2,04 | - | 1,91 | 1,74 | 3,96 | - | 1,98 | | - | - | - | - | - | - |
| 52 | - | 1,70 | 2,05 | - | 1,92 | 1,73 | 3,97 | - | 1,99 | | - | - | - | - | - | - |
| 53 | - | 1,68 | 2,06 | - | 1,70 | 1,95 | 3,64 | - | 1,99 | | - | 0,35 | - | - | - | - |
| 54 | - | 1,65 | 2,08 | - | 3,64 | - | 3,58 | - | 2,01 | | - | 0,41 | - | - | - | - |

| | | | | | | | | | | | | | | | | |
|---|---|---|---|---|---|---|---|---|---|---|---|---|---|---|---|---|
| 55 | - | 1,61 | 2,10 | - | 3,64 | - | 2,76 | - | 2,01 | | 0,80 | 0,48 | - | - | - | - |
| 56 | - | 1,57 | 2,12 | - | 3,64 | - | 2,66 | - | 2,03 | | 0,85 | 0,54 | - | - | - | - |
| 57 | - | 1,52 | 2,15 | - | 3,11 | - | 2,58 | - | 2,05 | | 0,90 | 0,60 | - | - | - | 0,53 |
| 58 | - | 1,46 | 2,19 | - | 2,67 | - | 2,51 | - | 2,08 | | 0,94 | 0,66 | - | - | 0,39 | 0,56 |
| 59 | - | 1,40 | 2,24 | - | 2,55 | - | 2,44 | - | 2,12 | | 0,96 | 0,72 | - | - | 0,48 | 0,58 |
| 60 | - | 1,32 | 2,29 | - | 2,46 | - | 2,36 | - | 2,17 | | 0,98 | 0,77 | - | - | 0,54 | 0,59 |
| 61 | 1,40 | 1,23 | 2,35 | - | 2,39 | - | 2,24 | - | 2,29 | | 0,99 | - | - | - | - | 0,58 |
| 62 | 1,48 | 1,13 | 2,43 | - | 2,33 | - | 2,15 | - | 2,38 | | 0,99 | - | - | - | - | 0,57 |
| 63 | 1,55 | 1,01 | 2,53 | - | 2,29 | - | 2,09 | - | 2,45 | | 0,99 | - | - | - | - | 0,53 |
| 64 | 1,61 | - | 2,67 | - | 2,24 | - | 2,06 | - | 2,51 | | 0,97 | - | 0,63 | 0,24 | - | 0,49 |
| 65 | 1,66 | - | 3,40 | - | 2,21 | - | 2,03 | - | 2,58 | | 0,94 | - | 0,56 | - | - | - |
| 66 | 1,69 | - | 3,40 | - | 2,19 | - | 2,01 | - | 2,65 | | 0,91 | - | 0,50 | - | - | - |
| 67 | 1,73 | - | 3,38 | - | 2,17 | - | 1,99 | - | 2,74 | | 0,87 | - | 0,43 | - | - | - |
| 68 | 1,76 | - | 1,78 | 1,62 | 2,15 | - | 1,98 | - | 2,83 | | 0,82 | - | 0,35 | - | - | - |
| 69 | 1,78 | - | 1,77 | 1,64 | 2,13 | - | 1,98 | - | 3,20 | | 0,78 | - | - | - | - | - |
| 70 | 1,80 | - | 1,77 | 1,65 | 2,12 | - | 1,97 | - | 3,95 | | - | - | - | - | - | - |
| 71 | 1,82 | - | 1,77 | 1,66 | 2,11 | - | 1,96 | - | 3,96 | | - | - | - | - | - | - |
| 72 | 1,83 | - | 1,77 | 1,67 | 2,10 | - | 1,96 | - | 3,95 | | - | - | - | - | - | - |
| 73 | 1,84 | - | 1,76 | 1,68 | 2,10 | - | 1,96 | - | 3,95 | | - | - | - | - | - | - |
| 74 | 1,85 | - | 1,75 | 1,68 | 2,09 | - | 1,96 | - | 3,95 | | - | - | - | - | - | - |
| 75 | 1,86 | - | 1,76 | 1,69 | 2,09 | - | 1,96 | - | 3,95 | | - | - | - | - | - | - |
| 76 | 1,87 | - | 1,75 | 1,69 | 2,08 | - | 1,96 | - | 3,95 | | - | - | - | - | - | - |
| 77 | 1,87 | - | 1,70 | 1,76 | 2,08 | - | 1,96 | - | 3,95 | | - | - | - | - | - | - |
| 78 | 1,88 | - | 1,75 | 1,70 | 2,08 | - | 1,95 | - | 3,96 | | - | - | - | - | - | - |
| 79 | 1,88 | - | 1,71 | 1,75 | 2,08 | - | 1,95 | - | 3,96 | | - | - | - | - | - | - |
| 80 | 1,89 | - | 1,71 | 1,75 | 2,07 | - | 1,96 | - | 3,96 | | - | - | - | - | - | - |
| 81 | 1,89 | - | 1,74 | 1,72 | 2,07 | - | 1,96 | - | 3,96 | | - | - | - | - | - | - |
| 82 | 1,89 | - | 1,72 | 1,74 | 2,07 | - | 1,96 | - | 3,97 | | - | - | - | - | - | - |
| 83 | 1,89 | - | 1,72 | 1,74 | 2,07 | - | 1,96 | - | 3,96 | | - | - | - | - | - | - |
| 84 | 1,90 | - | 1,72 | 1,74 | 2,07 | - | 1,96 | - | 3,97 | | - | - | - | - | - | - |
| 85 | 1,90 | - | 1,73 | 1,74 | 2,07 | - | 1,96 | - | 3,96 | | - | - | - | - | - | - |
| 86 | 1,90 | - | 1,74 | 1,73 | 2,07 | - | 1,97 | - | 3,97 | | - | - | - | - | - | - |

**Table S10.** Electronic population of basins for the rearrangement of nitrones (*E*)-**8**

| IRC | V(C1,C6) | V(C3,C4) | V1(C4,C5) | V2(C4C5) | V1C5,C6) | V2(C5,C6) | V1(C1,N2) | V2(C1,N2) | V1(C3,N2) | V2(C3,N2) | V(N2) | V(C1) | V(C3) | V(C4) | V(C5) | V(C6) |
|---|---|---|---|---|---|---|---|---|---|---|---|---|---|---|---|---|
| 36 | - | 1,81 | 1,98 | - | 1,82 | 1,88 | 3,93 | - | 1,98 | - | - | - | - | - | - | - |
| 37 | - | 1,81 | 1,98 | - | 1,82 | 1,88 | 3,94 | - | 1,98 | - | - | - | - | - | - | - |
| 38 | - | 1,80 | 1,98 | - | 1,83 | 1,87 | 3,93 | - | 1,98 | - | - | - | - | - | - | - |
| 39 | - | 1,80 | 1,99 | - | 1,82 | 1,87 | 3,93 | - | 1,97 | - | - | - | - | - | - | - |
| 40 | - | 1,80 | 2,00 | - | 1,82 | 1,87 | 3,93 | - | 1,97 | - | - | - | - | - | - | - |
| 41 | - | 1,80 | 1,99 | - | 1,82 | 1,87 | 3,93 | - | 1,97 | - | - | - | - | - | - | - |
| 42 | - | 1,79 | 1,99 | - | 1,82 | 1,87 | 3,93 | - | 1,97 | - | - | - | - | - | - | - |
| 43 | - | 1,79 | 1,99 | - | 1,81 | 1,87 | 3,92 | - | 1,98 | - | - | - | - | - | - | - |
| 44 | - | 1,79 | 1,99 | - | 1,80 | 1,88 | 3,93 | - | 1,97 | - | - | - | - | - | - | - |
| 45 | - | 1,78 | 1,99 | - | 1,81 | 1,87 | 3,93 | - | 1,97 | - | - | - | - | - | - | - |
| 46 | - | 1,78 | 2,00 | - | 1,81 | 1,86 | 3,93 | - | 1,97 | - | - | - | - | - | - | - |
| 47 | - | 1,77 | 2,01 | - | 1,81 | 1,86 | 3,93 | - | 1,97 | - | - | - | - | - | - | - |

| | | | | | | | | | | | | | | | | |
|---|---|---|---|---|---|---|---|---|---|---|---|---|---|---|---|---|
| 48 | - | 1,76 | 2,01 | - | 1,80 | 1,86 | 3,93 | - | 1,97 | - | - | - | - | - | - | - |
| 49 | - | 1,75 | 2,02 | - | 1,79 | 1,85 | 3,92 | - | 1,97 | - | - | - | - | - | - | - |
| 50 | - | 1,73 | 2,04 | - | 1,84 | 1,78 | 3,12 | - | 1,97 | - | 0,84 | - | - | - | - | - |
| 51 | - | 1,71 | 2,05 | - | 1,84 | 1,77 | 3,09 | - | 1,97 | - | 0,88 | - | - | - | - | - |
| 52 | - | 1,67 | 2,07 | - | 1,83 | 1,76 | 3,05 | - | 1,98 | - | 0,94 | - | - | - | - | - |
| 53 | - | 1,64 | 2,10 | - | 1,83 | 1,74 | 3,02 | - | 1,99 | - | 0,99 | - | - | - | - | - |
| 54 | - | 1,59 | 2,13 | - | 1,73 | 1,82 | 2,66 | - | 2,00 | - | 1,03 | 0,35 | - | - | - | - |
| 55 | - | 1,54 | 2,17 | - | 3,51 | - | 2,58 | - | 2,02 | - | 1,07 | 0,42 | - | - | - | - |
| 56 | - | 1,48 | 2,21 | - | 3,50 | - | 2,51 | - | 2,05 | - | 1,09 | 0,48 | - | - | - | - |
| 57 | - | 1,41 | 2,26 | - | 3,47 | - | 2,45 | - | 2,07 | - | 1,12 | 0,55 | - | - | - | - |
| 58 | - | 1,33 | 2,33 | - | 3,11 | - | 2,38 | - | 2,11 | - | 1,14 | 0,61 | - | - | 0,34 | - |
| 59 | - | 1,24 | 2,40 | - | 2,99 | - | 2,32 | - | 2,16 | - | 1,14 | 0,66 | - | - | 0,42 | - |
| 60 | - | 1,14 | 2,49 | - | 2,55 | - | 2,26 | - | 2,22 | - | 1,14 | 0,73 | - | - | 0,48 | 0,33 |
| 61 | 1,31 | - | 2,60 | - | 2,47 | - | 2,18 | - | 2,30 | - | 1,14 | - | 0,67 | 0,36 | - | 0,30 |
| 62 | 1,41 | - | 2,99 | - | 2,39 | - | 2,13 | - | 2,37 | - | 1,13 | - | 0,60 | 0,30 | - | - |
| 63 | 1,48 | - | 3,30 | - | 2,33 | - | 2,09 | - | 2,43 | - | 1,12 | - | 0,54 | - | - | - |
| 64 | 1,54 | - | 3,32 | - | 2,29 | - | 2,06 | - | 2,50 | - | 1,10 | - | 0,47 | - | - | - |
| 65 | 1,60 | - | 3,33 | - | 2,25 | - | 2,04 | - | 2,58 | - | 1,07 | - | 0,40 | - | - | - |
| 66 | 1,65 | - | 1,71 | 1,64 | 2,21 | - | 2,02 | - | 2,66 | - | 1,04 | - | 0,33 | - | - | - |
| 67 | 1,69 | - | 1,67 | 1,69 | 2,19 | - | 2,00 | - | 3,00 | - | 1,00 | - | - | - | - | - |
| 68 | 1,73 | - | 1,67 | 1,71 | 2,16 | - | 1,99 | - | 3,03 | - | 0,95 | - | - | - | - | - |
| 69 | 1,76 | - | 1,72 | 1,68 | 2,14 | - | 1,98 | - | 3,06 | - | 0,91 | - | - | - | - | - |
| 70 | 1,78 | - | 1,68 | 1,73 | 2,13 | - | 1,97 | - | 3,10 | - | 0,85 | - | - | - | - | - |
| 71 | 1,80 | - | 1,69 | 1,74 | 2,12 | - | 1,97 | - | 3,13 | - | 0,81 | - | - | - | - | - |
| 72 | 1,82 | - | 1,74 | 1,69 | 2,11 | - | 1,96 | - | 3,93 | - | - | - | - | - | - | - |
| 73 | 1,83 | - | 1,75 | 1,69 | 2,10 | - | 1,96 | - | 3,94 | - | - | - | - | - | - | - |
| 74 | 1,84 | - | 1,75 | 1,70 | 2,10 | - | 1,96 | - | 3,94 | - | - | - | - | - | - | - |
| 75 | 1,84 | - | 1,75 | 1,70 | 2,09 | - | 1,96 | - | 3,93 | - | - | - | - | - | - | - |
| 76 | 1,85 | - | 1,71 | 1,76 | 2,09 | - | 1,97 | - | 3,94 | - | - | - | - | - | - | - |
| 77 | 1,86 | - | 1,71 | 1,76 | 2,08 | - | 1,97 | - | 3,93 | - | - | - | - | - | - | - |
| 78 | 1,86 | - | 1,72 | 1,76 | 2,08 | - | 1,97 | - | 3,93 | - | - | - | - | - | - | - |
| 79 | 1,87 | - | 1,72 | 1,76 | 2,08 | - | 1,97 | - | 3,93 | - | - | - | - | - | - | - |
| 80 | 2,04 | - | 1,72 | 1,76 | 2,08 | - | 1,97 | - | 3,93 | - | - | - | - | - | - | - |
| 81 | 1,88 | - | 1,72 | 1,76 | 2,08 | - | 1,97 | - | 3,93 | - | - | - | - | - | - | - |
| 82 | 1,88 | - | 1,72 | 1,76 | 2,07 | - | 1,98 | - | 3,93 | - | - | - | - | - | - | - |
| 83 | 1,88 | - | 1,73 | 1,76 | 2,07 | - | 1,98 | - | 3,94 | - | - | - | - | - | - | - |
| 84 | 1,89 | - | 1,73 | 1,77 | 2,07 | - | 1,98 | - | 3,94 | - | - | - | - | - | - | - |
| 85 | 1,89 | - | 1,73 | 1,76 | 2,07 | - | 1,98 | - | 3,94 | - | - | - | - | - | - | - |
| 86 | 1,89 | - | 1,76 | 1,73 | 2,07 | - | 1,98 | - | 3,94 | - | - | - | - | - | - | - |

**Table S11.** Electronic population of basins for the rearrangement of nitrones (*Z*)-**9**

| IRC | V(C1,C6) | V(C3,C4) | V1(C4,C5) | V2(C4C5) | V1C5,C6) | V2(C5,C6) | V1(C1,N2) | V2(C1,N2) | V1(C3,N2) | V2(C3,N2) | V(N2) | V(C1) | V(C3) | V(C4) | V(C5) | V(C6) |
|---|---|---|---|---|---|---|---|---|---|---|---|---|---|---|---|---|
| 36 | - | 1,79 | 2,04 | - | 1,75 | 1,82 | 3,98 | - | 1,96 | - | - | - | - | - | - | - |
| 37 | - | 1,78 | 2,04 | - | 1,75 | 1,82 | 3,98 | - | 1,96 | - | - | - | - | - | - | - |
| 38 | - | 1,78 | 2,03 | - | 1,75 | 1,82 | 3,98 | - | 1,95 | - | - | - | - | - | - | - |

| | | | | | | | | | | | | | | | | |
|---|---|---|---|---|---|---|---|---|---|---|---|---|---|---|---|---|
| 39 | - | 1,78 | 2,03 | - | 1,76 | 1,81 | 3,98 | - | 1,95 | - | - | - | - | - | - | - |
| 40 | - | 1,77 | 2,04 | - | 1,76 | 1,81 | 3,96 | - | 1,95 | - | - | - | - | - | - | - |
| 41 | - | 1,77 | 2,04 | - | 1,76 | 1,80 | 3,96 | - | 1,95 | - | - | - | - | - | - | - |
| 42 | - | 1,76 | 2,04 | - | 1,76 | 1,80 | 3,96 | - | 1,96 | - | - | - | - | - | - | - |
| 43 | - | 1,76 | 2,05 | - | 1,76 | 1,79 | 3,95 | - | 1,96 | - | - | - | - | - | - | - |
| 44 | - | 1,75 | 2,05 | - | 1,77 | 1,78 | 3,94 | - | 1,97 | - | - | - | - | - | - | - |
| 45 | - | 1,75 | 2,05 | - | 1,77 | 1,78 | 3,94 | - | 1,97 | - | - | - | - | - | - | - |
| 46 | - | 1,74 | 2,06 | - | 1,77 | 1,78 | 3,93 | - | 1,98 | - | - | - | - | - | - | - |
| 47 | - | 1,73 | 2,07 | - | 1,77 | 1,78 | 3,93 | - | 1,98 | - | - | - | - | - | - | - |
| 48 | - | 1,72 | 2,07 | - | 1,77 | 1,77 | 3,92 | - | 1,98 | - | - | - | - | - | - | - |
| 49 | - | 1,70 | 2,08 | - | 1,77 | 1,78 | 3,92 | - | 1,99 | - | - | - | - | - | - | - |
| 50 | - | 1,69 | 2,09 | - | 1,78 | 1,77 | 3,92 | - | 1,99 | - | - | - | - | - | - | - |
| 51 | - | 1,67 | 2,10 | - | 1,77 | 1,79 | 3,92 | - | 2,00 | - | - | - | - | - | - | - |
| 52 | - | 1,65 | 2,12 | - | 1,76 | 1,80 | 3,61 | - | 2,01 | - | - | 0,32 | - | - | - | - |
| 53 | - | 1,62 | 2,13 | - | 3,55 | - | 3,59 | - | 2,02 | - | - | 0,36 | - | - | - | - |
| 54 | - | 1,59 | 2,15 | - | 3,57 | - | 3,54 | - | 2,04 | - | - | 0,41 | - | - | - | - |
| 55 | - | 1,54 | 2,17 | - | 3,59 | - | 2,75 | - | 2,04 | - | 0,76 | 0,47 | - | - | - | - |
| 56 | - | 1,49 | 2,20 | - | 3,60 | - | 2,67 | - | 2,06 | - | 0,80 | 0,51 | - | - | - | - |
| 57 | - | 1,44 | 2,24 | - | 3,16 | - | 2,60 | - | 2,09 | - | 0,84 | 0,56 | - | - | 0,45 | - |
| 58 | - | 1,38 | 2,28 | - | 2,66 | - | 2,53 | - | 2,12 | - | 0,87 | 0,61 | - | - | 0,53 | - |
| 59 | - | 1,30 | 2,32 | - | 2,56 | - | 2,46 | - | 2,15 | - | 0,90 | 0,65 | - | - | 0,59 | - |
| 60 | - | 1,22 | 2,39 | - | 2,48 | - | 2,39 | - | 2,21 | - | 0,92 | 0,69 | - | - | 0,65 | - |
| 61 | - | 1,12 | 2,47 | - | 2,41 | - | 2,23 | - | 2,37 | - | 0,93 | 0,72 | - | - | 0,69 | - |
| 62 | - | 1,01 | 2,57 | - | 2,35 | - | 2,15 | - | 2,46 | - | 0,92 | 0,71 | - | - | 0,77 | - |
| 63 | 1,54 | - | 3,09 | - | 2,30 | - | 2,10 | - | 2,53 | - | 0,92 | - | 0,64 | 0,25 | - | - |
| 64 | 1,59 | - | 3,33 | - | 2,27 | - | 2,07 | - | 2,59 | - | 0,90 | - | 0,57 | - | - | - |
| 65 | 1,63 | - | 3,36 | - | 2,22 | - | 2,04 | - | 2,67 | - | 0,87 | - | 0,51 | - | - | - |
| 66 | 1,68 | - | 3,36 | - | 2,20 | - | 2,01 | - | 2,75 | - | 0,84 | - | 0,44 | - | - | - |
| 67 | 1,71 | - | 3,36 | - | 2,17 | - | 1,99 | - | 2,85 | - | 0,80 | - | 0,37 | - | - | - |
| 68 | 1,74 | - | 1,79 | 1,60 | 2,15 | - | 1,98 | - | 2,91 | - | 0,78 | - | 0,31 | - | - | - |
| 69 | 1,76 | - | 1,77 | 1,62 | 2,13 | - | 1,97 | - | 3,97 | - | - | - | - | - | - | - |
| 70 | 1,78 | - | 1,77 | 1,64 | 2,12 | - | 1,97 | - | 3,96 | - | pop. | - | - | - | - | - |
| 71 | 1,80 | - | 1,77 | 1,65 | 2,10 | - | 1,96 | - | 3,96 | - | pop. | - | - | - | - | - |
| 72 | 1,82 | - | 1,77 | 1,67 | 2,10 | - | 1,95 | - | 3,96 | - | pop. | - | - | - | - | - |
| 73 | 1,83 | - | 1,76 | 1,67 | 2,09 | - | 1,95 | - | 3,96 | - | pop. | - | - | - | - | - |
| 74 | 1,84 | - | 1,76 | 1,68 | 2,08 | - | 1,95 | - | 3,96 | - | pop. | - | - | - | - | - |
| 75 | 1,84 | - | 1,76 | 1,69 | 2,08 | - | 1,95 | - | 3,96 | - | pop. | - | - | - | - | - |
| 76 | 1,86 | - | 1,75 | 1,70 | 2,07 | - | 1,95 | - | 3,96 | - | pop. | - | - | - | - | - |
| 77 | 1,86 | - | 1,75 | 1,70 | 2,07 | - | 1,94 | - | 3,97 | - | pop. | - | - | - | - | - |
| 78 | 1,87 | - | 1,75 | 1,71 | 2,07 | - | 1,95 | - | 3,97 | - | pop. | - | - | - | - | - |
| 79 | 1,87 | - | 1,75 | 1,71 | 2,07 | - | 1,95 | - | 3,97 | - | pop. | - | - | - | - | - |
| 80 | 1,88 | - | 1,75 | 1,72 | 2,07 | - | 1,95 | - | 3,97 | - | pop. | - | - | - | - | - |
| 81 | 1,88 | - | 1,75 | 1,72 | 2,07 | - | 1,95 | - | 3,97 | - | pop. | - | - | - | - | - |
| 82 | 1,88 | - | 1,75 | 1,72 | 2,07 | - | 1,95 | - | 3,97 | - | pop. | - | - | - | - | - |
| 83 | 1,88 | - | 1,74 | 1,73 | 2,06 | - | 1,96 | - | 3,98 | - | pop. | - | - | - | - | - |
| 84 | 1,89 | - | 1,75 | 1,73 | 2,06 | - | 1,96 | - | 3,98 | - | pop. | - | - | - | - | - |
| 85 | 1,89 | - | 1,75 | 1,73 | 2,06 | - | 1,96 | - | 3,97 | - | pop. | - | - | - | - | - |
| 86 | 1,89 | - | 1,75 | 1,73 | 2,06 | - | 1,96 | - | 3,98 | - | pop. | - | - | - | - | - |

**Table S12.** Electronic population of basins for the rearrangement of nitrones (*E*)-**9**

| IRC | V(C1,C6) | V(C3,C4) | V1(C4,C5) | V2(C4C5) | V1C5,C6) | V2(C5,C6) | V1(C1,N2) | V2(C1,N2) | V1(C3,N2) | V2(C3,N2) | V(N2) | V(C1) | V(C3) | V(C4) | V(C5) | V(C6) |
|---|---|---|---|---|---|---|---|---|---|---|---|---|---|---|---|---|
| 36 | - | 1.78 | 2.03 | - | 1.75 | 1.78 | 3.94 | - | 1.96 | - | - | - | - | - | - | - |
| 37 | - | 1.78 | 2.03 | - | 1.74 | 1.78 | 3.94 | - | 1.96 | - | - | - | - | - | - | - |
| 38 | - | 1.78 | 2.03 | - | 1.79 | 1.74 | 3.94 | - | 1.96 | - | - | - | - | - | - | - |
| 39 | - | 1.77 | 2.03 | - | 1.73 | 1.79 | 3.93 | - | 1.96 | - | - | - | - | - | - | - |
| 40 | - | 1.77 | 2.03 | - | 1.73 | 1.80 | 3.94 | - | 1.96 | - | - | - | - | - | - | - |
| 41 | - | 1.77 | 2.04 | - | 1.73 | 1.79 | 3.93 | - | 1.96 | - | - | - | - | - | - | - |
| 42 | - | 1.77 | 2.04 | - | 1.72 | 1.80 | 3.92 | - | 1.96 | - | - | - | - | - | - | - |
| 43 | - | 1.77 | 2.04 | - | 1.72 | 1.80 | 3.93 | - | 1.95 | - | - | - | - | - | - | - |
| 44 | - | 1.76 | 2.04 | - | 1.71 | 1.80 | 3.93 | - | 1.96 | - | - | - | - | - | - | - |
| 45 | - | 1.76 | 2.05 | - | 1.70 | 1.80 | 3.92 | - | 1.95 | - | - | - | - | - | - | - |
| 46 | - | 1.75 | 2.05 | - | 1.70 | 1.80 | 3.92 | - | 1.95 | - | - | - | - | - | - | - |
| 47 | - | 1.74 | 2.05 | - | 1.69 | 1.80 | 3.91 | - | 1.95 | - | - | - | - | - | - | - |
| 48 | - | 1.73 | 2.06 | - | 1.69 | 1.81 | 3.92 | - | 1.95 | - | - | - | - | - | - | - |
| 49 | - | 1.72 | 2.07 | - | 1.68 | 1.81 | 3.90 | - | 1.96 | - | - | - | - | - | - | - |
| 50 | - | 1.71 | 2.08 | - | 1.82 | 1.67 | 3.09 | - | 1.95 | - | 0.84 | - | - | - | - | - |
| 51 | - | 1.69 | 2.09 | - | 1.83 | 1.65 | 3.05 | - | 1.96 | - | 0.88 | - | - | - | - | - |
| 52 | - | 1.67 | 2.11 | - | 1.84 | 1.64 | 3.00 | - | 1.97 | - | 0.94 | - | - | - | - | - |
| 53 | - | 1.64 | 2.13 | - | 1.78 | 1.69 | 2.97 | - | 1.98 | - | 0.99 | - | - | - | - | - |
| 54 | - | 1.60 | 2.16 | - | 3.45 | - | 2.68 | - | 1.99 | - | 1.04 | 0.28 | - | - | - | - |
| 55 | - | 1.56 | 2.19 | - | 3.46 | - | 2.59 | - | 2.00 | - | 1.08 | 0.35 | - | - | - | - |
| 56 | - | 1.51 | 2.22 | - | 3.46 | - | 2.51 | - | 2.02 | - | 1.12 | 0.41 | - | - | - | - |
| 57 | - | 1.45 | 2.27 | - | 3.05 | - | 2.44 | - | 2.04 | - | 1.15 | 0.47 | - | - | 0.42 | - |
| 58 | - | 1.38 | 2.32 | - | 2.94 | - | 2.38 | - | 2.07 | - | 1.18 | 0.52 | - | - | 0.52 | - |
| 59 | - | 1.31 | 2.40 | - | 2.61 | - | 2.32 | - | 2.11 | - | 1.20 | 0.56 | - | - | 0.60 | - |
| 60 | - | 1.22 | 2.72 | - | 2.56 | - | 2.26 | - | 2.16 | - | 1.21 | 0.60 | - | - | 0.66 | - |
| 61 | 1.35 | 1.11 | 2.81 | - | 2.43 | - | 2.17 | - | 2.24 | - | 1.21 | - | - | - | - | - |
| 62 | 1.43 | 1.00 | 2.89 | - | 2.37 | - | 2.12 | - | 2.30 | - | 1.21 | - | - | - | - | - |
| 63 | 1.49 | - | 2.98 | - | 2.32 | - | 2.08 | - | 2.36 | - | 1.19 | - | 0.58 | 0.30 | - | - |
| 64 | 1.55 | - | 3.29 | - | 2.27 | - | 2.05 | - | 2.43 | - | 1.17 | - | 0.52 | - | - | - |
| 65 | 1.60 | - | 3.31 | - | 2.23 | - | 2.02 | - | 2.50 | - | 1.15 | - | 0.45 | - | - | - |
| 66 | 1.64 | - | 3.32 | - | 2.20 | - | 2.00 | - | 2.57 | - | 1.12 | - | 0.38 | - | - | - |
| 67 | 1.67 | - | 3.32 | - | 2.17 | - | 1.98 | - | 2.65 | - | 1.08 | - | 0.30 | - | - | - |
| 68 | 1.70 | - | 1.78 | 1.58 | 2.15 | - | 1.97 | - | 2.98 | - | 1.03 | - | - | - | - | - |
| 69 | 1.73 | - | 1.77 | 1.60 | 2.13 | - | 1.96 | - | 3.00 | - | 0.99 | - | - | - | - | - |
| 70 | 1.75 | - | 1.76 | 1.63 | 2.11 | - | 1.95 | - | 3.03 | - | 0.94 | - | - | - | - | - |
| 71 | 1.77 | - | 1.77 | 1.64 | 2.10 | - | 1.95 | - | 3.08 | - | 0.89 | - | - | - | - | - |
| 72 | 1.79 | - | 1.77 | 1.65 | 2.09 | - | 1.94 | - | 3.11 | - | 0.84 | - | - | - | - | - |
| 73 | 1.80 | - | 1.65 | 1.77 | 2.08 | - | 1.95 | - | 3.92 | - | - | - | - | - | - | - |
| 74 | 1.81 | - | 1.66 | 1.77 | 2.08 | - | 1.94 | - | 3.94 | - | - | - | - | - | - | - |
| 75 | 1.82 | - | 1.67 | 1.77 | 2.07 | - | 1.94 | - | 3.95 | - | - | - | - | - | - | - |
| 76 | 1.82 | - | 1.67 | 1.77 | 2.07 | - | 1.94 | - | 3.95 | - | - | - | - | - | - | - |
| 77 | 1.83 | - | 1.68 | 1.78 | 2.06 | - | 1.94 | - | 3.95 | - | - | - | - | - | - | - |
| 78 | 1.84 | - | 1.68 | 1.78 | 2.06 | - | 1.94 | - | 3.95 | - | - | - | - | - | - | - |
| 79 | 1.84 | - | 1.69 | 1.78 | 2.06 | - | 1.94 | - | 3.95 | - | - | - | - | - | - | - |
| 80 | 1.84 | - | 1.69 | 1.77 | 2.06 | - | 1.94 | - | 3.95 | - | - | - | - | - | - | - |

| | | | | | | | | | | | | | | | | |
|---|---|---|---|---|---|---|---|---|---|---|---|---|---|---|---|---|
| 81 | 1.85 | - | 1.69 | 1.78 | 2.06 | - | 1.94 | - | 3.96 | - | - | - | - | - | - | - |
| 82 | 1.85 | - | 1.69 | 1.78 | 2.05 | - | 1.94 | - | 3.96 | - | - | - | - | - | - | - |
| 83 | 1.85 | - | 1.70 | 1.78 | 2.05 | - | 1.94 | - | 3.96 | - | - | - | - | - | - | - |
| 84 | 1.85 | - | 1.78 | 1.70 | 2.05 | - | 1.94 | - | 3.95 | - | - | - | - | - | - | - |
| 85 | 1.85 | - | 1.70 | 1.77 | 2.05 | - | 1.94 | - | 3.95 | - | - | - | - | - | - | - |
| 86 | | | 1.70 | 1.77 | 2.05 | - | 1.95 | - | 3.95 | - | | | - | - | - | - |

**Table S13.** Electronic population of basins for the rearrangement of nitrones **10**

| IRC | V(C1,C6) | V(C3,C4) | V1(C4,C5) | V2(C4C5) | V1C5,C6) | V2(C5,C6) | V1(C1,N2) | V2(C1,N2) | V1(C3,N2) | V2(C3,N2) | V(N2) | V(C1) | V(C3) | V(C4) | V(C5) | V(C6) |
|---|---|---|---|---|---|---|---|---|---|---|---|---|---|---|---|---|
| 36 | 1.80 | - | 1.82 | 1.86 | 2.10 | - | 1.95 | - | 3.96 | - | - | - | - | - | - | - |
| 37 | 1.80 | - | 1.81 | 1.86 | 2.10 | - | 1.94 | - | 3.96 | - | - | - | - | - | - | - |
| 38 | 1.80 | - | 1.81 | 1.86 | 2.10 | - | 1.94 | - | 3.96 | - | - | - | - | - | - | - |
| 39 | 1.79 | - | 1.81 | 1.86 | 2.10 | - | 1.94 | - | 3.95 | - | - | - | - | - | - | - |
| 40 | 1.78 | - | 1.80 | 1.86 | 2.10 | - | 1.93 | - | 3.14 | - | 0.84 | - | - | - | - | - |
| 41 | 1.78 | - | 1.80 | 1.85 | 2.11 | - | 1.93 | - | 3.09 | - | 0.90 | - | - | - | - | - |
| 42 | 1.77 | - | 1.79 | 1.84 | 2.12 | - | 1.92 | - | 3.04 | - | 0.96 | - | - | - | - | - |
| 43 | 1.77 | - | 1.78 | 1.83 | 2.12 | - | 1.91 | - | 3.00 | - | 1.03 | - | - | - | - | - |
| 44 | 1.76 | - | 1.77 | 1.82 | 2.13 | - | 1.91 | - | 2.96 | - | 1.09 | - | - | - | - | - |
| 45 | 1.75 | - | 1.77 | 1.81 | 2.14 | - | 1.90 | - | 2.93 | - | 1.15 | - | - | - | - | - |
| 46 | 1.74 | - | 1.75 | 1.81 | 2.15 | - | 1.90 | - | 2.89 | - | 1.21 | - | - | - | - | - |
| 47 | 1.73 | - | 1.73 | 1.82 | 2.16 | - | 1.90 | - | 2.87 | - | 1.27 | - | - | - | - | - |
| 48 | 1.71 | - | 3.51 | - | 2.18 | - | 1.90 | - | 2.84 | - | 1.34 | - | - | - | - | - |
| 49 | 1.70 | - | 3.50 | - | 2.20 | - | 1.90 | - | 2.52 | - | 1.40 | - | 0.31 | - | - | - |
| 50 | 1.68 | - | 3.50 | - | 2.21 | - | 1.90 | - | 2.44 | - | 1.45 | - | 0.38 | - | - | - |
| 51 | 1.67 | - | 3.17 | - | 2.23 | - | 1.91 | - | 2.37 | - | 1.51 | - | 0.44 | 0.32 | - | - |
| 52 | 1.65 | - | 3.07 | - | 2.26 | - | 1.91 | - | 2.31 | - | 1.56 | - | 0.49 | 0.41 | - | - |
| 53 | 1.63 | - | 2.99 | - | 2.28 | - | 1.92 | - | 2.25 | - | 1.61 | - | 0.55 | 0.48 | - | - |
| 54 | 1.61 | - | 2.93 | - | 2.31 | - | 1.93 | - | 2.20 | - | 1.65 | - | 0.60 | 0.54 | - | - |
| 55 | 1.58 | 1.23 | 2.87 | - | 2.34 | - | 1.94 | - | 2.16 | - | 1.69 | - | - | - | - | - |
| 56 | 1.55 | 1.31 | 2.80 | - | 2.37 | - | 1.96 | - | 2.11 | - | 1.71 | - | - | - | - | - |
| 57 | 1.51 | 1.38 | 2.74 | - | 2.42 | - | 1.98 | - | 2.08 | - | 1.73 | - | - | - | - | - |
| 58 | 1.45 | 1.44 | 2.67 | - | 2.49 | - | 2.02 | - | 2.04 | - | 1.72 | - | - | - | - | - |
| 59 | 1.38 | 1.48 | 2.55 | - | 2.63 | - | 2.06 | - | 2.01 | - | 1.70 | - | - | - | - | - |
| 60 | 1.30 | 1.52 | 2.45 | - | 2.75 | - | 2.12 | - | 1.99 | - | 1.67 | - | - | - | - | - |
| 61 | 1.20 | 1.55 | 2.40 | - | 2.83 | - | 2.17 | - | 1.97 | - | 1.64 | - | - | - | - | - |
| 62 | - | 1.57 | 2.35 | - | 2.90 | - | 2.22 | - | 1.95 | - | 1.60 | 0.60 | - | - | 0.51 | - |
| 63 | - | 1.60 | 2.32 | - | 2.98 | - | 2.27 | - | 1.94 | - | 1.56 | 0.54 | - | - | 0.46 | - |
| 64 | - | 1.62 | 2.29 | - | 3.06 | - | 2.33 | - | 1.93 | - | 1.52 | 0.49 | - | - | 0.38 | - |
| 65 | - | 1.64 | 2.27 | - | 3.14 | - | 2.39 | - | 1.92 | - | 1.47 | 0.44 | - | - | 0.32 | - |
| 66 | - | 1.66 | 2.24 | - | 3.46 | - | 2.46 | - | 1.92 | - | 1.41 | 0.38 | - | - | - | - |
| 67 | - | 1.68 | 2.22 | - | 3.46 | - | 2.54 | - | 1.92 | - | 1.35 | 0.31 | - | - | - | - |
| 68 | - | 1.70 | 2.20 | - | 1.71 | 1.79 | 2.86 | - | 1.92 | - | 1.29 | - | - | - | - | - |
| 69 | - | 1.71 | 2.19 | - | 1.79 | 1.73 | 2.88 | - | 1.91 | - | 1.24 | - | - | - | - | - |
| 70 | - | 1.73 | 2.17 | - | 1.79 | 1.75 | 2.92 | - | 1.91 | - | 1.17 | - | - | - | - | - |
| 71 | - | 1.74 | 2.16 | - | 1.79 | 1.76 | 2.95 | - | 1.92 | - | 1.11 | - | - | - | - | - |
| 72 | - | 1.75 | 2.15 | - | 1.80 | 1.76 | 2.98 | - | 1.92 | - | 1.06 | - | - | - | - | - |

| | | | | | | | | | | | | | | | | |
|---|---|---|---|---|---|---|---|---|---|---|---|---|---|---|---|---|
| 73 | - | 1.76 | 2.14 | - | 1.81 | 1.77 | 3.02 | - | 1.92 | - | 0.99 | - | - | - | - | - |
| 74 | - | 1.77 | 2.13 | - | 1.82 | 1.78 | 3.06 | - | 1.93 | - | 0.94 | - | - | - | - | - |
| 75 | - | 1.78 | 2.13 | - | 1.83 | 1.78 | 3.11 | - | 1.93 | - | 0.87 | - | - | - | - | - |
| 76 | - | 1.78 | 2.12 | - | 1.83 | 1.79 | 3.14 | - | 1.93 | - | 0.84 | - | - | - | - | - |
| 77 | - | 1.79 | 2.12 | - | 1.84 | 1.79 | 3.96 | - | 1.94 | - | - | - | - | - | - | - |
| 78 | - | 1.79 | 2.11 | - | 1.84 | 1.80 | 3.96 | - | 1.94 | - | - | - | - | - | - | - |
| 79 | - | 1.80 | 2.11 | - | 1.84 | 1.80 | 3.95 | - | 1.94 | - | - | - | - | - | - | - |
| 80 | - | 1.80 | 2.10 | - | 1.85 | 1.80 | 3.96 | - | 1.95 | - | - | - | - | - | - | - |
| 81 | - | 1.80 | 2.10 | - | 1.85 | 1.81 | 3.95 | - | 1.95 | - | - | - | - | - | - | - |
| 82 | - | 1.81 | 2.10 | - | 1.85 | 1.81 | 3.95 | - | 1.95 | - | - | - | - | - | - | - |
| 83 | - | 1.81 | 2.10 | - | 1.85 | 1.81 | 3.95 | - | 1.96 | - | - | - | - | - | - | - |
| 84 | - | 1.81 | 2.10 | - | 1.86 | 1.82 | 3.95 | - | 1.96 | - | - | - | - | - | - | - |
| 85 | - | 1.81 | 2.10 | - | 1.86 | 1.82 | 3.95 | - | 1.96 | - | - | - | - | - | - | - |
| 86 | - | 1.81 | 2.10 | - | 1.86 | 1.82 | 3.96 | - | 1.95 | - | - | - | - | - | - | - |

**Table S14.** Electronic population of basins for the rearrangement of nitrones (*Z*)-**11**

| IRC | V(C1,C6) | V(C3,C4) | V1(C4,C5) | V2(C4C5) | V1C5,C6) | V2(C5,C6) | V1(C1,N2) | V2(C1,N2) | V1(C3,N2) | V2(C3,N2) | V(N2) | V(C1) | V(C3) | V(C4) | V(C5) | V(C6) |
|---|---|---|---|---|---|---|---|---|---|---|---|---|---|---|---|---|
| 36 | - | 1.84 | 1.99 | - | 1.87 | 1.80 | 3.57 | 0.50 | 2.06 | - | - | - | - | - | - | - |
| 37 | - | 1.84 | 1.99 | - | 1.87 | 1.80 | 3.60 | 0.48 | 2.05 | - | - | - | - | - | - | - |
| 38 | - | 1.84 | 1.99 | - | 1.87 | 1.80 | 3.62 | 0.45 | 2.05 | - | - | - | - | - | - | - |
| 39 | - | 1.83 | 1.99 | - | 1.88 | 1.79 | 3.64 | 0.43 | 2.05 | - | - | - | - | - | - | - |
| 40 | - | 1.82 | 2.00 | - | 1.88 | 1.78 | 3.67 | 0.40 | 2.05 | - | - | - | - | - | - | - |
| 41 | - | 1.82 | 2.00 | - | 1.87 | 1.78 | 3.71 | 0.36 | 2.06 | - | - | - | - | - | - | - |
| 42 | - | 1.81 | 2.00 | - | 1.88 | 1.77 | 3.34 | 0.34 | 2.05 | - | - | 0.39 | - | - | - | - |
| 43 | - | 1.81 | 2.00 | - | 1.77 | 1.88 | 3.66 | - | 2.05 | - | - | 0.42 | - | - | - | - |
| 44 | - | 1.80 | 2.00 | - | 1.76 | 1.89 | 3.63 | - | 2.05 | - | - | 0.45 | - | - | - | - |
| 45 | - | 1.79 | 2.01 | - | 1.75 | 1.89 | 3.59 | - | 2.05 | - | - | 0.49 | - | - | - | - |
| 46 | - | 1.78 | 2.01 | - | 1.89 | 1.74 | 3.55 | - | 2.05 | - | - | 0.53 | - | - | - | - |
| 47 | - | 1.77 | 2.02 | - | 1.73 | 1.90 | 3.51 | - | 2.05 | - | - | 0.56 | - | - | - | - |
| 48 | - | 1.76 | 2.02 | - | 1.91 | 1.72 | 3.47 | - | 2.05 | - | - | 0.61 | - | - | - | - |
| 49 | - | 1.75 | 2.03 | - | 1.70 | 1.92 | 3.43 | - | 2.05 | - | - | 0.66 | - | - | - | - |
| 50 | - | 1.73 | 2.04 | - | 1.67 | 1.94 | 3.41 | - | 2.05 | - | - | 0.70 | - | - | - | - |
| 51 | - | 1.71 | 2.05 | - | 3.59 | - | 3.39 | - | 2.05 | - | - | 0.74 | - | - | - | - |
| 52 | - | 1.69 | 2.07 | - | 3.59 | - | 2.70 | - | 2.05 | - | 0.70 | 0.79 | - | - | - | - |
| 53 | - | 1.67 | 2.08 | - | 3.59 | - | 2.63 | - | 2.06 | - | 0.76 | 0.83 | - | - | - | - |
| 54 | - | 1.65 | 2.10 | - | 3.59 | - | 2.56 | - | 2.06 | - | 0.82 | 0.87 | - | - | - | - |
| 55 | - | 1.62 | 2.12 | - | 3.05 | - | 2.50 | - | 2.08 | - | 0.87 | 0.92 | - | - | - | 0.53 |
| 56 | - | 1.59 | 2.14 | - | 2.70 | - | 2.43 | - | 2.10 | - | 0.93 | 1.05 | - | - | 0.20 | 0.57 |
| 57 | 1.40 | 1.56 | 2.16 | - | 2.56 | - | 2.34 | - | 2.15 | - | 0.98 | - | - | - | - | 0.60 |
| 58 | 1.48 | 1.52 | 2.18 | - | 2.48 | - | 2.24 | - | 2.22 | - | 1.03 | - | - | - | - | 0.62 |
| 59 | 1.55 | 1.49 | 2.21 | - | 2.42 | - | 2.18 | - | 2.25 | - | 1.06 | - | - | - | - | 0.64 |
| 60 | 1.61 | 1.44 | 2.24 | - | 2.36 | - | 2.13 | - | 2.28 | - | 1.09 | - | - | - | - | 0.64 |
| 61 | 1.66 | 1.38 | 2.28 | - | 2.32 | - | 2.10 | - | 2.31 | - | 1.11 | - | - | - | - | 0.64 |
| 62 | 1.70 | 1.32 | 2.33 | - | 2.28 | - | 2.07 | - | 2.34 | - | 1.12 | - | - | - | - | 0.63 |
| 63 | 1.73 | 1.24 | 2.39 | - | 2.25 | - | 2.05 | - | 2.37 | - | 1.12 | - | - | - | - | 0.60 |

| | | | | | | | | | | | | | | | | |
|---|---|---|---|---|---|---|---|---|---|---|---|---|---|---|---|---|
| 64 | 1.76 | 1.15 | 2.46 | - | 2.23 | - | 2.03 | - | 2.41 | - | 1.11 | - | - | - | - | 0.58 |
| 65 | 1.78 | 1.05 | 2.55 | - | 2.21 | - | 2.02 | - | 2.46 | - | 1.09 | - | - | - | - | 0.54 |
| 66 | 1.81 | - | 2.67 | - | 2.19 | - | 2.02 | - | 2.51 | - | 1.06 | - | 0.68 | 0.26 | - | 0.48 |
| 67 | 1.82 | - | 3.21 | - | 2.17 | - | 2.01 | - | 2.57 | - | 1.02 | - | 0.62 | 0.21 | - | - |
| 68 | 1.84 | - | 3.41 | - | 2.16 | - | 2.00 | - | 2.63 | - | 0.98 | - | 0.57 | - | - | - |
| 69 | 1.85 | - | 3.41 | - | 2.15 | - | 1.99 | - | 2.71 | - | 0.94 | - | 0.51 | - | - | - |
| 70 | 1.87 | - | 3.40 | - | 2.13 | - | 1.98 | - | 2.78 | - | 0.89 | - | 0.46 | - | - | - |
| 71 | 1.88 | - | 1.78 | 1.64 | 2.12 | - | 1.98 | - | 2.84 | - | 0.87 | - | 0.41 | - | - | - |
| 72 | 1.89 | - | 1.76 | 1.65 | 2.11 | - | 1.98 | - | 3.73 | - | - | - | 0.36 | - | - | - |
| 73 | 1.90 | - | 1.75 | 1.66 | 2.10 | - | 1.98 | - | 4.07 | - | - | - | - | - | - | - |
| 74 | 1.91 | - | 1.75 | 1.67 | 2.10 | - | 1.98 | - | 4.07 | - | - | - | - | - | - | - |
| 75 | 1.91 | - | 1.75 | 1.67 | 2.10 | - | 1.98 | - | 4.06 | - | - | - | - | - | - | - |
| 76 | 1.92 | - | 1.76 | 1.68 | 2.09 | - | 1.97 | - | 4.06 | - | - | - | - | - | - | - |
| 77 | 1.93 | - | 1.74 | 1.68 | 2.09 | - | 1.98 | - | 4.06 | - | - | - | - | - | - | - |
| 78 | 1.93 | - | 1.74 | 1.69 | 2.08 | - | 1.98 | - | 4.06 | - | - | - | - | - | - | - |
| 79 | 1.94 | - | 1.75 | 1.69 | 2.08 | - | 1.97 | - | 4.06 | - | - | - | - | - | - | - |
| 80 | 1.94 | - | 1.75 | 1.70 | 2.07 | - | 1.98 | - | 4.05 | - | - | - | - | - | - | - |
| 81 | 1.95 | - | 1.74 | 1.70 | 2.07 | - | 1.98 | - | 4.06 | - | - | - | - | - | - | - |
| 82 | 1.95 | - | 1.74 | 1.70 | 2.06 | - | 1.98 | - | 4.06 | - | - | - | - | - | - | - |
| 83 | 1.96 | - | 1.74 | 1.70 | 2.07 | - | 1.98 | - | 4.06 | - | - | - | - | - | - | - |
| 84 | 1.96 | - | 1.74 | 1.71 | 2.07 | - | 1.98 | - | 4.07 | - | - | - | - | - | - | - |
| 85 | 1.96 | - | 1.71 | 1.74 | 2.06 | - | 1.98 | - | 4.07 | - | - | - | - | - | - | - |
| 86 | 1.96 | - | 1.72 | 1.74 | 2.06 | - | 1.98 | - | 4.08 | - | - | - | - | - | - | - |

**Table S15.** Electronic population of basins for the rearrangement of nitrones (*E*)-**11**

| IRC | V(C1,C6) | V(C3,C4) | V1(C4,C5) | V2(C4C5) | V1C5,C6) | V2(C5,C6) | V1(C1,N2) | V2(C1,N2) | V1(C3,N2) | V2(C3,N2) | V(N2) | V(C1) | V(C3) | V(C4) | V(C5) | V(C6) |
|---|---|---|---|---|---|---|---|---|---|---|---|---|---|---|---|---|
| 36 | - | 1.83 | 1.96 | | 3.66 | | 3.91 | - | 2.12 | - | - | - | - | - | - | - |
| 37 | - | 1.82 | 1.97 | | 3.65 | | 3.91 | - | 2.12 | - | - | - | - | - | - | - |
| 38 | - | 1.82 | 1.97 | | 3.64 | | 3.91 | - | 2.12 | - | - | - | - | - | - | - |
| 39 | - | 1.81 | 1.97 | | 3.64 | | 3.92 | - | 2.11 | - | - | - | - | - | - | - |
| 40 | - | 1.80 | 1.98 | | 3.64 | | 3.92 | - | 2.11 | - | - | - | - | - | - | - |
| 41 | - | 1.79 | 1.99 | | 3.63 | | 3.93 | - | 2.12 | - | - | - | - | - | - | - |
| 42 | - | 1.80 | 1.98 | | 3.62 | | 3.94 | - | 2.11 | - | - | - | - | - | - | - |
| 43 | - | 1.78 | 1.99 | | 3.62 | | 3.94 | - | 2.12 | - | - | - | - | - | - | - |
| 44 | - | 1.79 | 2.00 | | 3.60 | | 3.95 | - | 2.11 | - | - | - | - | - | - | - |
| 45 | - | 1.78 | 2.00 | | 3.60 | | 3.96 | - | 2.11 | - | - | - | - | - | - | - |
| 46 | - | 1.76 | 2.01 | | 3.59 | | 3.98 | - | 2.11 | - | - | - | - | - | - | - |
| 47 | - | 1.75 | 2.02 | | 3.58 | | 4.00 | - | 2.10 | - | - | - | - | - | - | - |
| 48 | - | 1.74 | 2.03 | | 3.56 | | 4.02 | - | 2.10 | - | - | - | - | - | - | - |
| 49 | - | 1.72 | 2.04 | | 3.55 | | 4.05 | - | 2.09 | - | - | - | - | - | - | - |
| 50 | - | 1.72 | 2.05 | | 3.53 | | 3.54 | - | 2.08 | - | - | 0.55 | - | - | - | - |
| 51 | - | 1.70 | 2.07 | | 3.52 | | 3.52 | - | 2.08 | - | - | 0.58 | - | - | - | - |
| 52 | - | 1.68 | 2.09 | | 3.50 | | 2.83 | - | 2.06 | - | 0.70 | 0.62 | - | - | - | - |
| 53 | - | 1.65 | 2.11 | | 3.46 | | 2.73 | - | 2.06 | - | 0.80 | 0.67 | - | - | - | - |
| 54 | - | 1.63 | 2.13 | | 3.45 | | 2.65 | - | 2.05 | - | 0.88 | 0.72 | - | - | - | - |
| 55 | - | 1.61 | 2.15 | | 3.43 | | 2.57 | - | 2.05 | - | 0.96 | 0.77 | - | - | - | - |
| 56 | - | 1.57 | 2.18 | | 3.18 | | 2.49 | - | 2.05 | - | 1.04 | 0.82 | - | - | 0.22 | - |

| | | | | | | | | | | | | | | | | |
|---|---|---|---|---|---|---|---|---|---|---|---|---|---|---|---|---|
| 57 | 1.17 | 1.54 | 2.21 | | 3.09 | | 2.42 | - | 2.05 | - | 1.11 | - | - | - | - | - |
| 58 | 1.28 | 1.50 | 2.25 | | 3.00 | | 2.35 | - | 2.06 | - | 1.18 | - | - | - | - | - |
| 59 | 1.37 | 1.45 | 2.30 | | 2.92 | | 2.30 | - | 2.07 | - | 1.23 | - | - | - | - | - |
| 60 | 1.46 | 1.39 | 2.36 | | 2.83 | | 2.24 | - | 2.09 | - | 1.27 | - | - | - | - | - |
| 61 | 1.54 | 1.33 | 2.42 | | 2.76 | | 2.19 | - | 2.13 | - | 1.30 | - | - | - | - | - |
| 62 | 1.60 | 1.24 | 2.72 | | 2.45 | | 2.14 | - | 2.18 | - | 1.31 | - | - | - | - | - |
| 63 | 1.65 | 1.15 | 2.79 | | 2.39 | | 2.10 | - | 2.22 | - | 1.31 | - | - | - | - | - |
| 64 | 1.69 | 1.05 | 2.86 | | 2.35 | | 2.08 | - | 2.28 | - | 1.29 | - | - | - | - | - |
| 65 | 1.73 | 0.93 | 2.93 | | 2.31 | | 2.06 | - | 2.34 | - | 1.26 | - | - | - | - | - |
| 66 | 1.77 | - | 3.03 | | 2.28 | | 2.04 | - | 2.41 | - | 1.21 | - | 0.58 | 0.18 | - | - |
| 67 | 1.79 | - | 3.09 | | 2.25 | | 2.03 | - | 2.50 | - | 1.16 | - | 0.47 | 0.19 | - | - |
| 68 | 1.82 | - | 3.28 | | 2.23 | | 2.03 | - | 2.58 | - | 1.10 | - | 0.40 | - | - | - |
| 69 | 1.84 | - | 3.28 | | 2.21 | | 2.02 | - | 2.69 | - | 1.03 | - | 0.34 | - | - | - |
| 70 | 1.86 | - | 3.29 | | 2.18 | | 2.01 | - | 3.07 | - | 0.97 | - | - | - | - | - |
| 71 | 1.88 | - | 3.32 | | 2.16 | | 2.01 | - | 4.01 | - | - | - | - | - | - | - |
| 72 | 1.89 | - | 3.33 | | 2.15 | | 2.01 | - | 3.99 | - | - | - | - | - | - | - |
| 73 | 1.90 | - | 3.35 | | 2.14 | | 2.01 | - | 3.98 | - | - | - | - | - | - | - |
| 74 | 1.91 | - | 3.36 | | 2.13 | | 2.01 | - | 3.96 | - | - | - | - | - | - | - |
| 75 | 1.92 | - | 3.37 | | 2.12 | | 2.01 | - | 3.95 | - | - | - | - | - | - | - |
| 76 | 1.93 | - | 3.38 | | 2.11 | | 2.00 | - | 3.93 | - | - | - | - | - | - | - |
| 77 | 1.94 | - | 3.38 | | 2.10 | | 2.01 | - | 3.92 | - | - | - | - | - | - | - |
| 78 | 1.95 | - | 3.40 | | 2.10 | | 2.01 | - | 3.90 | - | - | - | - | - | - | - |
| 79 | 1.96 | - | 3.48 | | 2.09 | | 2.01 | - | 3.89 | - | - | - | - | - | - | - |
| 80 | 1.96 | - | 3.41 | | 2.09 | | 2.01 | - | 3.89 | - | - | - | - | - | - | - |
| 81 | 1.97 | - | 3.41 | | 2.08 | | 2.01 | - | 3.90 | - | - | - | - | - | - | - |
| 82 | 1.97 | - | 3.41 | | 2.08 | | 2.01 | - | 3.90 | - | - | - | - | - | - | - |
| 83 | 1.97 | - | 3.42 | | 2.08 | | 2.01 | - | 3.89 | - | - | - | - | - | - | - |
| 84 | 1.97 | - | 3.43 | | 2.07 | | 2.02 | - | 3.89 | - | - | - | - | - | - | - |
| 85 | 1.98 | - | 3.43 | | 2.07 | | 2.02 | - | 3.89 | - | - | - | - | - | - | - |
| 86 | 1.99 | - | 3.42 | | 2.07 | | 2.02 | - | 3.89 | - | - | - | - | - | - | - |

**Table S16**. Electronic population of basins for the rearrangement of nitrones **12**

| IRC | V(C1,C6) | V(C3,C4) | V1(C4,C5) | V2(C4C5) | V1C5,C6) | V2(C5,C6) | V1(C1,N2) | V2(C1,N2) | V1(C3,N2) | V2(C3,N2) | V(N2) | V(C1) | V(C3) | V(C4) | V(C5) | V(C6) |
|---|---|---|---|---|---|---|---|---|---|---|---|---|---|---|---|---|
| 36 | 1.82 | - | 1.76 | 1.69 | 2.01 | - | 2.00 | | 3.99 | | - | - | - | - | - | - |
| 37 | 1.82 | - | 1.76 | 1.69 | 2.01 | - | 2.01 | | 4.00 | | - | - | - | - | - | - |
| 38 | 1.82 | - | 1.76 | 1.69 | 2.01 | - | 2.00 | | 4.00 | | - | - | - | - | - | - |
| 39 | 1.81 | - | 1.76 | 1.68 | 2.01 | - | 2.00 | | 3.99 | | - | - | - | - | - | - |
| 40 | 1.81 | - | 1.76 | 1.68 | 2.02 | - | 2.00 | | 4.00 | | - | - | - | - | - | - |
| 41 | 1.80 | - | 1.75 | 1.68 | 2.02 | - | 2.00 | | 4.01 | | - | - | - | - | - | - |
| 42 | 1.79 | - | 1.75 | 1.67 | 2.03 | - | 1.99 | | 4.02 | | - | - | - | - | - | - |
| 43 | 1.79 | - | 1.75 | 1.67 | 2.04 | - | 1.99 | | 4.03 | | - | - | - | - | - | - |
| 44 | 1.77 | - | 1.75 | 1.66 | 2.04 | - | 1.99 | | 4.03 | | - | - | - | - | - | - |
| 45 | 1.76 | - | 1.65 | 1.75 | 2.05 | - | 1.98 | | 2.92 | | 0.74 | - | 0.40 | - | - | - |
| 46 | 1.75 | - | 1.64 | 1.74 | 2.06 | - | 1.98 | | 2.89 | | 0.77 | - | 0.43 | - | - | - |
| 47 | 1.74 | - | 1.63 | 1.74 | 2.07 | - | 1.98 | | 2.81 | | 0.83 | - | 0.47 | - | - | - |

| | | | | | | | | | | | | | | |
|---|---|---|---|---|---|---|---|---|---|---|---|---|---|---|
| 48 | 1.72 | - | 1.61 | 1.76 | 2.08 | - | 1.98 | 2.73 | 0.90 | - | 0.51 | - | - | - |
| 49 | 1.70 | - | 3.34 | - | 2.10 | - | 1.98 | 2.65 | 0.96 | - | 0.55 | - | - | - |
| 50 | 1.69 | - | 3.33 | - | 2.11 | - | 1.98 | 2.59 | 1.01 | - | 0.59 | - | - | - |
| 51 | 1.67 | - | 3.34 | - | 2.12 | - | 1.98 | 2.52 | 1.07 | - | 0.64 | - | - | - |
| 52 | 1.65 | - | 3.33 | - | 2.14 | - | 1.98 | 2.46 | 1.12 | - | 0.68 | - | - | - |
| 53 | 1.63 | - | 3.06 | - | 2.16 | - | 1.99 | 2.40 | 1.17 | - | 0.73 | 0.26 | - | - |
| 54 | 1.61 | - | 2.98 | - | 2.19 | - | 2.00 | 2.35 | 1.22 | - | 0.77 | 0.32 | - | - |
| 55 | 1.58 | 1.19 | 2.92 | - | 2.21 | - | 2.00 | 2.29 | 1.27 | - | - | - | - | - |
| 56 | 1.55 | 1.27 | 2.86 | - | 2.24 | - | 2.01 | 2.24 | 1.30 | - | - | - | - | - |
| 57 | 1.52 | 1.35 | 2.80 | - | 2.27 | - | 2.03 | 2.18 | 1.34 | - | - | - | - | - |
| 58 | 1.49 | 1.42 | 2.43 | - | 2.31 | - | 2.05 | 2.14 | 1.36 | - | - | - | - | 0.32 |
| 59 | 1.44 | 1.47 | 2.37 | - | 2.35 | - | 2.09 | 2.10 | 1.37 | - | - | - | - | 0.33 |
| 60 | 1.38 | 1.52 | 2.32 | - | 2.41 | - | 2.14 | 2.05 | 1.37 | - | - | - | - | 0.33 |
| 61 | 1.30 | 1.56 | 2.29 | - | 2.78 | - | 2.19 | 2.02 | 1.36 | - | - | - | - | - |
| 62 | 1.21 | 1.59 | 2.25 | - | 2.85 | - | 2.24 | 2.01 | 1.33 | - | - | - | - | - |
| 63 | 1.12 | 1.63 | 2.21 | - | 2.91 | - | 2.29 | 1.98 | 1.30 | - | - | - | - | - |
| 64 | 1.02 | 1.65 | 2.19 | - | 2.97 | - | 2.35 | 1.97 | 1.26 | - | - | - | - | - |
| 65 | - | 1.67 | 2.17 | - | 3.05 | - | 2.41 | 1.96 | 1.22 | 0.66 | - | - | 0.22 | - |
| 66 | - | 1.69 | 2.15 | - | 3.11 | - | 2.48 | 1.95 | 1.17 | 0.56 | - | - | 0.24 | - |
| 67 | - | 1.71 | 2.13 | - | 3.35 | - | 2.54 | 1.95 | 1.12 | 0.51 | - | - | - | - |
| 68 | - | 1.73 | 2.12 | - | 3.34 | - | 2.61 | 1.95 | 1.07 | 0.46 | - | - | - | - |
| 69 | - | 1.75 | 2.10 | - | 3.35 | - | 2.70 | 1.94 | 1.01 | 0.40 | - | - | - | - |
| 70 | - | 1.76 | 2.08 | - | 1.63 | 1.74 | 2.78 | 1.94 | 0.94 | 0.36 | - | - | - | - |
| 71 | - | 1.77 | 2.07 | - | 1.76 | 1.62 | 3.18 | 1.93 | 0.88 | - | - | - | - | - |
| 72 | - | 1.78 | 2.06 | - | 1.64 | 1.75 | 4.01 | 1.94 | - | - | - | - | - | - |
| 73 | - | 1.80 | 2.05 | - | 1.65 | 1.75 | 4.02 | 1.93 | - | - | - | - | - | - |
| 74 | - | 1.81 | 2.05 | - | 1.66 | 1.75 | 4.00 | 1.94 | - | - | - | - | - | - |
| 75 | - | 1.81 | 2.04 | - | 1.67 | 1.75 | 4.00 | 1.94 | - | - | - | - | - | - |
| 76 | - | 1.82 | 2.04 | - | 1.68 | 1.75 | 3.99 | 1.94 | - | - | - | - | - | - |
| 77 | - | 1.83 | 2.03 | - | 1.68 | 1.75 | 3.97 | 1.95 | - | - | - | - | - | - |
| 78 | - | 1.84 | 2.03 | - | 1.69 | 1.75 | 3.97 | 1.95 | - | - | - | - | - | - |
| 79 | - | 1.85 | 2.02 | - | 1.69 | 1.76 | 3.98 | 1.95 | - | - | - | - | - | - |
| 80 | - | 1.85 | 2.02 | - | 1.69 | 1.76 | 3.98 | 1.95 | - | - | - | - | - | - |
| 81 | - | 1.85 | 2.01 | - | 1.70 | 1.75 | 3.97 | 1.95 | - | - | - | - | - | - |
| 82 | - | 1.86 | 2.01 | - | 1.70 | 1.76 | 3.97 | 1.95 | - | - | - | - | - | - |
| 83 | - | 1.86 | 2.01 | - | 1.70 | 1.76 | 3.97 | 1.95 | - | - | - | - | - | - |
| 84 | - | 1.86 | 2.01 | - | 1.70 | 1.76 | 3.97 | 1.96 | - | - | - | - | - | - |
| 85 | - | 1.87 | 2.00 | - | 1.71 | 1.76 | 3.97 | 1.96 | - | - | - | - | - | - |
| 86 | - | 1.88 | 2.00 | - | 1.71 | 1.76 | 3.97 | 1.96 | - | - | - | - | - | - |

**Table S17.** Electronic population of basins for the rearrangement of nitrones **13**

| IRC | V(C1,C6) | V(C3,C4) | V1(C4,C5) | V2(C4C5) | V1C5,C6) | V2(C5,C6) | V1(C1,N2) | V2(C1,N2) | V1(C3,N2) | V2(C3,N2) | V(N2) | V(C1) | V(C3) | V(C4) | V(C5) | V(C6) |
|---|---|---|---|---|---|---|---|---|---|---|---|---|---|---|---|---|
| 36 | - | 1.86 | 2.00 | - | 1.76 | 1.71 | 4.08 | - | 1.94 | - | - | - | - | - | - | - |
| 37 | - | 1.85 | 2.00 | - | 1.76 | 1.70 | 4.08 | - | 1.94 | - | - | - | - | - | - | - |
| 38 | - | 1.85 | 2.01 | - | 1.76 | 1.69 | 4.08 | - | 1.93 | - | - | - | - | - | - | - |
| 39 | - | 1.84 | 2.01 | - | 1.76 | 1.69 | 4.08 | - | 1.93 | - | - | - | - | - | - | - |
| 40 | - | 1.84 | 2.01 | - | 1.76 | 1.68 | 4.09 | - | 1.93 | - | - | - | - | - | - | - |

| | | | | | | | | | | | | | | | | | |
|---|---|---|---|---|---|---|---|---|---|---|---|---|---|---|---|---|---|
| 41 | - | 1.83 | 2.02 | - | 1.76 | 1.68 | 4.08 | - | 1.93 | - | - | - | - | - | - | - | - |
| 42 | - | 1.83 | 2.02 | - | 1.76 | 1.68 | 4.09 | - | 1.93 | - | - | - | - | - | - | - | - |
| 43 | - | 1.82 | 2.02 | - | 1.76 | 1.67 | 4.09 | - | 1.93 | - | - | - | - | - | - | - | - |
| 44 | - | 1.82 | 2.03 | - | 1.75 | 1.67 | 4.10 | - | 1.93 | - | - | - | - | - | - | - | - |
| 45 | - | 1.81 | 2.04 | - | 1.76 | 1.66 | 4.10 | - | 1.93 | - | - | - | - | - | - | - | - |
| 46 | - | 1.80 | 2.05 | - | 1.66 | 1.75 | 4.11 | - | 1.93 | - | - | - | - | - | - | - | - |
| 47 | - | 1.79 | 2.05 | - | 1.75 | 1.65 | 4.07 | - | 1.93 | - | - | - | - | - | - | - | - |
| 48 | - | 1.77 | 2.06 | - | 1.64 | 1.75 | 3.26 | - | 1.93 | - | 0.88 | - | - | - | - | - | - |
| 49 | - | 1.76 | 2.07 | - | 1.63 | 1.75 | 3.23 | - | 1.94 | - | 0.91 | - | - | - | - | - | - |
| 50 | - | 1.75 | 2.08 | - | 1.77 | 1.60 | 2.81 | - | 1.94 | - | 0.97 | 0.38 | - | - | - | - | - |
| 51 | - | 1.74 | 2.10 | - | 3.34 | - | 2.73 | - | 1.94 | - | 1.03 | 0.43 | - | - | - | - | - |
| 52 | - | 1.72 | 2.11 | - | 3.34 | - | 2.65 | - | 1.94 | - | 1.08 | 0.48 | - | - | - | - | - |
| 53 | - | 1.71 | 2.13 | - | 3.34 | - | 2.57 | - | 1.96 | - | 1.13 | 0.53 | - | - | - | - | - |
| 54 | - | 1.68 | 2.15 | - | 3.34 | - | 2.51 | - | 1.96 | - | 1.16 | 0.58 | - | - | - | - | - |
| 55 | - | 1.66 | 2.17 | - | 3.06 | - | 2.44 | - | 1.98 | - | 1.21 | 0.63 | - | - | 0.27 | - | |
| 56 | - | 1.63 | 2.19 | - | 2.99 | - | 2.38 | - | 2.00 | - | 1.25 | 0.68 | - | - | 0.33 | - | |
| 57 | 1.10 | 1.61 | 2.22 | - | 2.93 | - | 2.32 | - | 2.01 | - | 1.29 | - | - | - | - | - | |
| 58 | 1.20 | 1.57 | 2.25 | - | 2.87 | - | 2.27 | - | 2.03 | - | 1.32 | - | - | - | - | - | |
| 59 | 1.28 | 1.54 | 2.28 | - | 2.81 | - | 2.22 | - | 2.05 | - | 1.33 | - | - | - | - | - | |
| 60 | 1.35 | 1.50 | 2.33 | - | 2.46 | - | 2.17 | - | 2.09 | - | 1.35 | - | - | - | - | 0.30 | |
| 61 | 1.42 | 1.45 | 2.38 | - | 2.38 | - | 2.10 | - | 2.15 | - | 1.34 | - | - | - | - | 0.31 | |
| 62 | 1.47 | 1.38 | 2.44 | - | 2.34 | - | 2.07 | - | 2.20 | - | 1.33 | - | - | - | - | 0.30 | |
| 63 | 1.52 | 1.31 | 2.79 | - | 2.29 | - | 2.05 | - | 2.24 | - | 1.31 | - | - | - | - | - | |
| 64 | 1.56 | 1.23 | 2.86 | - | 2.26 | - | 2.04 | - | 2.28 | - | 1.28 | - | - | - | - | - | |
| 65 | 1.59 | 1.14 | 2.91 | - | 2.23 | - | 2.02 | - | 2.34 | - | 1.24 | - | - | - | - | - | |
| 66 | 1.62 | - | 2.98 | - | 2.20 | - | 2.02 | - | 2.39 | - | 1.19 | - | 0.74 | 0.30 | - | - | |
| 67 | 1.64 | - | 3.09 | - | 2.18 | - | 2.01 | - | 2.44 | - | 1.15 | - | 0.71 | 0.18 | - | - | |
| 68 | 1.67 | - | 3.32 | - | 2.16 | - | 2.01 | - | 2.50 | - | 1.09 | - | 0.64 | - | - | - | |
| 69 | 1.68 | - | 3.32 | - | 2.14 | - | 2.00 | - | 2.57 | - | 1.05 | - | 0.59 | - | - | - | |
| 70 | 1.70 | - | 3.32 | - | 2.12 | - | 2.00 | - | 2.63 | - | 0.99 | - | 0.55 | - | - | - | |
| 71 | 1.72 | - | 3.33 | - | 2.11 | - | 2.00 | - | 2.70 | - | 0.93 | - | 0.51 | - | - | - | |
| 72 | 1.73 | - | 1.59 | 1.76 | 2.09 | - | 2.01 | - | 2.77 | - | 0.88 | - | 0.47 | - | - | - | |
| 73 | 1.76 | - | 1.62 | 1.75 | 2.08 | - | 2.01 | - | 2.85 | - | 0.82 | - | 0.42 | - | - | - | |
| 74 | 1.77 | - | 1.64 | 1.75 | 2.07 | - | 2.01 | - | 2.93 | - | 0.74 | - | 0.38 | - | - | - | |
| 75 | 1.78 | - | 1.75 | 1.64 | 2.06 | - | 2.01 | - | 3.31 | - | 0.73 | - | - | - | - | - | |
| 76 | 1.79 | - | 1.65 | 1.75 | 2.05 | - | 2.01 | - | 4.00 | - | - | - | - | - | - | - | |
| 77 | 1.80 | - | 1.66 | 1.75 | 2.04 | - | 2.02 | - | 3.99 | - | - | - | - | - | - | - | |
| 78 | 1.81 | - | 1.67 | 1.75 | 2.04 | - | 2.02 | - | 3.99 | - | - | - | - | - | - | - | |
| 79 | 1.82 | - | 1.67 | 1.75 | 2.03 | - | 2.02 | - | 3.98 | - | - | - | - | - | - | - | |
| 80 | 1.83 | - | 1.68 | 1.76 | 2.03 | - | 2.02 | - | 3.97 | - | - | - | - | - | - | - | |
| 81 | 1.83 | - | 1.68 | 1.76 | 2.03 | - | 2.03 | - | 3.96 | - | - | - | - | - | - | - | |
| 82 | 1.83 | - | 1.68 | 1.76 | 2.02 | - | 2.03 | - | 3.97 | - | - | - | - | - | - | - | |
| 83 | 1.84 | - | 1.68 | 1.76 | 2.01 | - | 2.03 | - | 3.96 | - | - | - | - | - | - | - | |
| 84 | 1.85 | - | 1.69 | 1.76 | 2.01 | - | 2.03 | - | 3.96 | - | - | - | - | - | - | - | |
| 85 | 1.85 | - | 1.69 | 1.76 | 2.01 | - | 2.04 | - | 3.95 | - | - | - | - | - | - | - | |
| 86 | 1.85 | - | 1.70 | 1.76 | 2.01 | - | 2.04 | - | 3.95 | - | - | - | - | - | - | - | |

## Graphical Representations of ELF Analyses

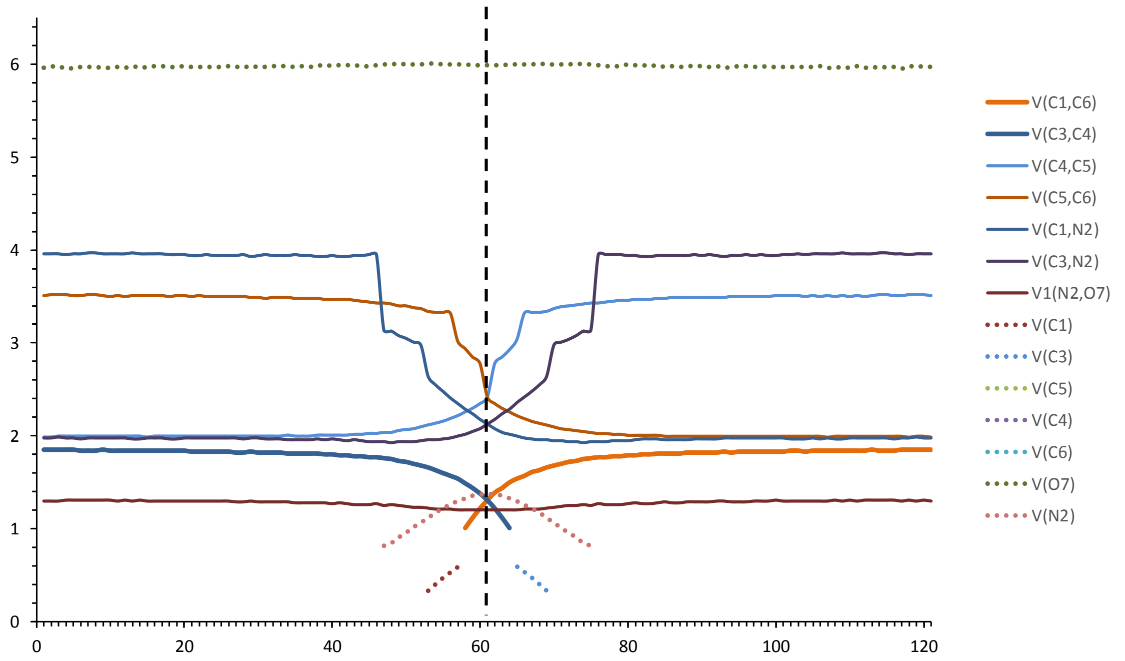


**Figura S3**. Graphical representation of ELF analysis for the rearrangement of nitrone **1**.

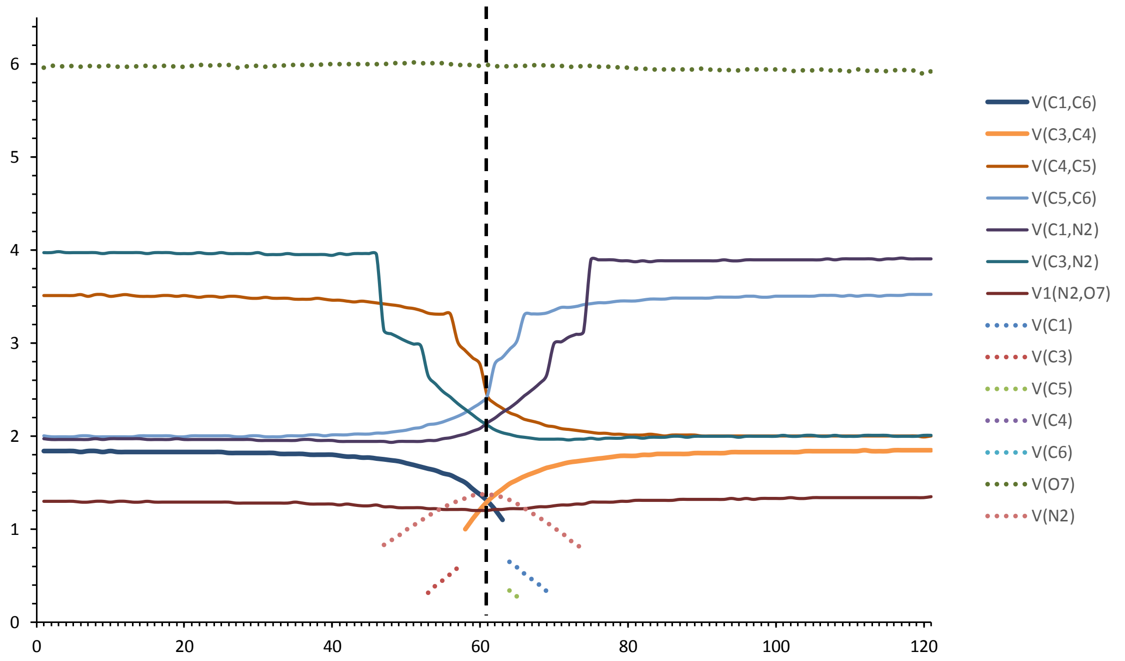


**Figura S4**. Graphical representation of ELF analysis for the rearrangement of nitrones **2**.

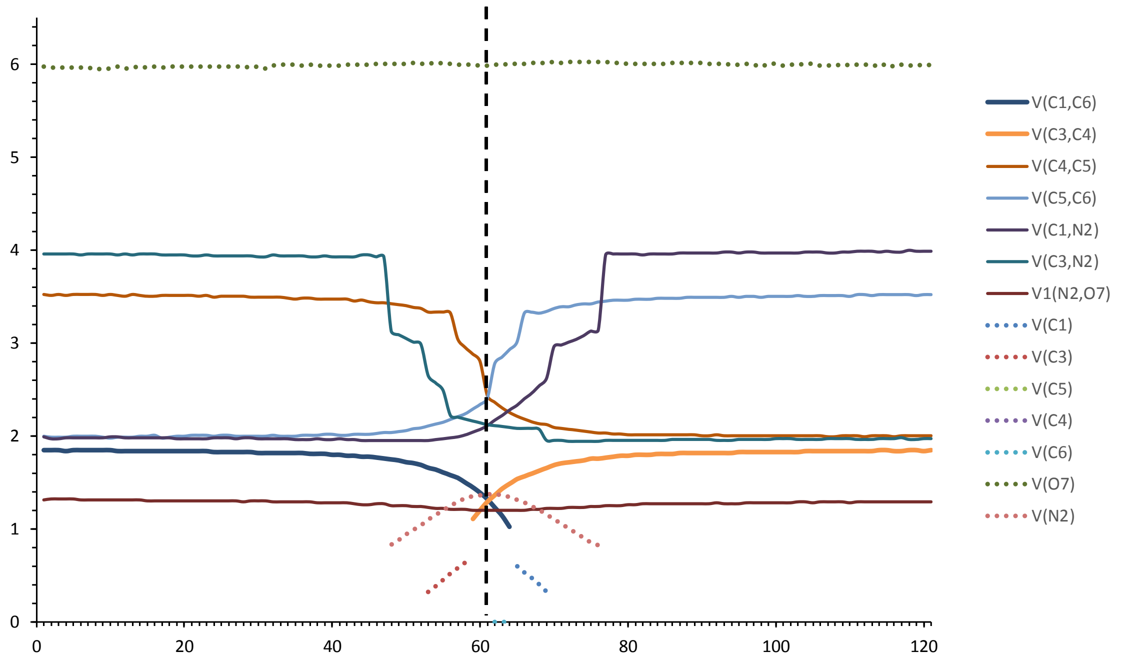


**Figura S5**. Graphical representation of ELF analysis for the rearrangement of nitrones **3**.

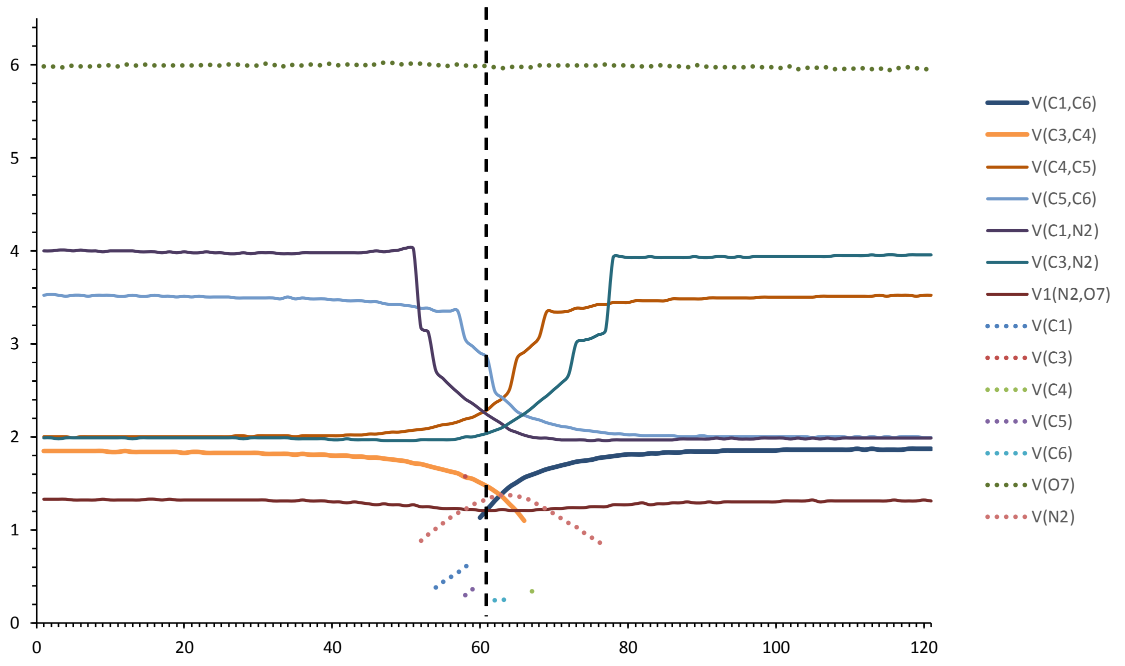


**Figura S6**. Graphical representation of ELF analysis for the rearrangement of nitrones **4**.

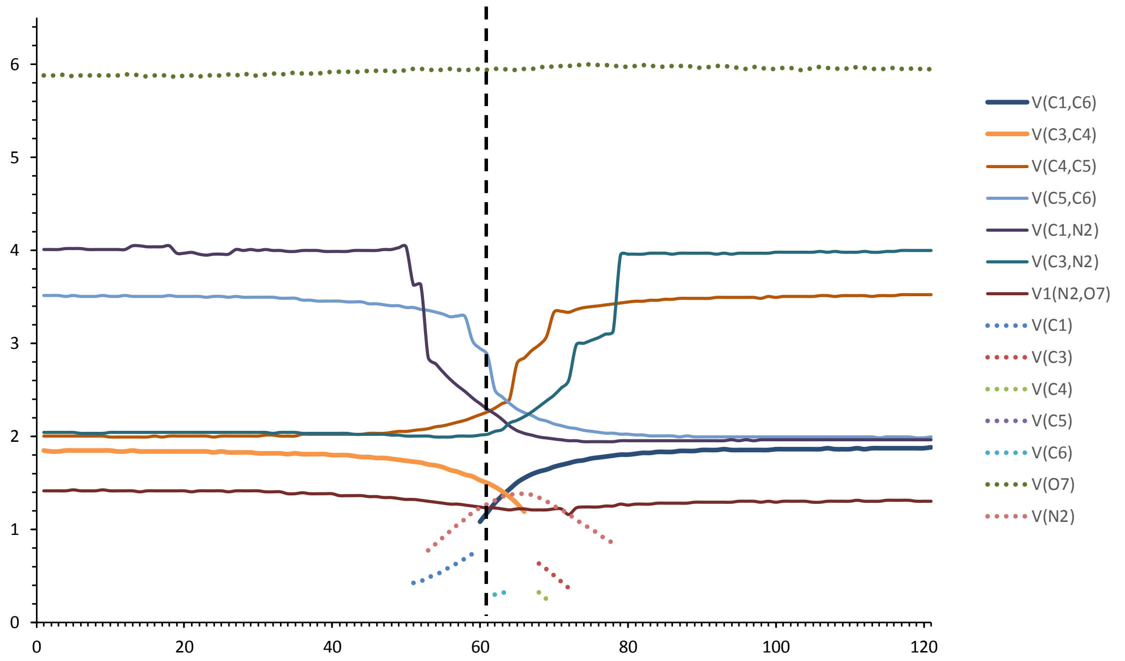


**Figura S7**. Graphical representation of ELF analysis for the rearrangement of nitrones **5**.

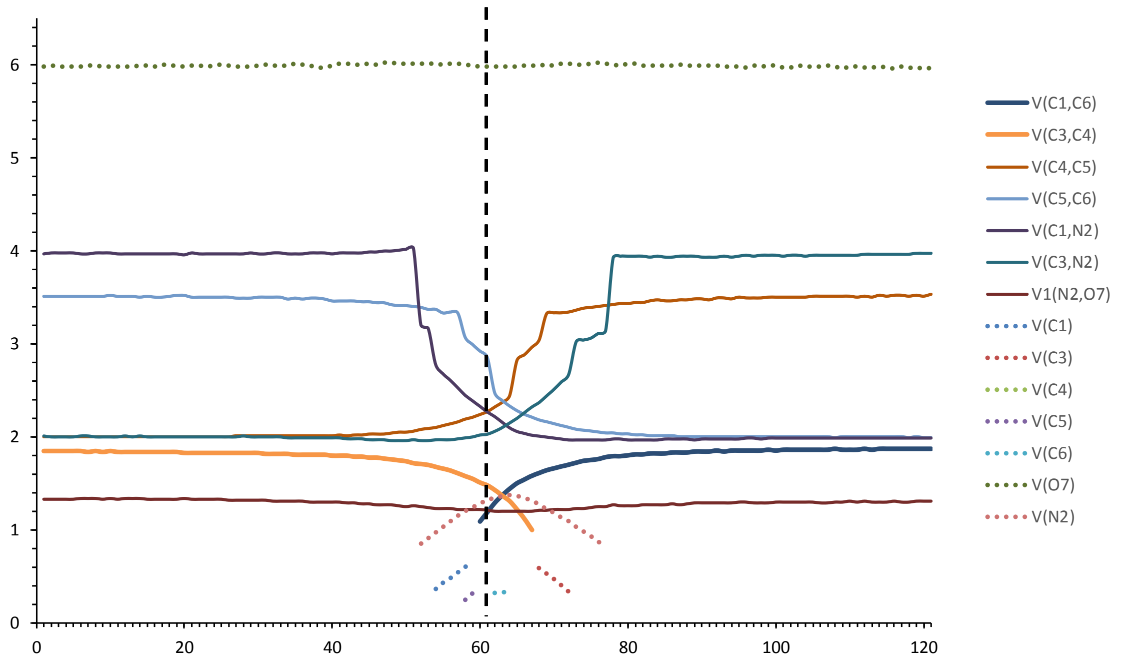


**Figura S8**. Graphical representation of ELF analysis for the rearrangement of nitrones **6**.

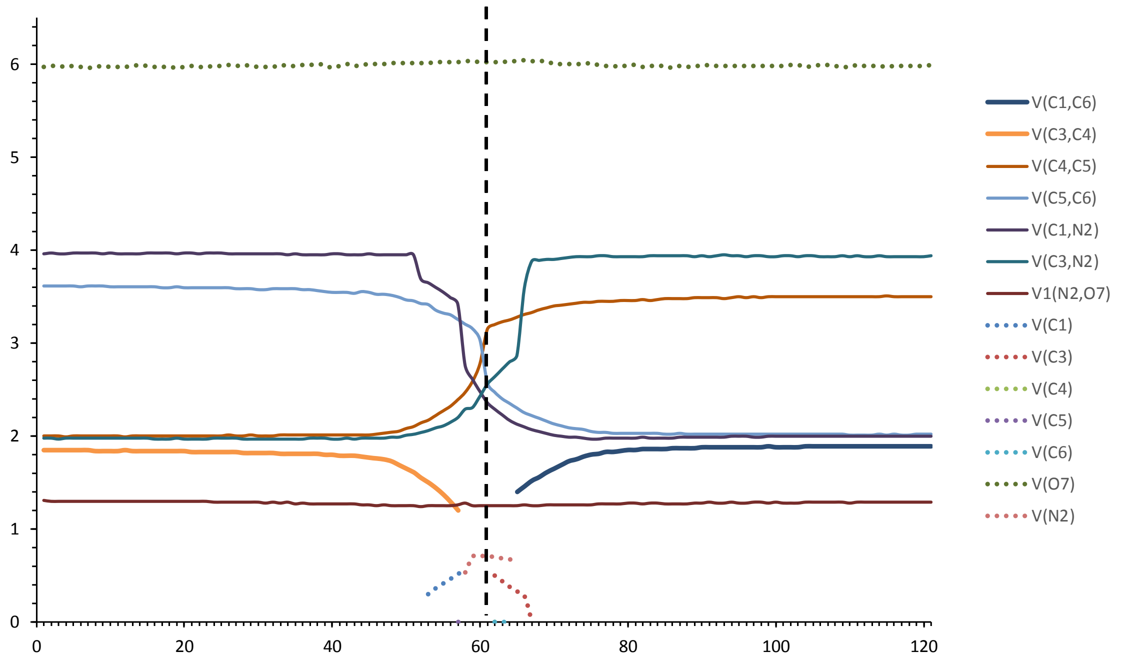


**Figura S9**. Graphical representation of ELF analysis for the rearrangement of nitrones (*Z*)-**7**.

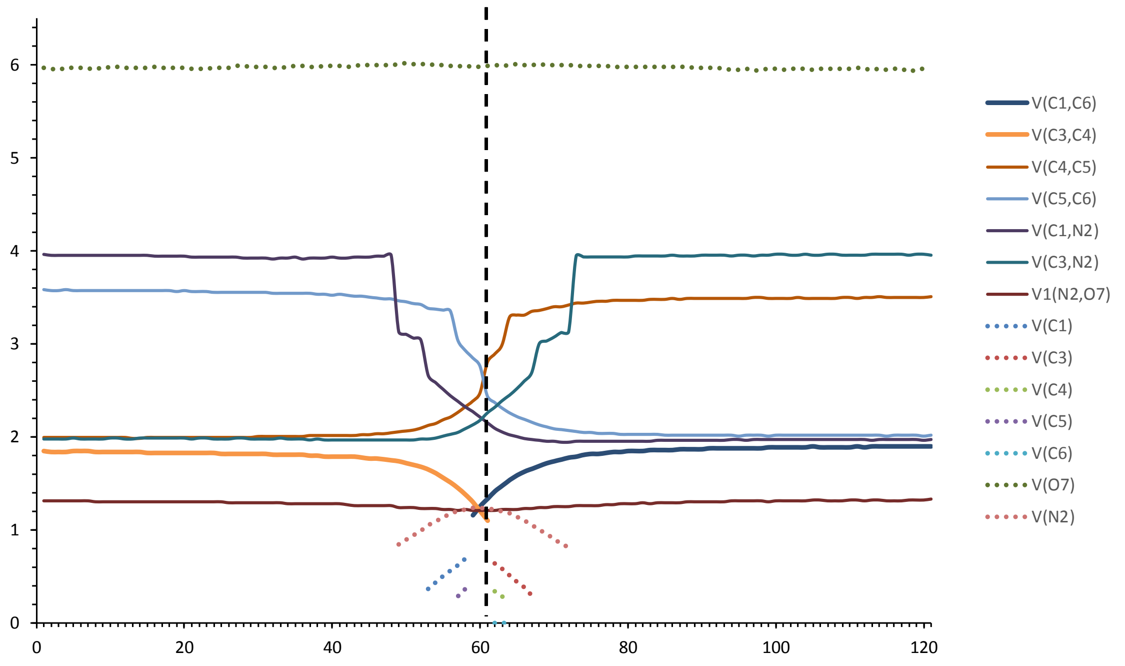


**Figura S10**. Graphical representation of ELF analysis for the rearrangement of nitrones (*E*)-**7**.

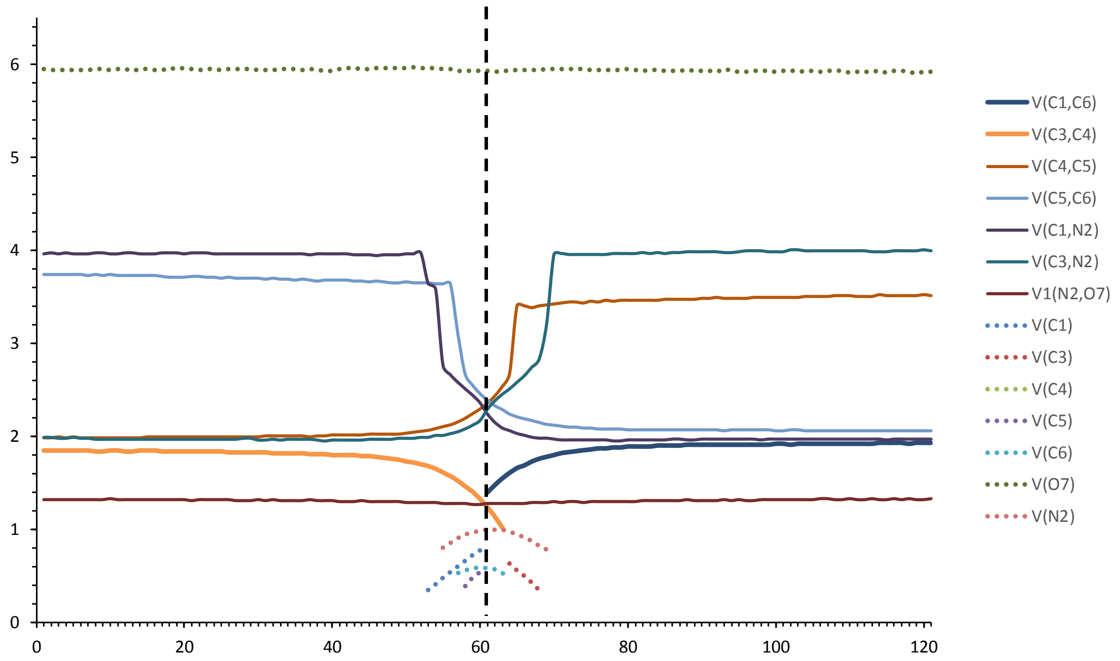


**Figura S11**. Graphical representation of ELF analysis for the rearrangement of nitrones (*Z*)-**8**.

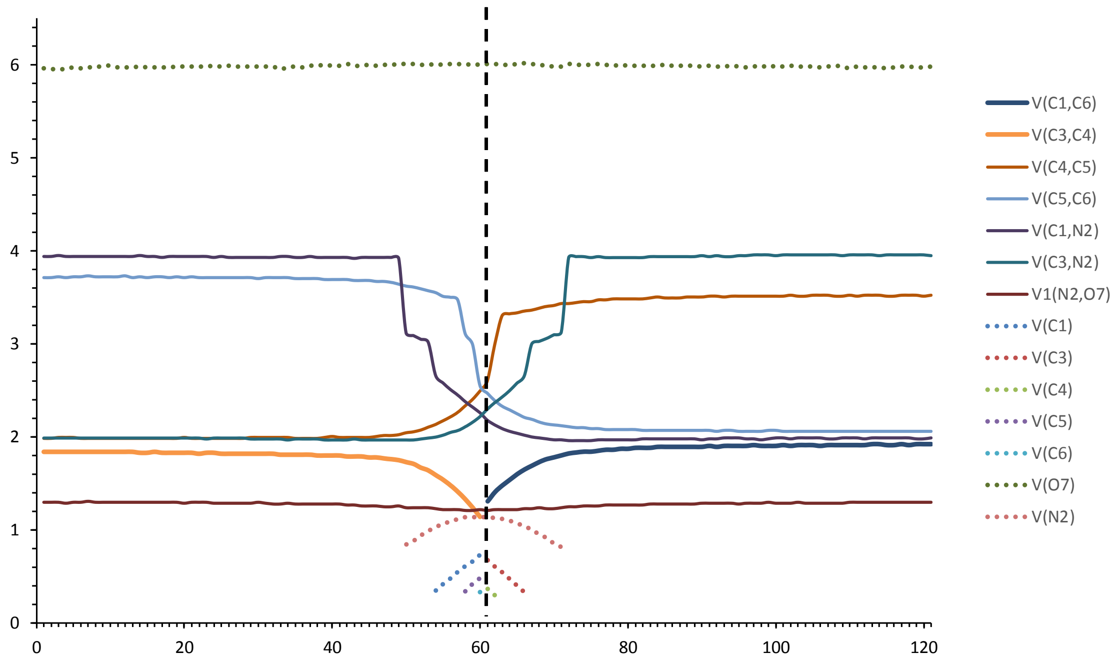


**Figura S12**. Graphical representation of ELF analysis for the rearrangement of nitrones (*E*)-**8**.

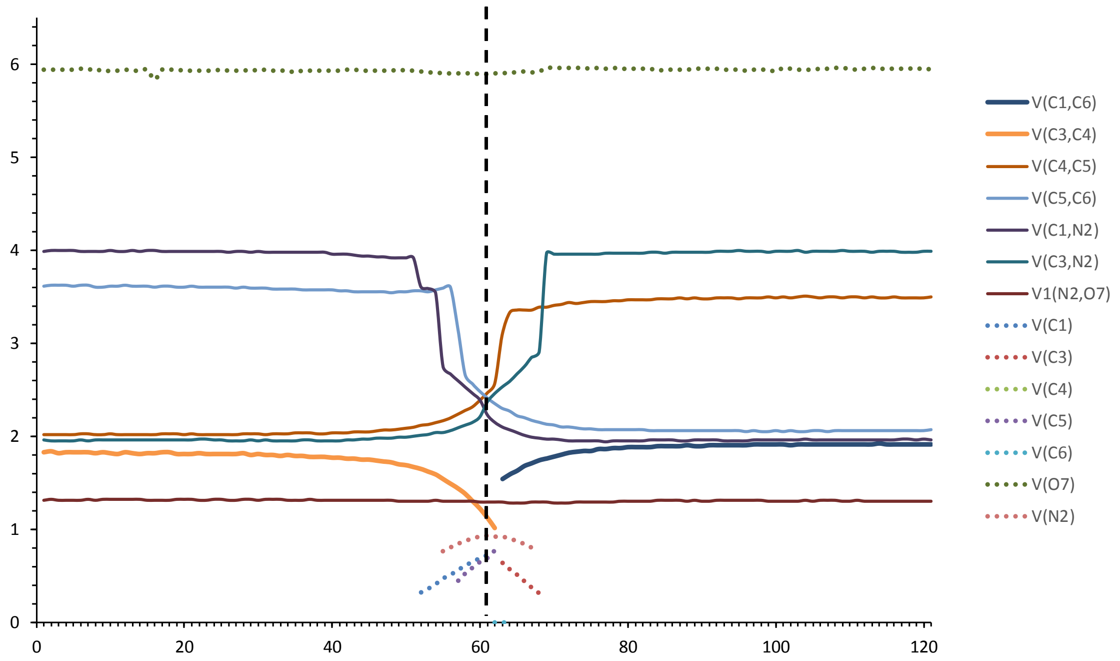


**Figura S13**. Graphical representation of ELF analysis for the rearrangement of nitrones (*Z*)-**9**.

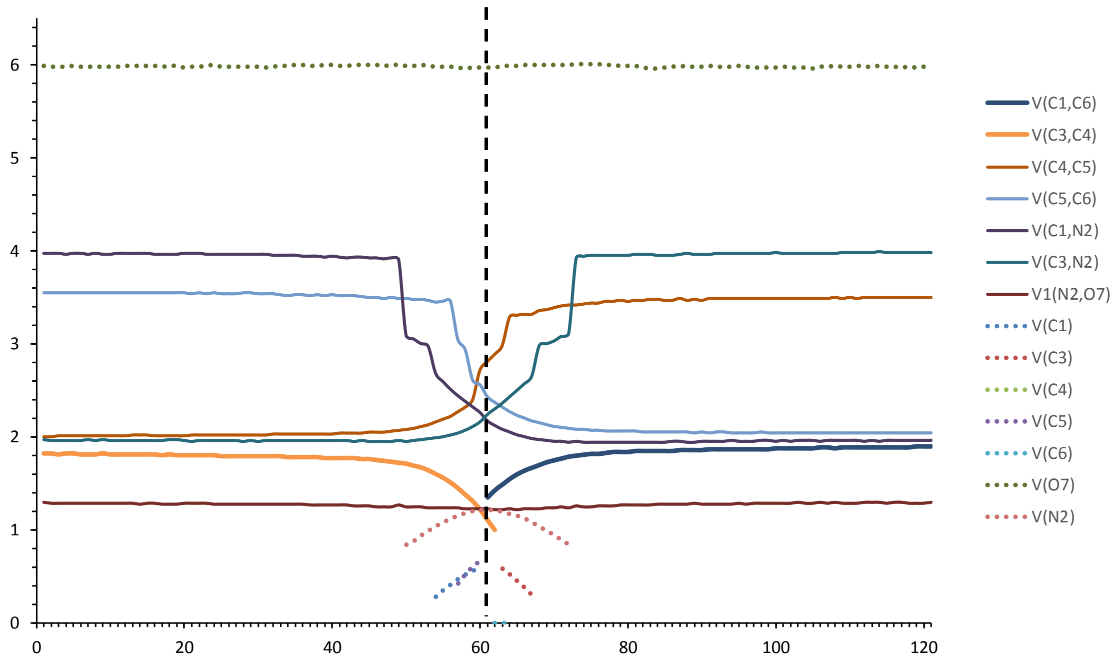


**Figura S14**. Graphical representation of ELF analysis for the rearrangement of nitrones (*E*)-**9**.

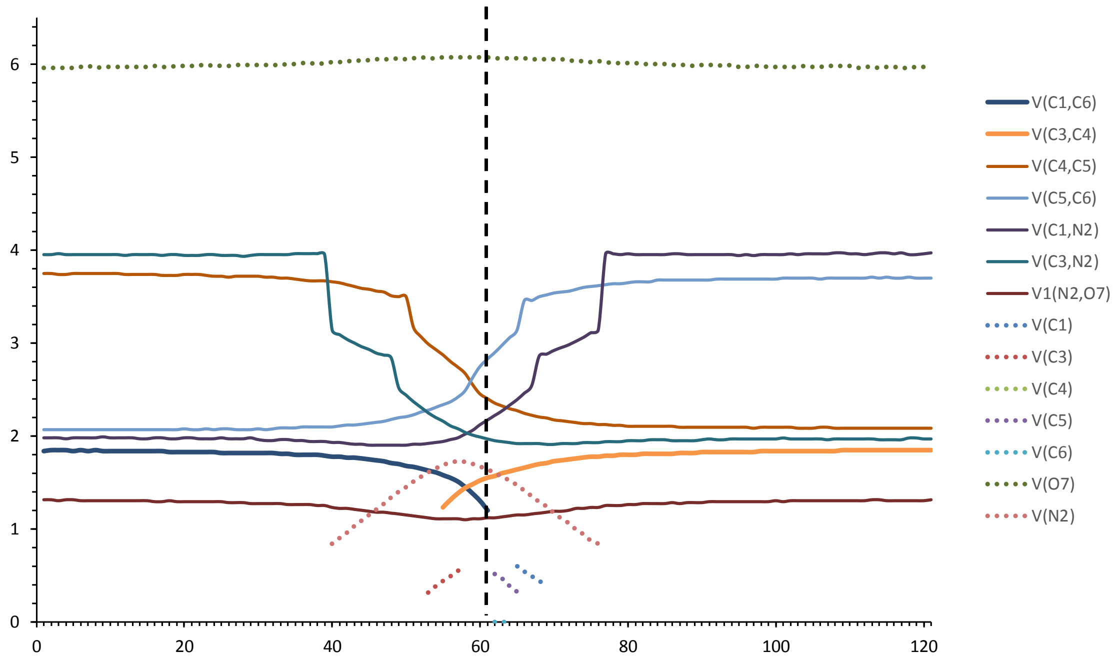


**Figura S15**. Graphical representation of ELF analysis for the rearrangement of nitrones **10**.

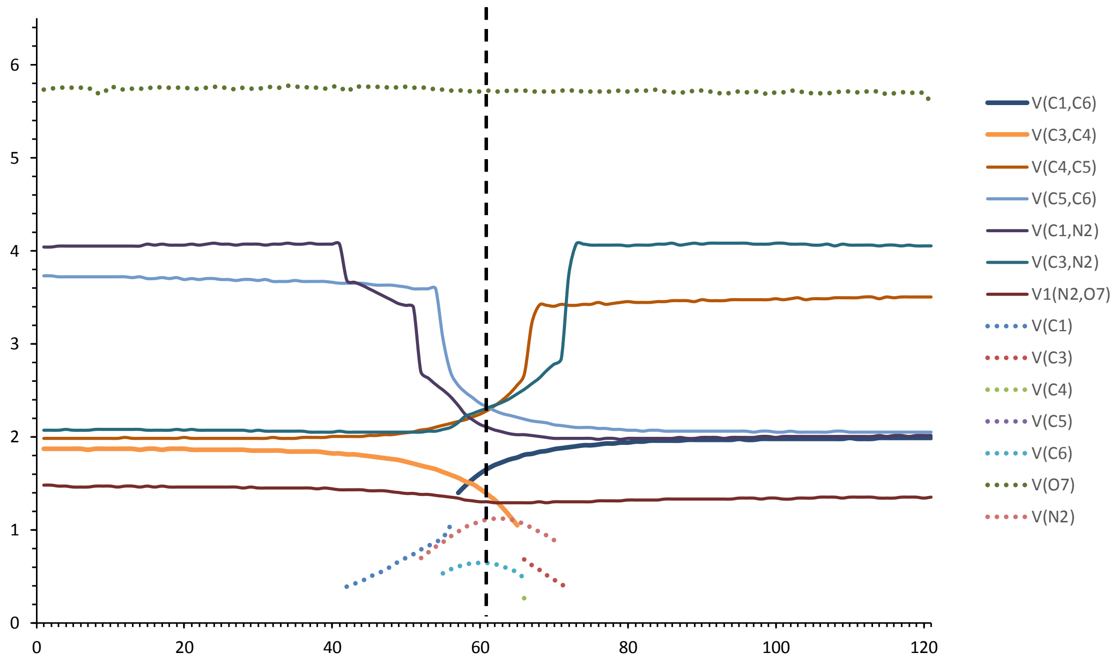


**Figura S16**. Graphical representation of ELF analysis for the rearrangement of nitrones (*Z*)-**11**.

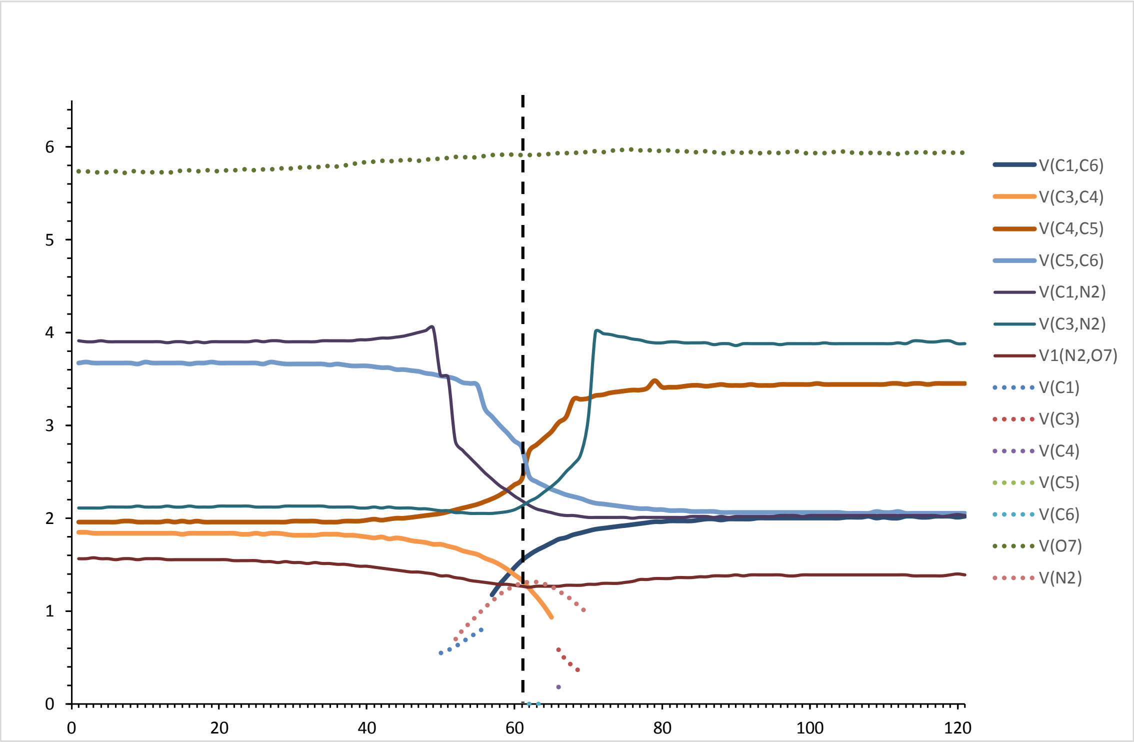


**Figura S11**. Graphical representation of ELF analysis for the rearrangement of nitrones (*E*)-**11**.

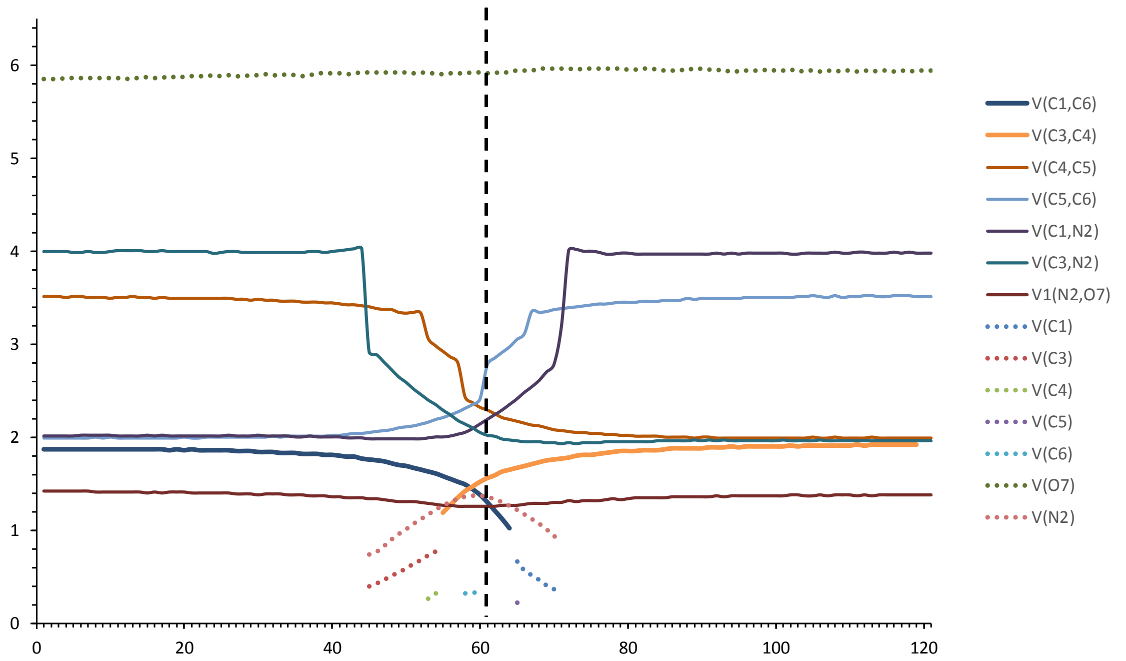


**Figura S12**. Graphical representation of ELF analysis for the rearrangement of nitrones **12**.

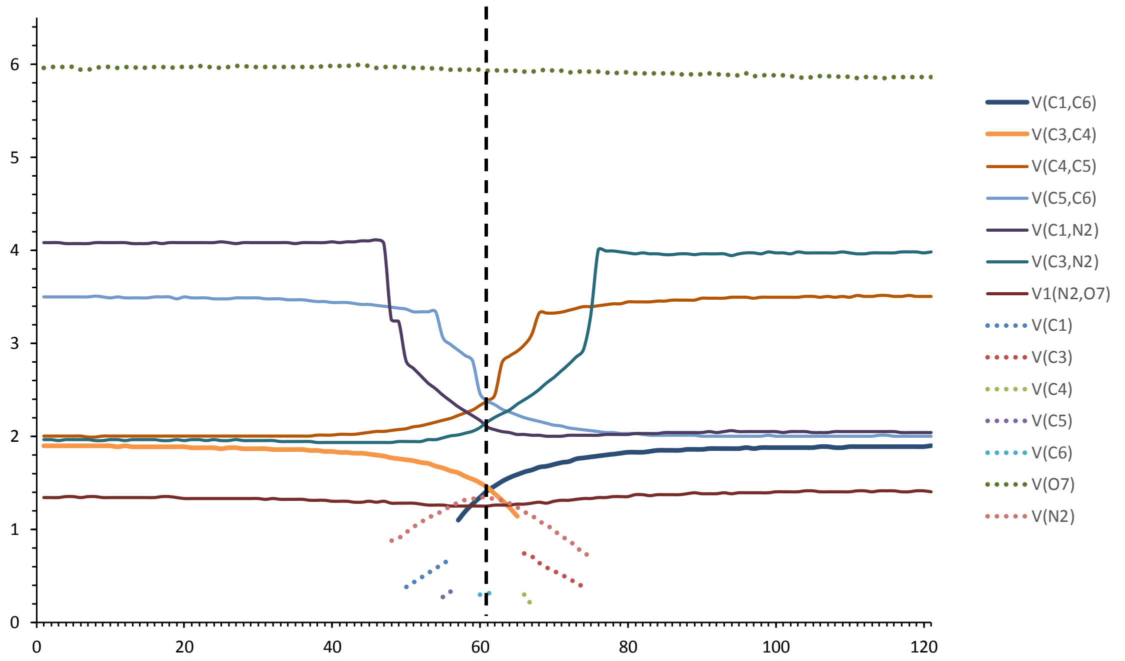


**Figura S13**. Graphical representation of ELF analysis for the rearrangement of nitrones **13**.

## Cartesian coordinates

01a

0 1

| | | | |
|---|---|---|---|
| C | -1.0571617692 | 0.2649938284 | 0.6536293730 |
| C | 1.1359111261 | 1.1268505433 | 0.0349377037 |
| N | -0.1479378570 | 0.3994031876 | -0.2881431447 |
| C | -0.5850592661 | 3.3817660465 | -2.0299047289 |
| C | 1.3989303515 | 2.1700763630 | -1.0730391898 |
| C | 2.2806283125 | 0.1462625679 | 0.2239718098 |
| O | -0.2429864998 | -0.0466568725 | -1.4838179974 |
| C | 2.6136564132 | -0.8020681142 | -0.7542140917 |
| C | 3.6934865792 | -1.6626313680 | -0.5578034011 |
| C | 4.4606269728 | -1.5836130965 | 0.6066974614 |
| C | 4.1366166376 | -0.6413127441 | 1.5828988178 |
| C | 3.0503378579 | 0.2140217963 | 1.3920315208 |
| H | -0.7973077991 | 0.7091430314 | 1.6068810912 |
| H | 0.9624987738 | 1.6478127585 | 0.9810209455 |
| H | 2.0053931006 | -0.8755702313 | -1.6487870591 |
| H | 3.9366457542 | -2.3986135474 | -1.3196972234 |
| H | 5.3034817682 | -2.2539263016 | 0.7527917067 |
| H | 4.7235251536 | -0.5733634272 | 2.4950784944 |
| H | 2.8006585623 | 0.9440109584 | 2.1595431200 |
| C | 0.3682988978 | 3.2680028586 | -1.1049956793 |
| H | -1.3128004591 | 4.1886853148 | -2.0042670422 |
| H | -0.6758625493 | 2.6627784767 | -2.8403603776 |
| H | 0.4163676785 | 3.9971973957 | -0.2939077482 |
| H | 1.4327966656 | 1.6443747420 | -2.0303949070 |
| H | 2.3960116795 | 2.5876212395 | -0.8844839596 |
| C | -2.3348515541 | -0.4118271262 | 0.5462503540 |
| C | -2.8103134225 | -1.0491975413 | -0.6199913430 |
| C | -3.1498350322 | -0.4243878673 | 1.6994900441 |
| C | -4.0565863441 | -1.6724696800 | -0.6146397487 |
| H | -2.1913120552 | -1.0425353642 | -1.5062285089 |
| C | -4.3913267882 | -1.0488452259 | 1.6937709194 |
| H | -2.7966384998 | 0.0632147015 | 2.6055917276 |
| C | -4.8515744954 | -1.6777106778 | 0.5331795761 |
| H | -4.4095946242 | -2.1598791430 | -1.5197871029 |
| H | -5.0011607789 | -1.0461840395 | 2.5932877061 |
| H | -5.8219034905 | -2.1669914422 | 0.5255798820 |

01b

0 1

| | | | |
|---|---|---|---|
| C | -1.1358577745 | 1.1268999115 | 0.0349679422 |
| C | 1.0572152755 | 0.2649652565 | 0.6535505073 |
| N | 0.1479471851 | 0.3994026065 | -0.2881754124 |
| C | -1.3988944538 | 2.1701305929 | -1.0730003204 |

| | | | |
|---|---|---|---|
| C | 0.5850889009 | 3.3817509728 | -2.0299671656 |
| C | 2.3348743739 | -0.4119036894 | 0.5461086986 |
| O | 0.2429169269 | -0.0466685318 | -1.4838523997 |
| C | 2.8102516470 | -1.0492986447 | -0.6201540782 |
| C | 4.0565017583 | -1.6726169260 | -0.6148639967 |
| C | 4.8515497173 | -1.6778806379 | 0.5329137920 |
| C | 4.3913859951 | -1.0489912524 | 1.6935254698 |
| C | 3.1499176860 | -0.4244878331 | 1.6993058475 |
| H | -0.9623772990 | 1.6478599491 | 0.9810398943 |
| H | 0.7974275969 | 0.7091299117 | 1.6068130890 |
| H | 2.1912042325 | -1.0426187983 | -1.5063589422 |
| H | 4.4094446335 | -2.1600447808 | -1.5200269512 |
| H | 5.8218601676 | -2.1671974336 | 0.5252662817 |
| H | 5.0012671171 | -1.0463473885 | 2.5930103547 |
| H | 2.7967866091 | 0.0631331818 | 2.6054231139 |
| C | -0.3682267460 | 3.2680214193 | -1.1050102006 |
| H | -2.3959521879 | 2.5877107582 | -0.8843984838 |
| H | -1.4328252102 | 1.6444265443 | -2.0303524627 |
| H | -0.4162297775 | 3.9972185890 | -0.2939207114 |
| H | 0.6758268583 | 2.6627597888 | -2.8404269538 |
| H | 1.3128589240 | 4.1886454333 | -2.0043668930 |
| C | -2.2806023494 | 0.1463560937 | 0.2240654693 |
| C | -2.6137149268 | -0.8019688290 | -0.7540972319 |
| C | -3.0502512108 | 0.2141524252 | 1.3921630522 |
| C | -3.6935677681 | -1.6624900108 | -0.5576267525 |
| H | -2.0054989054 | -0.8755000567 | -1.6486999197 |
| C | -4.1365527371 | -0.6411398092 | 1.5830903794 |
| H | -2.8005062317 | 0.9441374867 | 2.1596571856 |
| C | -4.4606472065 | -1.5834347626 | 0.6069117312 |
| H | -3.9367926076 | -2.3984683015 | -1.3195033759 |
| H | -4.7234132360 | -0.5731620374 | 2.4952988292 |
| H | -5.3035199766 | -2.2537151975 | 0.7530526138 |

02a

0 1

| | | | |
|---|---|---|---|
| C | 0.0591588249 | 1.4919810128 | -0.0662973881 |
| C | 2.0660174015 | 0.3074372094 | 0.6333279641 |
| N | 1.2191685870 | 0.5691809813 | -0.3391646765 |
| C | -0.0020710792 | 2.5542248177 | -1.1878476864 |
| C | 2.1925548682 | 3.4212470956 | -2.0506063210 |
| C | 3.2281893827 | -0.5566433445 | 0.5732066461 |
| O | 1.2822002619 | 0.1009275494 | -1.5306436846 |
| C | 3.6404545835 | -1.2687176665 | -0.5735930882 |
| C | 4.7766144495 | -2.0735648693 | -0.5217532523 |
| C | 5.5205639400 | -2.1888480129 | 0.6541863815 |
| C | 5.1217293440 | -1.4877467203 | 1.7959677578 |
| C | 3.9902259760 | -0.6821074334 | 1.7554857223 |
| H | 0.2744601496 | 1.9880631868 | 0.8843115713 |
| H | 1.8479044779 | 0.7983948055 | 1.5741633491 |

| | | | |
|---|---|---|---|
| H | 3.0612552916 | -1.1760740284 | -1.4815767442 |
| H | 5.0830439211 | -2.6167611521 | -1.4117843462 |
| H | 6.4047704772 | -2.8198287172 | 0.6828811932 |
| H | 5.6932478736 | -1.5704303909 | 2.7164023901 |
| H | 3.6837264380 | -0.1389302533 | 2.6467835301 |
| C | 1.1857502368 | 3.4805705916 | -1.1788589675 |
| H | -0.9304081984 | 3.1209577991 | -1.0429774250 |
| H | -0.0765643177 | 2.0270236946 | -2.1421140914 |
| H | 1.2066188592 | 4.2296554616 | -0.3853058253 |
| H | 2.2137556401 | 2.6755992873 | -2.8414641450 |
| H | 3.0332492505 | 4.1080567423 | -1.9990917282 |
| C | -1.2352684446 | 0.7094194177 | 0.0782016476 |
| C | -1.6219159499 | -0.2690826749 | -0.8514213821 |
| C | -2.0886918170 | 1.0070852392 | 1.1505965432 |
| C | -2.8335192128 | -0.9387496046 | -0.7075280561 |
| H | -0.9538399557 | -0.5138390764 | -1.6690647946 |
| C | -3.3079674978 | 0.3530885893 | 1.3014077059 |
| H | -1.7992164460 | 1.7612046922 | 1.8782392909 |
| C | -3.6623518900 | -0.6145750620 | 0.3649878378 |
| H | -3.1444703198 | -1.7014456601 | -1.4108497071 |
| H | -3.9763572742 | 0.5736035682 | 2.1244016916 |
| N | -4.9472469721 | -1.3152345525 | 0.5164242505 |
| O | -5.2384005000 | -2.1644864029 | -0.3251397316 |
| O | -5.6551953599 | -1.0097031195 | 1.4763515685 |

02b

0 1

| | | | |
|---|---|---|---|
| C | 0.0247027182 | 0.6282595516 | 0.5881206812 |
| C | 2.3325102465 | 1.1481924929 | -0.0128401076 |
| N | 0.9625720980 | 0.5919940945 | -0.3381967850 |
| C | 0.9991437497 | 3.5556040347 | -2.2121279232 |
| C | 2.7544395023 | 2.1014479088 | -1.1514506424 |
| C | 3.3198526613 | 0.0221175091 | 0.2386959511 |
| O | 0.8262828654 | 0.1119032006 | -1.5092639547 |
| C | 3.5639548251 | -0.9761455363 | -0.7155567432 |
| C | 4.5032912421 | -1.9749085839 | -0.4604165388 |
| C | 5.2166061400 | -1.9863408461 | 0.7403196015 |
| C | 4.9806542800 | -0.9949960563 | 1.6928614422 |
| C | 4.0341737083 | -0.0002519977 | 1.4430483227 |
| H | 0.3242152710 | 1.0767994467 | 1.5267959097 |
| H | 2.2136663453 | 1.7231912093 | 0.9098061539 |
| H | 2.9985211803 | -0.9786138072 | -1.6407449611 |
| H | 4.6786917682 | -2.7473692979 | -1.2043472521 |
| H | 5.9502705286 | -2.7645251656 | 0.9324682174 |
| H | 5.5275119179 | -0.9961749650 | 2.6318770247 |
| H | 3.8530771512 | 0.7685074821 | 2.1915711648 |
| C | 1.8867731660 | 3.3290014398 | -1.2435970489 |
| H | 0.3913914524 | 4.4563875493 | -2.2310053859 |
| H | 0.8459408286 | 2.8434858585 | -3.0194465923 |

| | | | |
|---|---|---|---|
| H | 2.0022016798 | 4.0608006688 | -0.4418093838 |
| H | 2.7339003368 | 1.5422998632 | -2.0902263664 |
| H | 3.7962094038 | 2.3838282137 | -0.9539393559 |
| C | -1.3280315468 | 0.1273778903 | 0.4719169595 |
| C | -1.8577004013 | -0.4974778220 | -0.6802510594 |
| C | -2.1650088575 | 0.2818875590 | 1.6020283569 |
| C | -3.1725378150 | -0.9476140405 | -0.6921891451 |
| H | -1.2251602043 | -0.6193118186 | -1.5477029072 |
| C | -3.4764962709 | -0.1645169813 | 1.5946601480 |
| H | -1.7721571517 | 0.7599856924 | 2.4955266677 |
| C | -3.9671626005 | -0.7777453333 | 0.4404855910 |
| H | -3.5918365360 | -1.4288020295 | -1.5672670320 |
| H | -4.1228392991 | -0.0498835548 | 2.4558423897 |
| N | -5.3544113280 | -1.2540084228 | 0.4221401302 |
| O | -5.7637227898 | -1.7911484638 | -0.6080276261 |
| O | -6.0303482659 | -1.0887959431 | 1.4394500987 |

03a

0 1

| | | | |
|---|---|---|---|
| C | -0.3328541066 | 0.5304410205 | 0.5675683169 |
| C | 1.9866496022 | 1.1329677301 | 0.1367078584 |
| N | 0.6410138223 | 0.6213143348 | -0.3123791192 |
| C | 0.7068831973 | 3.7676869391 | -1.7787837501 |
| C | 2.4575957298 | 2.2132551278 | -0.8620722766 |
| C | 2.9771553207 | -0.0082931048 | 0.2964204014 |
| O | 0.5573270248 | 0.2953481619 | -1.5508819800 |
| C | 3.2073654390 | -0.9398677125 | -0.7264801057 |
| C | 4.1509370339 | -1.9523792309 | -0.5536506677 |
| C | 4.8839604969 | -2.0442423564 | 0.6316697617 |
| C | 4.6620850087 | -1.1195762202 | 1.6523193838 |
| C | 3.7110400011 | -0.1118502040 | 1.4848101318 |
| H | -0.0757912998 | 0.8532533396 | 1.5694768669 |
| H | 1.8278482101 | 1.5965874476 | 1.1149497918 |
| H | 2.6234995678 | -0.8774028298 | -1.6381076260 |
| H | 4.3142520691 | -2.6730951847 | -1.3506963768 |
| H | 5.6209766318 | -2.8326851049 | 0.7593993205 |
| H | 5.2229354022 | -1.1837081104 | 2.5810970616 |
| H | 3.5400070497 | 0.6033153346 | 2.2871296404 |
| C | 1.5984951716 | 3.4502550933 | -0.8397534850 |
| H | 0.1023164705 | 4.6685806138 | -1.7116769995 |
| H | 0.5432743993 | 3.1283710877 | -2.6428234699 |
| H | 1.7199888666 | 4.1053236049 | 0.0252157205 |
| H | 2.4645387261 | 1.7655995449 | -1.8588222650 |
| H | 3.4928217487 | 2.4627060213 | -0.5976004653 |
| C | -1.6760224924 | 0.0438965213 | 0.3360773444 |
| C | -2.1648065578 | -0.4241562856 | -0.9069152828 |
| C | -2.5600731973 | 0.0374505980 | 1.4322672499 |
| C | -3.4708979024 | -0.8715256782 | -1.0264456800 |
| H | -1.4995163739 | -0.4256945202 | -1.7591412983 |

| | | | |
|---|---|---|---|
| C | -3.8725200085 | -0.4102824758 | 1.3179031346 |
| H | -2.2108670365 | 0.3916289846 | 2.3998295056 |
| C | -4.3369189896 | -0.8705014125 | 0.0779696790 |
| H | -3.8504930639 | -1.2319058345 | -1.9776556076 |
| H | -4.5176754627 | -0.3971114099 | 2.1888374369 |
| O | -5.5981928660 | -1.3316317608 | -0.1547866205 |
| C | -6.5188346302 | -1.3529893885 | 0.9246753758 |
| H | -7.4487178386 | -1.7532803951 | 0.5167785816 |
| H | -6.1711693131 | -2.0022157197 | 1.7391070509 |
| H | -6.7007638509 | -0.3448305660 | 1.3199854617 |

03b

0 1

| | | | |
|---|---|---|---|
| C | -0.3077337245 | 1.4697874346 | -0.0034622509 |
| C | 1.6905281984 | 0.2434080390 | 0.6663703309 |
| N | 0.8812086518 | 0.5880772337 | -0.3121268370 |
| C | -0.3188093597 | 2.6371929412 | -1.0147340870 |
| C | 1.8949162438 | 3.6099446094 | -1.7043658357 |
| C | 2.8644135247 | -0.6027474438 | 0.5746501226 |
| O | 0.9986995681 | 0.2328304887 | -1.5357142935 |
| C | 3.3345288697 | -1.1969268438 | -0.6162139753 |
| C | 4.4782063093 | -1.9928156927 | -0.5937998666 |
| C | 5.1743451399 | -2.2159775715 | 0.5957698805 |
| C | 4.7179149695 | -1.6327368798 | 1.7813871557 |
| C | 3.5786341169 | -0.8368543113 | 1.7701775235 |
| H | -0.1382360481 | 1.8702859748 | 1.0004772745 |
| H | 1.4273410987 | 0.6417403114 | 1.6387713385 |
| H | 2.7920806909 | -1.0214678972 | -1.5346986145 |
| H | 4.8283913323 | -2.4439421463 | -1.5186675976 |
| H | 6.0648801007 | -2.8388318156 | 0.6012185944 |
| H | 5.2510410156 | -1.7992089988 | 2.7136942846 |
| H | 3.2281476590 | -0.3853592313 | 2.6958773767 |
| C | 0.8620472116 | 3.5596415774 | -0.8628965346 |
| H | -1.2552991950 | 3.1859849578 | -0.8541050585 |
| H | -0.3486102269 | 2.2125433918 | -2.0211949208 |
| H | 0.8560957148 | 4.2087311700 | 0.0150338708 |
| H | 1.9452360179 | 2.9644578295 | -2.5778209320 |
| H | 2.7296460852 | 4.2877207003 | -1.5452288712 |
| C | -1.5895961406 | 0.6595589098 | 0.0088374087 |
| C | -1.9960010119 | -0.1158854949 | -1.0916438703 |
| C | -2.4201514650 | 0.6996659305 | 1.1303406872 |
| C | -3.1894049005 | -0.8228430414 | -1.0585652852 |
| H | -1.3508607646 | -0.1816416894 | -1.9605731204 |
| C | -3.6299387734 | 0.0005806087 | 1.1745202593 |
| H | -2.1280476263 | 1.2903865911 | 1.9964044817 |
| C | -4.0186985206 | -0.7678420755 | 0.0726206299 |
| H | -3.5046867301 | -1.4301415810 | -1.9013990565 |
| H | -4.2464454776 | 0.0594946194 | 2.0641344981 |
| O | -5.1713827914 | -1.4946561661 | -0.0011722191 |

| | | | |
|---|---|---|---|
| C | -6.0453806451 | -1.4817647294 | 1.1157835521 |
| H | -6.8880570976 | -2.1202256661 | 0.8443242796 |
| H | -5.5591709613 | -1.8857734960 | 2.0137793292 |
| H | -6.4124750585 | -0.4691675471 | 1.3302253480 |

04a

0 1

| | | | |
|---|---|---|---|
| C | -1.8896604017 | -0.9160970211 | 1.3333845845 |
| C | -0.3695405892 | 0.6408369609 | 0.2271149404 |
| N | -1.2854873509 | -0.5546737644 | 0.2331210296 |
| C | -3.2240974752 | 1.7617638171 | -1.4508796408 |
| C | -0.7681565792 | 1.5503135978 | -0.9562577932 |
| C | 1.0878164940 | 0.2138741912 | 0.1903380499 |
| O | -1.4273741159 | -1.1739833756 | -0.8799515994 |
| C | -2.7846715434 | -2.1049987622 | 1.3190659631 |
| C | 1.5711651831 | -0.6962983155 | -0.7610402900 |
| C | 2.9242804160 | -1.0343749442 | -0.7833497256 |
| C | 3.8133675596 | -0.4637036497 | 0.1301769763 |
| C | 3.3398937460 | 0.4448751401 | 1.0770110918 |
| C | 1.9844832747 | 0.7764217836 | 1.1074162008 |
| H | -1.7008183745 | -0.3256619658 | 2.2209645318 |
| H | -0.5555777784 | 1.1706147024 | 1.1666455232 |
| H | -2.2298352267 | -3.0045500074 | 1.0215949127 |
| H | -3.5795025558 | -1.9771564556 | 0.5729780320 |
| H | -3.2334615846 | -2.2679116983 | 2.3020621880 |
| H | 0.8742540872 | -1.1511456082 | -1.4562942371 |
| H | 3.2854249238 | -1.7472102748 | -1.5201316497 |
| H | 4.8674419013 | -0.7272826183 | 0.1053378149 |
| H | 4.0214649181 | 0.8921375465 | 1.7958066028 |
| H | 1.6210537837 | 1.4823251700 | 1.8517198239 |
| C | -2.1351502984 | 2.1618092484 | -0.7936368745 |
| H | -4.1919702740 | 2.2304196840 | -1.2917540871 |
| H | -3.1842731727 | 0.9427704227 | -2.1648963313 |
| H | -2.2165376217 | 2.9735043456 | -0.0680323422 |
| H | -0.7195809224 | 0.9492813255 | -1.8676791347 |
| H | -0.0050024227 | 2.3351255254 | -1.0287055602 |

04b

0 1

| | | | |
|---|---|---|---|
| C | -2.4245987515 | -0.0897237086 | 0.6050544528 |
| C | 0.0024028346 | 0.0599422254 | 0.7932213856 |
| N | -1.0336647323 | -0.4034053692 | 0.1256314312 |
| C | -3.1584222540 | 0.6970119748 | -0.4983574616 |
| C | -1.9423028301 | 2.4199598509 | -1.8664076076 |
| C | 1.4040607841 | -0.1477681728 | 0.4854013814 |
| O | -0.9837175975 | -1.1211709734 | -0.9313203075 |
| C | -3.1264108165 | -1.4018674938 | 0.9559456258 |

| | | | |
|---|---|---|---|
| C | 1.8812950232 | -0.8973047574 | -0.6113336144 |
| C | 3.2526769126 | -1.0376270383 | -0.8157035579 |
| C | 4.1723333402 | -0.4450097255 | 0.0517621505 |
| C | 3.7111023339 | 0.2998949366 | 1.1410878430 |
| C | 2.3455204965 | 0.4461720374 | 1.3543463207 |
| H | -2.3190701665 | 0.5328028136 | 1.4996363398 |
| H | -0.2439388511 | 0.6550961700 | 1.6641682182 |
| H | -2.5943836099 | -1.9283078304 | 1.7553421421 |
| H | -3.1622087991 | -2.0503609057 | 0.0772669507 |
| H | -4.1477600401 | -1.2004210155 | 1.2950748823 |
| H | 1.1654459036 | -1.3546929783 | -1.2800937355 |
| H | 3.6054989935 | -1.6180686284 | -1.6643329155 |
| H | 5.2394821314 | -0.5612236415 | -0.1176494793 |
| H | 4.4169672437 | 0.7661535002 | 1.8233674847 |
| H | 1.9923205518 | 1.0274204689 | 2.2035512568 |
| C | -2.5791732949 | 2.0626001710 | -0.7511004753 |
| H | -4.2086703695 | 0.7864891941 | -0.1875998692 |
| H | -3.1313252615 | 0.0928949039 | -1.4102976154 |
| H | -2.6908487728 | 2.7896752607 | 0.0558422786 |
| H | -1.8002234730 | 1.7212251690 | -2.6876644892 |
| H | -1.5349899285 | 3.4189125625 | -1.9981580159 |

05a

0 1

| | | | |
|---|---|---|---|
| C | -1.2940901898 | -0.3548626558 | 0.8435394325 |
| C | 0.6207701792 | 1.0256620922 | 0.2387933886 |
| N | -0.5126280271 | 0.0791718324 | -0.1129328534 |
| C | -1.7391763858 | 3.0751928027 | -1.3496389648 |
| C | 0.5436096379 | 2.2364766182 | -0.7154892192 |
| C | 1.9496707769 | 0.2942771627 | 0.2034919058 |
| O | -0.6290144227 | -0.2367906670 | -1.3299726899 |
| C | -2.4148354680 | -1.2986773127 | 0.5494398777 |
| C | 2.3952642435 | -0.3766539563 | -0.9445347439 |
| C | 3.6369056850 | -1.0115133664 | -0.9466987368 |
| C | 4.4518542465 | -0.9772327861 | 0.1870214847 |
| C | 4.0158568921 | -0.3088825444 | 1.3312016969 |
| C | 2.7693750950 | 0.3185733182 | 1.3387184879 |
| H | -1.1214291399 | -0.0354596464 | 1.8597366546 |
| H | 0.4253114910 | 1.3578094516 | 1.2622817510 |
| H | 1.7556188447 | -0.4214310097 | -1.8188935120 |
| H | 3.9682427412 | -1.5357238405 | -1.8389940776 |
| H | 5.4198925556 | -1.4707897452 | 0.1788603502 |
| H | 4.6403544962 | -0.2791915359 | 2.2199389927 |
| H | 2.4329381484 | 0.8341924315 | 2.2358680131 |
| C | -0.6990442449 | 3.0644345916 | -0.5157176628 |
| H | -2.6192451549 | 3.6827775806 | -1.1565108714 |
| H | -1.7499554548 | 2.4685001332 | -2.2518262623 |
| H | -0.7316454547 | 3.6729235967 | 0.3898349173 |
| H | 0.5999212162 | 1.8649964144 | -1.7418934544 |

| | | | |
|---|---|---|---|
| H | 1.4412079993 | 2.8392675397 | -0.5295362348 |
| F | -3.3198692824 | -0.7859630657 | -0.3060905869 |
| F | -3.0574517779 | -1.5706652476 | 1.7107303358 |
| F | -1.9925682458 | -2.4698091861 | 0.0346655813 |

05b

0 1

| | | | |
|---|---|---|---|
| C | -1.5327212451 | 0.5321030703 | 0.3511263351 |
| C | 0.8211003149 | 0.0826551934 | 0.7112231755 |
| N | -0.1824164877 | 0.1247360819 | -0.1415453912 |
| C | -2.0920910483 | 1.6693922746 | -0.5190051172 |
| C | -0.5023010955 | 3.4662057317 | -1.2539641910 |
| C | 2.1912723835 | -0.2854365180 | 0.4204072539 |
| O | -0.1301452643 | -0.1580468179 | -1.3872653890 |
| C | -2.4601375825 | -0.6883799355 | 0.3663546073 |
| C | 2.6609580771 | -0.6865534255 | -0.8489153942 |
| C | 4.0033069051 | -1.0182061717 | -1.0205420864 |
| C | 4.8988699383 | -0.9604171678 | 0.0490968149 |
| C | 4.4441341603 | -0.5658423747 | 1.3108600926 |
| C | 3.1075686495 | -0.2329302805 | 1.4938648682 |
| H | -1.4351398556 | 0.8476644895 | 1.3928724906 |
| H | 0.5714798345 | 0.3541308934 | 1.7294498718 |
| H | 1.9634772717 | -0.7320010754 | -1.6736285589 |
| H | 4.3520234202 | -1.3262646006 | -2.0026918687 |
| H | 5.9435667494 | -1.2214654217 | -0.0968624583 |
| H | 5.1322947205 | -0.5185460780 | 2.1504615538 |
| H | 2.7582274554 | 0.0732398325 | 2.4775581369 |
| C | -1.3287069145 | 2.9549215998 | -0.3418043455 |
| H | -3.1439600543 | 1.8079042005 | -0.2404751777 |
| H | -2.0558659238 | 1.3337012482 | -1.5577220939 |
| H | -1.4701961253 | 3.4744147803 | 0.6074706783 |
| H | -0.3275604420 | 2.9672228093 | -2.2041316084 |
| H | 0.0321625562 | 4.3965931143 | -1.0819963295 |
| F | -2.8220513088 | -1.0965457280 | -0.8560952753 |
| F | -3.5907952331 | -0.3659302952 | 1.0420046625 |
| F | -1.8875748557 | -1.7264124291 | 1.0066127438 |

06a

0 1

| | | | |
|---|---|---|---|
| C | -1.6575418153 | -0.2865983087 | 0.5924843746 |
| C | 0.4034235598 | 0.9537557326 | 0.1849120685 |
| N | -0.7617241979 | 0.1129381568 | -0.2637806849 |
| C | -1.6437418807 | 3.2273517710 | -1.5397439377 |
| C | 0.5004677954 | 2.1807181994 | -0.7481780309 |
| C | 1.6812092371 | 0.1340319736 | 0.2361079689 |
| O | -0.8201909524 | -0.1805308560 | -1.5123589250 |
| C | -2.7911532067 | -1.1228300982 | 0.1073796934 |

| | | | |
|---|---|---|---|
| C | 2.1136135023 | -0.6344192892 | -0.8545776283 |
| C | 3.3117693515 | -1.3446139659 | -0.7801909648 |
| C | 4.0982772155 | -1.2897727401 | 0.3726452208 |
| C | 3.6754147869 | -0.5249348807 | 1.4600119266 |
| C | 2.4716846620 | 0.1780544702 | 1.3913434251 |
| H | -1.5620168840 | -0.0131904008 | 1.6348893311 |
| H | 0.1595643353 | 1.2888453171 | 1.1978963121 |
| H | -2.4030084748 | -2.0426733196 | -0.3678224492 |
| H | -3.3392931812 | -0.5834758380 | -0.6868951017 |
| H | 1.4910448318 | -0.6895293953 | -1.7407262358 |
| H | 3.6323815781 | -1.9433760787 | -1.6287444440 |
| H | 5.0331152713 | -1.8416641749 | 0.4237880117 |
| H | 4.2766643364 | -0.4788790700 | 2.3641918789 |
| H | 2.1449084530 | 0.7680657947 | 2.2453461901 |
| C | -0.6800028016 | 3.1079723147 | -0.6261805320 |
| H | -2.4803981840 | 3.9075347194 | -1.4019447015 |
| H | -1.6320710684 | 2.6408420201 | -2.4552253739 |
| H | -0.7351507840 | 3.6995484608 | 0.2897151003 |
| H | 0.5989675226 | 1.8144166772 | -1.7731807366 |
| H | 1.4277148026 | 2.7067386566 | -0.4883847577 |
| C | -4.7310968857 | -2.2095721332 | 0.8560979793 |
| H | -5.3078049405 | -2.3843048391 | 1.7679783488 |
| H | -5.3729612594 | -1.7040679256 | 0.1167623786 |
| H | -4.4190964228 | -3.1797410885 | 0.4371030119 |
| O | -3.6208443019 | -1.4146238615 | 1.2130542833 |

06b

0 1

| | | | |
|---|---|---|---|
| C | -1.7499396468 | 0.3668046746 | 0.2007240548 |
| C | 0.6160112678 | 0.0187444703 | 0.6447762888 |
| N | -0.3865154484 | -0.0951973085 | -0.2016975615 |
| C | -2.1828182811 | 1.5470655688 | -0.6873233546 |
| C | -0.5843406717 | 3.4028549136 | -1.2607361387 |
| C | 1.9935738855 | -0.3751958670 | 0.4237781365 |
| O | -0.3191578399 | -0.5784988504 | -1.3864444882 |
| C | -2.7048783986 | -0.8277571270 | 0.0902923663 |
| C | 2.4805261107 | -0.9537425025 | -0.7682051560 |
| C | 3.8263334744 | -1.2989156851 | -0.8756792821 |
| C | 4.7100121092 | -1.0812356166 | 0.1832353519 |
| C | 4.2388665666 | -0.5082361386 | 1.3681757847 |
| C | 2.8986352492 | -0.1600403118 | 1.4862192419 |
| H | -1.7045841328 | 0.6755308334 | 1.2494460118 |
| H | 0.3585835838 | 0.4585210911 | 1.6005274411 |
| H | -2.2856711689 | -1.6911233954 | 0.6334173688 |
| H | -2.8204052401 | -1.1166174732 | -0.9633455488 |
| H | 1.7919327857 | -1.1198609322 | -1.5850149705 |
| H | 4.1876027102 | -1.7435450274 | -1.7994179598 |
| H | 5.7574437600 | -1.3547643186 | 0.0879796533 |
| H | 4.9173043085 | -0.3334437293 | 2.1989692784 |

| | | | |
|---|---|---|---|
| H | 2.5373685696 | 0.2860558591 | 2.4103538600 |
| C | -1.4238012910 | 2.8149722791 | -0.4078506161 |
| H | -3.2542097997 | 1.6954278381 | -0.5046355825 |
| H | -2.0552700279 | 1.2416872291 | -1.7309094806 |
| H | -1.5850192475 | 3.2630752165 | 0.5747650103 |
| H | -0.3905674449 | 2.9861045060 | -2.2466950250 |
| H | -0.0601480311 | 4.3205411034 | -1.0073096348 |
| C | -4.9331474313 | -1.4257383398 | 0.5553463489 |
| H | -5.8399054916 | -1.0218936916 | 1.0127732086 |
| H | -5.1430174915 | -1.6833708810 | -0.4945433932 |
| H | -4.6423149227 | -2.3457045491 | 1.0868300774 |
| O | -3.9370633737 | -0.4282058380 | 0.6576687088 |

07Ea

0 1

| | | | |
|---|---|---|---|
| C | -0.3310450086 | -1.1670057776 | 0.7924803786 |
| C | 1.3944600962 | 0.5245883275 | 0.4835863544 |
| N | 0.3620452330 | -0.4370304029 | -0.0551001269 |
| C | -1.1933924277 | 2.5957491595 | -0.6282733749 |
| C | 1.1779357478 | 1.9006439288 | -0.1844512607 |
| C | 2.7987885669 | -0.0235881122 | 0.2954195332 |
| O | 0.2462000482 | -0.4671705236 | -1.3295550114 |
| C | 3.2650900418 | -0.4618643346 | -0.9525741103 |
| C | 4.5742486614 | -0.9231104399 | -1.0896650226 |
| C | 5.4377605140 | -0.9432357418 | 0.0078845581 |
| C | 4.9817011249 | -0.5061571485 | 1.2516671090 |
| C | 3.6688752013 | -0.0540436595 | 1.3925857779 |
| H | -0.0937169017 | -1.0037594885 | 1.8367308470 |
| H | 1.1959678240 | 0.6153154444 | 1.5554502921 |
| H | 2.5879665252 | -0.4623208474 | -1.7994931414 |
| H | 4.9212540562 | -1.2676848507 | -2.0603309874 |
| H | 6.4582085186 | -1.2995088749 | -0.1055176490 |
| H | 5.6430978790 | -0.5212888710 | 2.1139548353 |
| H | 3.3182012327 | 0.2813489333 | 2.3666879542 |
| C | -0.1366938962 | 2.5370987333 | 0.1871049877 |
| H | -0.2007129006 | 2.9447173180 | 1.1963017718 |
| H | 1.2532371240 | 1.7637336199 | -1.2661491527 |
| H | 2.0149869167 | 2.5374218210 | 0.1289462320 |
| C | -1.3468619345 | -2.1513361072 | 0.4731183336 |
| C | -1.7516222553 | -2.4920709558 | -0.8356136572 |
| C | -1.9651211296 | -2.8071732975 | 1.5603662665 |
| C | -2.7390535750 | -3.4556923558 | -1.0312276110 |
| H | -1.2811232625 | -1.9943732756 | -1.6720021516 |
| C | -2.9493630821 | -3.7663492737 | 1.3538754918 |
| H | -1.6626539933 | -2.5555802462 | 2.5746324758 |
| C | -3.3418996889 | -4.0959397041 | 0.0532605450 |
| H | -3.0399048363 | -3.7085404992 | -2.0446652828 |
| H | -3.4109867399 | -4.2583363451 | 2.2058757320 |
| H | -4.1109753977 | -4.8458989377 | -0.1112945204 |

| | | | |
|---|---|---|---|
| H | -1.0913539078 | 2.1563441749 | -1.6227895371 |
| C | -3.6161407427 | 2.0764113504 | -0.5141348709 |
| H | -4.6283125282 | 2.4771271183 | -0.3768292656 |
| H | -3.5642436538 | 1.6279487531 | -1.5132505020 |
| H | -3.4616223166 | 1.2757233275 | 0.2176655072 |
| C | -2.5592070717 | 3.1933154627 | -0.3471287568 |
| C | -2.8276240743 | 4.2996969058 | -1.3940510553 |
| H | -2.1048934003 | 5.1178669522 | -1.2962284988 |
| H | -2.7526452045 | 3.9057623099 | -2.4147385036 |
| H | -3.8348654196 | 4.7160568244 | -1.2682095046 |
| C | -2.6688225323 | 3.7920646252 | 1.0646612068 |
| H | -3.6679245340 | 4.2154169081 | 1.2204732760 |
| H | -2.5060526022 | 3.0312764115 | 1.8369673851 |
| H | -1.9388182941 | 4.5952926615 | 1.2187277040 |

07Eb

0 1

| | | | |
|---|---|---|---|
| C | -0.7502819645 | 0.3288825044 | 0.3202878959 |
| C | 1.6173873679 | -0.1264270727 | 0.7132111397 |
| N | 0.6428055603 | -0.0269891728 | -0.1650830047 |
| C | -1.2156475012 | 1.5865724159 | -0.4853491894 |
| C | 0.6981116057 | 3.0433000901 | -1.2653466960 |
| C | 3.0034814743 | -0.4584972154 | 0.4464482680 |
| O | 0.7545618955 | -0.2124519380 | -1.4242770349 |
| C | 3.5305924631 | -0.7350123861 | -0.8333901731 |
| C | 4.8824626346 | -1.0398453068 | -0.9797798043 |
| C | 5.7337500363 | -1.0773192236 | 0.1262514788 |
| C | 5.2227944166 | -0.8054732994 | 1.3987658874 |
| C | 3.8760865655 | -0.5002497240 | 1.5560215819 |
| H | -0.6353109783 | 0.6144842098 | 1.3690062827 |
| H | 1.3297335926 | 0.0614870188 | 1.7403838302 |
| H | 2.8676325065 | -0.7052832781 | -1.6867237996 |
| H | 5.2740086962 | -1.2505817779 | -1.9716557445 |
| H | 6.7864031402 | -1.3161438447 | 0.0001576853 |
| H | 5.8753685542 | -0.8317047024 | 2.2674414797 |
| H | 3.4843142360 | -0.2893957987 | 2.5488495430 |
| C | -0.1556316304 | 2.6582770086 | -0.3172258634 |
| H | -0.0944048255 | 3.1252359449 | 0.6674875259 |
| H | 0.6964691394 | 2.5881378846 | -2.2521854228 |
| H | 1.4425271574 | 3.8141445064 | -1.0820752333 |
| C | -1.5975324897 | -0.9314580206 | 0.2604083064 |
| C | -1.8346486731 | -1.6105120460 | -0.9441890162 |
| C | -2.1266304496 | -1.4578338119 | 1.4460952064 |
| C | -2.5982550404 | -2.7768927481 | -0.9554771044 |
| H | -1.3990391292 | -1.2326374012 | -1.8613508832 |
| C | -2.8994921645 | -2.6203490714 | 1.4330176834 |
| H | -1.9364783269 | -0.9525872574 | 2.3908322594 |
| C | -3.1391146127 | -3.2827006046 | 0.2291442480 |
| H | -2.7714099666 | -3.2939246406 | -1.8956572802 |

| | | | |
|---|---|---|---|
| H | -3.3062399977 | -3.0087730419 | 2.3629468939 |
| H | -3.7378779907 | -4.1894852906 | 0.2137937392 |
| H | -1.2289183231 | 1.2935831511 | -1.5403381237 |
| C | -3.7730067516 | 1.2100765960 | -0.6418332552 |
| H | -4.7443223753 | 1.7044004863 | -0.5161808190 |
| H | -3.6495088102 | 0.9894587887 | -1.7088329073 |
| H | -3.8139590644 | 0.2610417169 | -0.1056322501 |
| C | -2.6521722651 | 2.1413314022 | -0.1312130474 |
| C | -2.8326334941 | 3.4970333633 | -0.8523687476 |
| H | -2.1359952664 | 4.2577790716 | -0.4887420016 |
| H | -2.6732828007 | 3.3947091371 | -1.9325618749 |
| H | -3.8526115504 | 3.8682742356 | -0.6969896153 |
| C | -2.8375167029 | 2.3599906080 | 1.3848238939 |
| H | -3.8086856163 | 2.8306549276 | 1.5789669968 |
| H | -2.8212422821 | 1.4152848270 | 1.9396367747 |
| H | -2.0691609988 | 3.0187757801 | 1.8064642916 |

07Za

0 1

| | | | |
|---|---|---|---|
| C | 0.6226821131 | -1.2248665729 | -0.9655108611 |
| C | -1.3004045315 | 0.2665594209 | -0.8618772089 |
| N | -0.1089545913 | -0.4135583934 | -0.2329955709 |
| C | 1.0349194424 | 3.0606995063 | -0.7859497034 |
| C | -1.2227224910 | 1.7847189283 | -0.5856489595 |
| C | -2.5978197383 | -0.3665101829 | -0.3883900925 |
| O | 0.0938737861 | -0.1479398956 | 1.0050097900 |
| C | -2.9188282921 | -0.4751332081 | 0.9724873843 |
| C | -4.1372021593 | -1.0297310855 | 1.3639493183 |
| C | -5.0545844784 | -1.4723259113 | 0.4082957520 |
| C | -4.7435276509 | -1.3649461143 | -0.9472212091 |
| C | -3.5204583305 | -0.8191768446 | -1.3399408786 |
| H | 0.3037693628 | -1.3235486026 | -1.9959601186 |
| H | -1.2078111323 | 0.1016882554 | -1.9393641350 |
| H | -2.1983478375 | -0.1496213219 | 1.7142117941 |
| H | -4.3707772677 | -1.1155078410 | 2.4219435371 |
| H | -6.0043288395 | -1.8997821526 | 0.7187005058 |
| H | -5.4477643249 | -1.7091822271 | -1.7000255732 |
| H | -3.2827403713 | -0.7418683391 | -2.3990799589 |
| C | -0.0422152378 | 2.4261687049 | -1.2690038665 |
| H | -0.0789498580 | 2.3286972330 | -2.3573105298 |
| H | -1.2349610289 | 1.9421359848 | 0.4899110248 |
| H | -2.1522895708 | 2.2110702659 | -0.9876421102 |
| C | 1.7830987414 | -1.9834179707 | -0.5408615634 |
| C | 2.2854468539 | -2.0142500565 | 0.7780623747 |
| C | 2.4428686410 | -2.7457129719 | -1.5298963040 |
| C | 3.4087126171 | -2.7827139080 | 1.0782551581 |
| H | 1.7806772382 | -1.4395949401 | 1.5418574563 |
| C | 3.5632852635 | -3.5070121620 | -1.2199812335 |
| H | 2.0656965356 | -2.7333895505 | -2.5501980323 |

| | | | |
|---|---|---|---|
| C | 4.0530262769 | -3.5282280647 | 0.0893238692 |
| H | 3.7834749151 | -2.7980463317 | 2.0983824346 |
| H | 4.0556057861 | -4.0846804415 | -1.9977144570 |
| H | 4.9291453656 | -4.1224970783 | 0.3346992754 |
| H | 1.7425261965 | 3.3937492236 | -1.5496408423 |
| C | 1.5282366884 | 3.4206236679 | 0.6083533168 |
| C | 0.5046742822 | 3.2499416215 | 1.7444342331 |
| H | 0.9365528223 | 3.6196561118 | 2.6827922342 |
| H | -0.4087494643 | 3.8256448384 | 1.5538683005 |
| H | 0.2430256893 | 2.1987974940 | 1.8903761349 |
| C | 2.7573576155 | 2.5275309076 | 0.9079486541 |
| H | 3.5226291651 | 2.6244210166 | 0.1280899002 |
| H | 3.2143189740 | 2.8151999694 | 1.8634760012 |
| H | 2.4587638515 | 1.4762466833 | 0.9693865242 |
| C | 1.9775309392 | 4.9008354146 | 0.5665519875 |
| H | 2.4151374672 | 5.1955783586 | 1.5280332957 |
| H | 2.7330571703 | 5.0692337658 | -0.2106941280 |
| H | 1.1302143962 | 5.5659417960 | 0.3616440800 |

07Zb

0 1

| | | | |
|---|---|---|---|
| C | 0.8157552744 | 0.1613081324 | -0.7768570661 |
| C | -1.5160963857 | -0.4939261766 | -0.6529870379 |
| N | -0.4398463433 | -0.0960999188 | -0.0039852390 |
| C | 1.3210239469 | 1.6587771820 | -0.7781485883 |
| C | -0.8475195310 | 2.7259067386 | -1.6521085967 |
| C | -2.8091387418 | -0.8212104708 | -0.0854627489 |
| O | -0.3719391209 | 0.0489129099 | 1.2710311542 |
| C | -3.1234031186 | -0.7767991040 | 1.2904334268 |
| C | -4.4045533788 | -1.1132895119 | 1.7234203659 |
| C | -5.3920403984 | -1.4964510239 | 0.8140499012 |
| C | -5.0928840622 | -1.5441305937 | -0.5508913770 |
| C | -3.8188851459 | -1.2109037299 | -0.9937981627 |
| H | 0.5327829472 | -0.0036336252 | -1.8199102278 |
| H | -1.4058771878 | -0.5657584735 | -1.7272918354 |
| H | -2.3564620270 | -0.4797610795 | 1.9914122905 |
| H | -4.6323194445 | -1.0747459124 | 2.7854639999 |
| H | -6.3875722770 | -1.7562208726 | 1.1638567968 |
| H | -5.8535783277 | -1.8407888003 | -1.2679275002 |
| H | -3.5912064237 | -1.2493342367 | -2.0568077258 |
| C | 0.4659317381 | 2.4952331049 | -1.7262040632 |
| H | 1.0077589793 | 2.9421733058 | -2.5605533427 |
| H | -1.4718702563 | 2.3401955431 | -0.8523126741 |
| H | -1.3482881486 | 3.3350974404 | -2.4008528142 |
| C | 1.8778516209 | -0.9008192259 | -0.4702613928 |
| C | 1.8215895115 | -1.7793991607 | 0.6184256400 |
| C | 2.9279003690 | -1.0448867134 | -1.3903652951 |
| C | 2.8013130030 | -2.7601472186 | 0.7875686362 |
| H | 1.0169483648 | -1.6843821769 | 1.3377582617 |

| | | | |
|---|---|---|---|
| C | 3.9105616746 | -2.0183572763 | -1.2162274163 |
| H | 2.9758031155 | -0.3954136591 | -2.2621561322 |
| C | 3.8517839973 | -2.8818315609 | -0.1212427522 |
| H | 2.7393145125 | -3.4306019431 | 1.6408055114 |
| H | 4.7142753436 | -2.1073775714 | -1.9424525180 |
| H | 4.6132606628 | -3.6447070052 | 0.0167994189 |
| H | 2.2844080864 | 1.5402105779 | -1.2898122692 |
| C | 1.7085194415 | 2.4390652856 | 0.5449413151 |
| C | 0.5069132639 | 3.0062132415 | 1.3264999866 |
| H | 0.8748675151 | 3.5201166541 | 2.2235091158 |
| H | -0.0481940332 | 3.7393961887 | 0.7333948347 |
| H | -0.1737600818 | 2.2142767308 | 1.6434487902 |
| C | 2.5531322891 | 1.5592024787 | 1.4855762646 |
| H | 3.4126807502 | 1.1169439225 | 0.9686857820 |
| H | 2.9401860596 | 2.1736246978 | 2.3081209083 |
| H | 1.9543099658 | 0.7542996484 | 1.9151482940 |
| C | 2.5826626899 | 3.6354736862 | 0.0955738811 |
| H | 2.8987877635 | 4.2174781326 | 0.9692718729 |
| H | 3.4894164251 | 3.3014076532 | -0.4256504350 |
| H | 2.0350331227 | 4.3119367862 | -0.5704302380 |

08Ea

0 1

| | | | |
|---|---|---|---|
| C | -0.6901376175 | -0.4940900058 | 0.9113557928 |
| C | 1.2802216805 | 0.8329704561 | 0.3725356218 |
| N | 0.0970358440 | -0.0150415764 | -0.0270099762 |
| C | -1.0003954696 | 2.9647363874 | -1.0519734845 |
| C | 1.2764474567 | 2.1125695475 | -0.4971321219 |
| C | 2.5728178103 | 0.0409815355 | 0.2804089001 |
| O | -0.0489429287 | -0.2049447927 | -1.2864082699 |
| C | 2.9366516715 | -0.6572930566 | -0.8802617332 |
| C | 4.1505683821 | -1.3418803804 | -0.9362379532 |
| C | 5.0215775011 | -1.3311420729 | 0.1555058807 |
| C | 4.6679758465 | -0.6360642155 | 1.3122026569 |
| C | 3.4491066968 | 0.0409967150 | 1.3731537331 |
| H | -0.4110068937 | -0.2251905225 | 1.9228376044 |
| H | 1.1137606967 | 1.1147231419 | 1.4160492372 |
| H | 2.2509443815 | -0.6796787678 | -1.7199910246 |
| H | 4.4169659788 | -1.8861498358 | -1.8385593749 |
| H | 5.9680075524 | -1.8630100808 | 0.1055244117 |
| H | 5.3356729268 | -0.6241237272 | 2.1696847246 |
| H | 3.1776400275 | 0.5775616930 | 2.2801455900 |
| C | 0.0781231057 | 2.9922927585 | -0.2628637334 |
| H | 0.0820390697 | 3.6604447797 | 0.5967065312 |
| H | 1.3353628700 | 1.7994030640 | -1.5427921269 |
| H | 2.2025422594 | 2.6533380537 | -0.2678503809 |
| C | -1.8626603842 | -1.3280252155 | 0.7305368057 |
| C | -2.3462209668 | -1.7756183709 | -0.5178401708 |
| C | -2.5614913629 | -1.7079247966 | 1.8974333070 |

| | | | |
|---|---|---|---|
| C | -3.4879769799 | -2.5724255132 | -0.5782381611 |
| H | -1.8139405446 | -1.4906622332 | -1.4145950634 |
| C | -3.6993620568 | -2.5026591182 | 1.8258057628 |
| H | -2.2004812475 | -1.3705928486 | 2.8665850515 |
| C | -4.1693962497 | -2.9395032447 | 0.5838527370 |
| H | -3.8477587018 | -2.9112486848 | -1.5464087250 |
| H | -4.2205671372 | -2.7824892838 | 2.7374273266 |
| H | -5.0585445909 | -3.5615152732 | 0.5246264902 |
| H | -1.0676994392 | 2.2907975289 | -1.9063648858 |
| O | -2.0740681145 | 3.7767977405 | -0.8440845837 |
| C | -3.2241843516 | 3.4644093503 | -1.6185859263 |
| H | -3.9646771737 | 4.2370122250 | -1.4015276361 |
| H | -2.9980713278 | 3.4746912887 | -2.6938677631 |
| H | -3.6352462193 | 2.4826423515 | -1.3484290703 |

08Eb

0 1

| | | | |
|---|---|---|---|
| C | -0.9324720220 | 0.5792054616 | 0.3225414352 |
| C | 1.3778435946 | -0.0420829858 | 0.7424912579 |
| N | 0.4120180394 | 0.0990152226 | -0.1404508438 |
| C | -1.3709718359 | 1.7503216678 | -0.5960271934 |
| C | 0.4358909227 | 3.2100867872 | -1.5718312420 |
| C | 2.7287810647 | -0.5056682788 | 0.4950362692 |
| O | 0.5078555236 | -0.1440360309 | -1.3957924501 |
| C | 3.2280329151 | -0.8886222240 | -0.7684640051 |
| C | 4.5470731869 | -1.3198703279 | -0.8948147764 |
| C | 5.3919216652 | -1.3800822678 | 0.2151814861 |
| C | 4.9083420739 | -1.0031189868 | 1.4714818163 |
| C | 3.5944035632 | -0.5718482642 | 1.6089815321 |
| H | -0.7994455576 | 0.9826014679 | 1.3298985510 |
| H | 1.1071317572 | 0.2129577395 | 1.7598450135 |
| H | 2.5701449879 | -0.8408436280 | -1.6248644448 |
| H | 4.9178685448 | -1.6123939729 | -1.8737758424 |
| H | 6.4188496859 | -1.7179320480 | 0.1045722412 |
| H | 5.5563198974 | -1.0462942104 | 2.3428251087 |
| H | 3.2229086153 | -0.2803618953 | 2.5890513928 |
| C | -0.3886785970 | 2.8964555579 | -0.5732393865 |
| H | -0.3930588173 | 3.4808527795 | 0.3481040337 |
| H | 0.4628701594 | 2.6263408112 | -2.4883728043 |
| H | 1.1299842538 | 4.0433475684 | -1.4995402062 |
| C | -1.9401644933 | -0.5561101503 | 0.3786000754 |
| C | -2.1207669132 | -1.4455079019 | -0.6898069844 |
| C | -2.7278936218 | -0.7052161690 | 1.5263956017 |
| C | -3.0681959457 | -2.4657503573 | -0.6005095407 |
| H | -1.4989767279 | -1.3465226717 | -1.5725882400 |
| C | -3.6801207406 | -1.7209153988 | 1.6109247733 |
| H | -2.6026408652 | -0.0142971314 | 2.3566074008 |
| C | -3.8519392554 | -2.6064467600 | 0.5462881103 |
| H | -3.1928945431 | -3.1540540463 | -1.4324660370 |

| | | | |
|---|---|---|---|
| H | -4.2842765196 | -1.8205983680 | 2.5088811323 |
| H | -4.5905091450 | -3.4012550784 | 0.6102030528 |
| H | -1.4735919609 | 1.3587063420 | -1.6159469617 |
| O | -2.6330469809 | 2.1590981626 | -0.0808600071 |
| C | -3.3846597312 | 2.9494148747 | -0.9848998474 |
| H | -4.3240394936 | 3.1926961737 | -0.4820457004 |
| H | -2.8643974103 | 3.8807897799 | -1.2498797563 |
| H | -3.6057132733 | 2.3967027573 | -1.9108030144 |

08Za

0 1

| | | | |
|---|---|---|---|
| C | 0.8577931607 | -0.6463450740 | -0.8930104996 |
| C | -1.2332286327 | 0.6034716243 | -0.8196982584 |
| N | 0.0348850902 | 0.0907020729 | -0.1772476569 |
| C | 0.7117067886 | 3.5793885645 | -0.5172287016 |
| C | -1.3338219897 | 2.1264000381 | -0.5828290050 |
| C | -2.4434476465 | -0.1650249967 | -0.3181046605 |
| O | 0.2089712418 | 0.4200630312 | 1.0467324568 |
| C | -2.7487240568 | -0.2559648628 | 1.0477945678 |
| C | -3.8892122009 | -0.9402479001 | 1.4674644310 |
| C | -4.7446919826 | -1.5314563877 | 0.5349665786 |
| C | -4.4491979183 | -1.4423014382 | -0.8253602668 |
| C | -3.3026229534 | -0.7666146254 | -1.2460127256 |
| H | 0.5471929986 | -0.8185474947 | -1.9162027455 |
| H | -1.1236936572 | 0.4182466673 | -1.8923538530 |
| H | -2.0744426143 | 0.1883718513 | 1.7711812969 |
| H | -4.1110898146 | -1.0103081835 | 2.5291513510 |
| H | -5.6346095486 | -2.0595196429 | 0.8671605086 |
| H | -5.1057561536 | -1.9012119674 | -1.5598993155 |
| H | -3.0760777885 | -0.7035552180 | -2.3085365818 |
| C | -0.1965676122 | 2.8845618989 | -1.2075665406 |
| H | -0.0849980109 | 2.8508366406 | -2.2899804913 |
| H | -1.3611335540 | 2.3132643616 | 0.4919118277 |
| H | -2.2977382413 | 2.4435350752 | -1.0024751448 |
| C | 2.1090951889 | -1.2344520746 | -0.4563845085 |
| C | 2.6232036839 | -1.1489793576 | 0.8556435101 |
| C | 2.8552194298 | -1.9416954080 | -1.4248915754 |
| C | 3.8405700280 | -1.7511886917 | 1.1686044463 |
| H | 2.0526092728 | -0.6185161719 | 1.6050557120 |
| C | 4.0688931261 | -2.5369610168 | -1.1024393594 |
| H | 2.4705233832 | -2.0186097258 | -2.4396339181 |
| C | 4.5693215027 | -2.4432349605 | 0.1995701336 |
| H | 4.2224486521 | -1.6789114441 | 2.1837678568 |
| H | 4.6256277085 | -3.0749246280 | -1.8651354067 |
| H | 5.5179848388 | -2.9078360392 | 0.4547630745 |
| H | 1.5606793521 | 4.0689271373 | -1.0026072757 |
| O | 0.6200030067 | 3.7811854941 | 0.8277506521 |
| C | 1.8324508713 | 3.5530300345 | 1.5399225356 |
| H | 2.6665072697 | 4.1174366031 | 1.0962971176 |

| | | | |
|---|---|---|---|
| H | 1.6658334947 | 3.9015351934 | 2.5615041788 |
| H | 2.0732572869 | 2.4839150217 | 1.5481412549 |

08Zb

0 1

| | | | |
|---|---|---|---|
| C | 0.9278880802 | 0.4819048886 | -0.7529847733 |
| C | -1.3415739497 | -0.3793406996 | -0.7938581406 |
| N | -0.3615929535 | 0.1236144142 | -0.0678011225 |
| C | 1.2599175190 | 1.9939171512 | -0.5698353311 |
| C | -0.6989951684 | 3.5106700671 | -0.1315431918 |
| C | -2.6489461793 | -0.7922632742 | -0.3236271811 |
| O | -0.4081368129 | 0.3399190488 | 1.1883896793 |
| C | -3.0861633536 | -0.7053224284 | 1.0159562910 |
| C | -4.3669448901 | -1.1332596640 | 1.3603491788 |
| C | -5.2348325120 | -1.6511082410 | 0.3971319517 |
| C | -4.8131465754 | -1.7414892236 | -0.9327433988 |
| C | -3.5381834224 | -1.3174125080 | -1.2873544490 |
| H | 0.7434318454 | 0.3542397913 | -1.8232647998 |
| H | -1.1239089281 | -0.4956816135 | -1.8481622372 |
| H | -2.4110452159 | -0.3042896925 | 1.7587578624 |
| H | -4.6892164030 | -1.0605573244 | 2.3959567034 |
| H | -6.2312419545 | -1.9818285387 | 0.6779379261 |
| H | -5.4791227837 | -2.1431045151 | -1.6919128995 |
| H | -3.2156046363 | -1.3909037185 | -2.3238228977 |
| C | 0.0969832483 | 2.8726500834 | -0.9877409966 |
| H | -0.0741502401 | 2.9480977262 | -2.0623398213 |
| H | -0.5457441335 | 3.4258399980 | 0.9398474494 |
| H | -1.5280618341 | 4.1227864931 | -0.4752578758 |
| C | 2.0534294966 | -0.4538351842 | -0.3543131001 |
| C | 2.4192349588 | -0.6608977479 | 0.9840918138 |
| C | 2.7640879167 | -1.1158839233 | -1.3638630948 |
| C | 3.4721918798 | -1.5215428708 | 1.2931159230 |
| H | 1.8641743260 | -0.1570401948 | 1.7641445737 |
| C | 3.8251312731 | -1.9673471016 | -1.0521897594 |
| H | 2.4864677608 | -0.9661566522 | -2.4056700953 |
| C | 4.1804131276 | -2.1733097567 | 0.2806881578 |
| H | 3.7431027214 | -1.6812851109 | 2.3335675329 |
| H | 4.3652559259 | -2.4721072053 | -1.8488389369 |
| H | 5.0024597623 | -2.8392956560 | 0.5299469305 |
| H | 2.0947962992 | 2.1631473759 | -1.2742591044 |
| O | 1.7059226958 | 2.2469249483 | 0.7430929741 |
| C | 2.5791163877 | 3.3586187469 | 0.8409109226 |
| H | 2.0939563537 | 4.2910328625 | 0.5212789709 |
| H | 2.8594939541 | 3.4434931383 | 1.8935727596 |
| H | 3.4888974139 | 3.2065941116 | 0.2391396056 |

09Ea

0 1

| | | | |
|---|---|---|---|
| C | -0.5643909830 | -0.5857266428 | 0.8315752185 |
| C | 1.3776584272 | 0.7735557541 | 0.2787598019 |
| N | 0.2135818664 | -0.1037366728 | -0.1125113376 |
| C | -0.8720463280 | 2.7595826364 | -1.2978249880 |
| C | 1.4223870555 | 1.9779666326 | -0.6916985140 |
| C | 2.6776439631 | -0.0123573434 | 0.2939336992 |
| O | 0.0871290800 | -0.3246864344 | -1.3716444025 |
| C | 3.0607110140 | -0.8297268640 | -0.7789883762 |
| C | 4.2771132842 | -1.5109722461 | -0.7392649647 |
| C | 5.1283506303 | -1.3777255621 | 0.3599650026 |
| C | 4.7533811424 | -0.5639571849 | 1.4292839111 |
| C | 3.5322505371 | 0.1108024583 | 1.3964316010 |
| H | -0.2980775656 | -0.2862124021 | 1.8384803133 |
| H | 1.1688061239 | 1.1320494532 | 1.2906048727 |
| H | 2.3903192589 | -0.9444741836 | -1.6241342043 |
| H | 4.5605300172 | -2.1486687829 | -1.5722707027 |
| H | 6.0765870242 | -1.9078141157 | 0.3839982749 |
| H | 5.4055922022 | -0.4576638484 | 2.2918827346 |
| H | 3.2429474800 | 0.7396887616 | 2.2360772423 |
| C | 0.2273052966 | 2.8784681168 | -0.5542983637 |
| H | 0.2422927207 | 3.6562276265 | 0.2059830550 |
| H | 1.4999070499 | 1.5811886797 | -1.7057927792 |
| H | 2.3366911808 | 2.5382210059 | -0.4701360422 |
| C | -1.7053045203 | -1.4639172837 | 0.6648002330 |
| C | -2.1617142698 | -1.9606077717 | -0.5750333957 |
| C | -2.3984950383 | -1.8353076394 | 1.8377708956 |
| C | -3.2742747269 | -2.7978446071 | -0.6216073049 |
| H | -1.6349134813 | -1.6810554101 | -1.4765886132 |
| C | -3.5072268321 | -2.6707761975 | 1.7795104650 |
| H | -2.0574374751 | -1.4591065733 | 2.7997482904 |
| C | -3.9509346171 | -3.1562433437 | 0.5459720001 |
| H | -3.6163289164 | -3.1727456715 | -1.5825338495 |
| H | -4.0268659787 | -2.9432176664 | 2.6939922321 |
| H | -4.8186023674 | -3.8085205841 | 0.4970410054 |
| H | -1.0472666293 | 2.0213943628 | -2.0685816965 |
| N | -2.0055003923 | 3.6584545320 | -1.1040188098 |
| O | -2.9639892612 | 3.4835923811 | -1.8581893678 |
| O | -1.9459229718 | 4.5206606308 | -0.2251481364 |

09Eb

0 1

| | | | |
|---|---|---|---|
| C | -0.7802878032 | 0.5524762798 | 0.2195390831 |
| C | 1.5206712896 | -0.0435375072 | 0.7081380575 |
| N | 0.5735577121 | 0.0548250879 | -0.1996131957 |
| C | -1.1835810516 | 1.6443017960 | -0.8097057938 |
| C | 0.6087235513 | 3.0895551403 | -1.7693509459 |
| C | 2.8724864875 | -0.5273671484 | 0.5115195762 |
| O | 0.6873697374 | -0.2480804400 | -1.4414653354 |

| | | | |
|---|---|---|---|
| C | 3.3899241915 | -0.9859550105 | -0.7190198482 |
| C | 4.7072263183 | -1.4331630563 | -0.7961781799 |
| C | 5.5311354896 | -1.4341931619 | 0.3311653150 |
| C | 5.0292092221 | -0.9814884228 | 1.5548655195 |
| C | 3.7166139396 | -0.5341084013 | 1.6436535684 |
| H | -0.6650757548 | 1.0345311464 | 1.1916358462 |
| H | 1.2307643707 | 0.2649316928 | 1.7053920580 |
| H | 2.7479396767 | -0.9839576303 | -1.5887527621 |
| H | 5.0931825296 | -1.7848757698 | -1.7492880861 |
| H | 6.5570102463 | -1.7851046948 | 0.2589712623 |
| H | 5.6616031355 | -0.9787011640 | 2.4384352864 |
| H | 3.3300468399 | -0.1842926825 | 2.5983397109 |
| C | -0.2676195621 | 2.8378648610 | -0.7984285087 |
| H | -0.3448092121 | 3.4811160220 | 0.0759725701 |
| H | 0.7063665806 | 2.4389235612 | -2.6337454667 |
| H | 1.2752291832 | 3.9458067804 | -1.7166323362 |
| C | -1.7783275624 | -0.5866572769 | 0.3404646530 |
| C | -1.9588239860 | -1.5273968047 | -0.6839878522 |
| C | -2.5551918389 | -0.6821397190 | 1.5021352451 |
| C | -2.8967001621 | -2.5485287366 | -0.5366404755 |
| H | -1.3450967455 | -1.4676383922 | -1.5759382156 |
| C | -3.4985434865 | -1.7004495399 | 1.6420130894 |
| H | -2.4314888681 | 0.0507126917 | 2.2953339278 |
| C | -3.6700889137 | -2.6371779971 | 0.6227587555 |
| H | -3.0245047152 | -3.2763834744 | -1.3332487865 |
| H | -4.0966149973 | -1.7598140176 | 2.5471223320 |
| H | -4.4035048907 | -3.4317829216 | 0.7300847421 |
| H | -1.2694226526 | 1.2030997299 | -1.7994462095 |
| N | -2.5838288373 | 2.1133215642 | -0.4421813963 |
| O | -3.4591240367 | 2.0058925535 | -1.2887167703 |
| O | -2.7303784246 | 2.5828930627 | 0.6865315661 |

09Za

0 1

| | | | |
|---|---|---|---|
| C | -0.8389330965 | -0.6969107488 | 0.7216228486 |
| C | 1.0611815213 | 0.7075724563 | 0.1585985731 |
| N | -0.1651387961 | -0.0811662487 | -0.2251761440 |
| C | -0.4830236776 | 3.7447549004 | 0.2465429484 |
| C | 1.1301787248 | 1.9843262468 | -0.7101735121 |
| C | 2.3161657310 | -0.1376886880 | 0.0331759504 |
| O | -0.4410580428 | -0.1045822992 | -1.4796479646 |
| C | 2.5349507899 | -0.9720596807 | -1.0716240710 |
| C | 3.7124454821 | -1.7136345639 | -1.1652243122 |
| C | 4.6862754136 | -1.6225821836 | -0.1680228032 |
| C | 4.4752839750 | -0.7886982726 | 0.9306378870 |
| C | 3.2936371668 | -0.0531722975 | 1.0322027418 |
| H | -0.4488153140 | -0.5534889449 | 1.7225791102 |
| H | 0.9295168336 | 1.0133482724 | 1.1977231489 |
| H | 1.7721543841 | -1.0461748046 | -1.8405462798 |

| | | | |
|---|---|---|---|
| H | 3.8695356582 | -2.3642613151 | -2.0214153691 |
| H | 5.6037182707 | -2.1996616235 | -0.2466241012 |
| H | 5.2260844887 | -0.7130622197 | 1.7125720671 |
| H | 3.1321456872 | 0.5966985914 | 1.8893592992 |
| C | -0.1261557554 | 2.8047011240 | -0.6361984066 |
| H | -1.4054763589 | 4.3071967902 | 0.1918256715 |
| H | -0.8670617602 | 2.5908613183 | -1.4031376457 |
| H | 1.2924969484 | 1.6844288082 | -1.7471061418 |
| H | 1.9918723879 | 2.5528347088 | -0.3567792446 |
| C | -2.0080475731 | -1.5382116231 | 0.5603241840 |
| C | -2.6122967285 | -1.8430998276 | -0.6784599517 |
| C | -2.5689546607 | -2.0823805254 | 1.7366154329 |
| C | -3.7367499762 | -2.6647588394 | -0.7206387077 |
| H | -2.1858654227 | -1.4313028289 | -1.5825480817 |
| C | -3.6910273813 | -2.9004163829 | 1.6827955484 |
| H | -2.1125233930 | -1.8561618304 | 2.6977115004 |
| C | -4.2814310485 | -3.1954535578 | 0.4504608938 |
| H | -4.1916610897 | -2.8928745063 | -1.6809874485 |
| H | -4.1059424148 | -3.3088221546 | 2.6002441444 |
| H | -5.1589496124 | -3.8347832281 | 0.4052600538 |
| N | 0.3305553103 | 4.1883465785 | 1.3666203054 |
| O | -0.0300608190 | 5.2296374586 | 1.9161209788 |
| O | 1.3102421480 | 3.5173829416 | 1.7139468978 |

09Zb

0 1

| | | | |
|---|---|---|---|
| C | -0.8935735415 | 0.4497986516 | 0.8484143109 |
| C | 1.3596563811 | -0.4466013085 | 0.7794845721 |
| N | 0.3940868150 | 0.1798705676 | 0.1426677530 |
| C | -1.2839154410 | 1.9554332164 | 0.7012026452 |
| C | 0.9349747980 | 3.1716094196 | 0.8042968865 |
| C | 2.6493184795 | -0.8324881276 | 0.2438492738 |
| O | 0.4340098276 | 0.5863146362 | -1.0745876325 |
| C | 3.0697072063 | -0.5969781921 | -1.0832347815 |
| C | 4.3334885777 | -1.0140590259 | -1.4960198449 |
| C | 5.1981798584 | -1.6634099346 | -0.6128283001 |
| C | 4.7924649875 | -1.9008863870 | 0.7039018239 |
| C | 3.5339689577 | -1.4908058157 | 1.1263360982 |
| H | -0.6900365515 | 0.3452920233 | 1.9181996744 |
| H | 1.1396844560 | -0.6999533464 | 1.8096450858 |
| H | 2.3970743190 | -0.0943485527 | -1.7641511277 |
| H | 4.6448117514 | -0.8280924069 | -2.5204404523 |
| H | 6.1817174523 | -1.9832606910 | -0.9461863119 |
| H | 5.4575165930 | -2.4062177344 | 1.3988408530 |
| H | 3.2216354738 | -1.6794725348 | 2.1510713524 |
| C | -0.2701523246 | 2.8942009860 | 1.3036280307 |
| H | -2.2337470102 | 2.0460100795 | 1.2325990987 |
| H | -0.5850226579 | 3.3557288548 | 2.2383269865 |
| H | 1.2801715025 | 2.7502760442 | -0.1336180479 |

| | | | |
|---|---|---|---|
| H | 1.6017868803 | 3.8556310684 | 1.3225552160 |
| C | -1.9547084655 | -0.5788774469 | 0.4832407462 |
| C | -2.0640515161 | -1.1371871635 | -0.7965183830 |
| C | -2.8372680577 | -0.9950613334 | 1.4876135705 |
| C | -3.0427618085 | -2.0948695640 | -1.0577922030 |
| H | -1.3817729839 | -0.8190424441 | -1.5764725035 |
| C | -3.8257561997 | -1.9422443214 | 1.2195392243 |
| H | -2.7528870477 | -0.5781412742 | 2.4898567047 |
| C | -3.9287527956 | -2.4968389334 | -0.0562473407 |
| H | -3.1168332085 | -2.5231877937 | -2.0535878539 |
| H | -4.5043674974 | -2.2526661524 | 2.0094203129 |
| H | -4.6926634839 | -3.2400444152 | -0.2681227578 |
| N | -1.7039323258 | 2.3287280301 | -0.7388991431 |
| O | -2.7374155914 | 1.7881909818 | -1.1238829272 |
| O | -1.0590788088 | 3.1509713403 | -1.3658326087 |

10a

0 1

| | | | |
|---|---|---|---|
| C | -1.0676455131 | 0.7011509132 | -0.0143115455 |
| C | 1.0527255297 | -0.2890593616 | 0.6534387398 |
| N | 0.1565432754 | -0.1350715452 | -0.2982611052 |
| C | -1.2545520299 | 1.7034358374 | -1.1714392948 |
| C | 0.7399694193 | 2.8056604648 | -2.2378325508 |
| C | 2.2765851568 | -1.0633188048 | 0.5860549389 |
| O | 0.2183601274 | -0.6472278721 | -1.4697428667 |
| C | 2.7092071287 | -1.7835068838 | -0.5481832135 |
| C | 3.9063288830 | -2.4954994108 | -0.5068128540 |
| C | 4.6930924716 | -2.5103068150 | 0.6466185738 |
| C | 4.2746523469 | -1.8005395141 | 1.7759715259 |
| C | 3.0826987137 | -1.0865780152 | 1.7453936213 |
| H | -0.8558436823 | 1.2628521532 | 0.8977812763 |
| H | 0.8249990831 | 0.2215439895 | 1.5812380424 |
| H | 2.0963889940 | -1.7687481770 | -1.4386482389 |
| H | 4.2269169564 | -3.0455537462 | -1.3878020266 |
| H | 5.6246354054 | -3.0696003183 | 0.6676317422 |
| H | 4.8783262308 | -1.8049603422 | 2.6796740944 |
| H | 2.7615909812 | -0.5364523850 | 2.6273271165 |
| C | -0.1543679936 | 2.7296460490 | -1.2448194841 |
| H | 0.7018238122 | 2.0790320953 | -3.0398046962 |
| H | 1.5265476484 | 3.5495300791 | -2.2787766676 |
| C | -2.2853247837 | -0.1763574375 | 0.2190596088 |
| C | -2.6660933208 | -1.1783550764 | -0.6851898173 |
| C | -3.0755259127 | 0.0494984121 | 1.3531899170 |
| C | -3.8128519865 | -1.9367132114 | -0.4498821778 |
| H | -2.0438346630 | -1.3720901959 | -1.5518700624 |
| C | -4.2281854609 | -0.7034326308 | 1.5819121872 |
| H | -2.7899043694 | 0.8239905385 | 2.0620444993 |
| C | -4.5995263597 | -1.7005587552 | 0.6796925420 |
| H | -4.0929177388 | -2.7158312210 | -1.1540632564 |

| | | | |
|---|---|---|---|
| H | -4.8303103941 | -0.5133565840 | 2.4665192815 |
| H | -5.4943659367 | -2.2914781073 | 0.8563300251 |
| H | -1.3154246289 | 1.1490138345 | -2.1088505829 |
| H | -2.2115926757 | 2.2119197205 | -1.0061197658 |
| O | -0.2077367686 | 3.5502784859 | -0.1477244731 |
| C | 0.7816428722 | 4.5617122258 | -0.0405757348 |
| H | 0.5784560097 | 5.0912082207 | 0.8923738448 |
| H | 1.7900949716 | 4.1288272418 | -0.0086937506 |
| H | 0.7220762009 | 5.2645581497 | -0.8817804119 |

10b

0 1

| | | | |
|---|---|---|---|
| C | -1.1940532354 | -0.1191762627 | 0.6025490042 |
| C | 1.0240206118 | 0.6279695872 | -0.0700550657 |
| N | -0.2792670851 | -0.0860626459 | -0.3425215702 |
| C | -0.5753225366 | 2.9258526985 | -2.2190562930 |
| C | 1.3130319632 | 1.5742271488 | -1.2550762750 |
| C | 2.1410294776 | -0.3649147603 | 0.1974100011 |
| O | -0.3815012838 | -0.6275073149 | -1.4985743938 |
| C | 2.4505240555 | -1.3968928495 | -0.7005203664 |
| C | 3.5086780600 | -2.2652023831 | -0.4329034714 |
| C | 4.2776471902 | -2.1113422479 | 0.7229627773 |
| C | 3.9768624681 | -1.0860471505 | 1.6196317968 |
| C | 2.9118699968 | -0.2225386210 | 1.3582500286 |
| H | -0.9284916473 | 0.4022967957 | 1.5140136714 |
| H | 0.8629783203 | 1.2306688216 | 0.8268622122 |
| H | 1.8407762052 | -1.5274256251 | -1.5875475958 |
| H | 3.7337618766 | -3.0660293854 | -1.1323911349 |
| H | 5.1036550229 | -2.7882592270 | 0.9243701887 |
| H | 4.5646121087 | -0.9600412250 | 2.5250969945 |
| H | 2.6776514698 | 0.5710360126 | 2.0652030865 |
| C | 0.3685182172 | 2.7506760823 | -1.2924786381 |
| H | 1.2275851895 | 1.0011338413 | -2.1798678375 |
| H | 2.3544102910 | 1.9040683135 | -1.1669359952 |
| C | -2.4873163258 | -0.7718965820 | 0.5446972996 |
| C | -2.9731135480 | -1.4934865148 | -0.5668291139 |
| C | -3.3067326754 | -0.6680434847 | 1.6900657586 |
| C | -4.2344579271 | -2.0836229898 | -0.5171909550 |
| H | -2.3505086875 | -1.5756920430 | -1.4466976583 |
| C | -4.5632361934 | -1.2606203859 | 1.7288758262 |
| H | -2.9455321325 | -0.1138396706 | 2.5536415736 |
| C | -5.0339903119 | -1.9731456886 | 0.6221179403 |
| H | -4.5957585093 | -2.6363614248 | -1.3805663117 |
| H | -5.1766989928 | -1.1675212033 | 2.6210678527 |
| H | -6.0161420112 | -2.4375336183 | 0.6494173234 |
| H | -0.7203543084 | 2.1926591250 | -3.0040590725 |
| H | -1.2414233234 | 3.7813282788 | -2.1786705731 |
| O | 0.4228080796 | 3.6290747232 | -0.2220981774 |
| C | 1.6843120781 | 4.2298985044 | 0.0510966157 |

| | | | |
|---|---|---|---|
| H | 1.5025507161 | 4.9765353092 | 0.8276231526 |
| H | 2.4240904707 | 3.5083267504 | 0.4241723264 |
| H | 2.0900848658 | 4.7280783113 | -0.8395909319 |

11Ea

0 1

| | | | |
|---|---|---|---|
| C | -0.4384398760 | -1.5768435075 | -0.7679471622 |
| C | -2.0907011137 | 0.1637144973 | -0.4383055102 |
| N | -1.0992286892 | -0.8469017748 | 0.1115501799 |
| C | 0.8144820760 | 2.0012184615 | 0.2365522304 |
| C | -1.6281711153 | 1.5654391083 | 0.0113981188 |
| O | -0.9922153691 | -0.8786020719 | 1.3571137781 |
| H | -0.6180784020 | -1.4577611286 | -1.8221594858 |
| H | -2.0438799411 | 0.0843709757 | -1.5282785432 |
| C | -0.2842847562 | 1.9683635121 | -0.5261783339 |
| H | -0.2000799022 | 2.2294329298 | -1.5796043923 |
| H | -1.6290234100 | 1.5763119968 | 1.1062900966 |
| H | -2.4048079443 | 2.2691828633 | -0.3173574586 |
| H | 0.7870563862 | 1.7361051482 | 1.2948956051 |
| O | 2.0233127336 | 2.3812298008 | -0.2494647545 |
| C | 3.1266621425 | 2.1729559424 | 0.6267581889 |
| H | 4.0023592935 | 2.5865253127 | 0.1234947448 |
| H | 2.9798555313 | 2.6936049097 | 1.5825646523 |
| H | 3.2883625053 | 1.1044457359 | 0.8175350854 |
| C | -3.4827044425 | -0.2055484518 | 0.0698971048 |
| H | -3.4988705183 | -0.2024581319 | 1.1626611018 |
| H | -4.2085620635 | 0.5269268278 | -0.2968221483 |
| H | -3.7861535337 | -1.1964898806 | -0.2830893698 |
| N | 0.5493685003 | -2.5541851521 | -0.4319248558 |
| O | 1.0173621418 | -3.1521642195 | -1.4135412476 |
| O | 0.8734217665 | -2.7407467036 | 0.7388403756 |

11Eb

0 1

| | | | |
|---|---|---|---|
| C | 0.0635893102 | -0.2709496097 | -0.5007277157 |
| C | -2.2663822087 | -0.7864179687 | -0.8615249813 |
| N | -1.2990946374 | -0.5619150484 | -0.0078380098 |
| C | 0.6465792896 | 0.9889780521 | 0.1403310785 |
| C | -1.0105776574 | 2.7621250372 | 0.7942968474 |
| O | -1.4346784426 | -0.5703538140 | 1.2634830532 |
| H | 0.0709979430 | -0.2180478210 | -1.5877374212 |
| H | -2.0143145886 | -0.7746836882 | -1.9137116170 |
| C | -0.2157299008 | 2.2021414222 | -0.1170664644 |
| H | -0.1404503253 | 2.6122273753 | -1.1241885486 |
| H | -1.1004890095 | 2.3533877941 | 1.7973617585 |
| H | -1.6126939195 | 3.6368573917 | 0.5632485661 |
| H | 0.7352959499 | 0.7945243775 | 1.2139599409 |

| | | | |
|---|---|---|---|
| O | 1.9212585408 | 1.1267035860 | -0.4739204627 |
| C | 2.8620417013 | 1.8289140627 | 0.3257033235 |
| H | 3.7816374015 | 1.8927255209 | -0.2605462942 |
| H | 2.5146434495 | 2.8437987941 | 0.5640717019 |
| H | 3.0648368348 | 1.2883193591 | 1.2605488170 |
| C | -3.6307747465 | -1.0652789764 | -0.3437576216 |
| H | -3.9677675423 | -0.2462575549 | 0.3046222598 |
| H | -4.3399299524 | -1.1979424695 | -1.1636375381 |
| H | -3.6270809352 | -1.9673012032 | 0.2823724757 |
| N | 0.9110431375 | -1.5053159648 | -0.1636416121 |
| O | 0.8067391139 | -2.4352083081 | -0.9570969065 |
| O | 1.5880231942 | -1.4907503461 | 0.8513443706 |

11Za

0 1

| | | | |
|---|---|---|---|
| C | 0.2798095237 | -1.2130870612 | -0.8511528111 |
| C | -1.9148802800 | -0.2247882395 | -0.5736301023 |
| N | -0.6081528073 | -0.7398499027 | 0.0061281657 |
| C | 0.2324648681 | 2.5319663482 | -0.4311145259 |
| C | -1.9538953489 | 1.3037713369 | -0.3542968759 |
| O | -0.4827936659 | -0.6422133442 | 1.2446873509 |
| H | 0.0481530582 | -1.2762368878 | -1.8998251883 |
| H | -1.8867745833 | -0.4499915514 | -1.6438328785 |
| C | -0.8414837954 | 2.0343493320 | -1.0559621403 |
| H | -0.8925433151 | 2.1466415218 | -2.1365346596 |
| H | -1.9110412906 | 1.4914193202 | 0.7216090458 |
| H | -2.9335769089 | 1.6454328003 | -0.7130978642 |
| H | 1.0505589781 | 3.0083882828 | -0.9774545643 |
| O | 0.3558272361 | 2.5331914066 | 0.9209550841 |
| C | 1.6671273349 | 2.2570073330 | 1.4151270325 |
| H | 2.4190670271 | 2.8745460161 | 0.9033810063 |
| H | 1.6566614254 | 2.5057371886 | 2.4777619245 |
| H | 1.9090119222 | 1.1955156299 | 1.2897402831 |
| N | 1.5891436385 | -1.6556478542 | -0.4961577276 |
| O | 2.0184774144 | -1.4958872468 | 0.6471692453 |
| O | 2.2184819945 | -2.1740544803 | -1.4311356815 |
| C | -3.0678102378 | -0.9631609115 | 0.1006352424 |
| H | -3.0460932287 | -0.7935874436 | 1.1796913140 |
| H | -4.0172104203 | -0.5916945813 | -0.2974100544 |
| H | -3.0145765391 | -2.0405730118 | -0.0869346206 |

11Zb

0 1

| | | | |
|---|---|---|---|
| C | 0.1160498983 | -0.2992642350 | -0.6677534521 |
| C | -2.0123653715 | -1.4062937785 | -0.6058831749 |
| N | -1.1097647669 | -0.7048612735 | 0.0407273601 |
| C | 0.4739471356 | 1.1970601475 | -0.5305510416 |

| | | | |
|---|---|---|---|
| C | -1.4957952799 | 2.6859225851 | -0.0816492352 |
| O | -1.2245592587 | -0.3392907951 | 1.2542008833 |
| H | 0.0156932772 | -0.5506830339 | -1.7253192897 |
| H | -1.8024752527 | -1.6744852515 | -1.6333884244 |
| C | -0.6971018725 | 2.0660867353 | -0.9483624093 |
| H | -0.8562473164 | 2.1625578034 | -2.0226285351 |
| H | -1.3459428577 | 2.5770083318 | 0.9876878439 |
| H | -2.3194944580 | 3.3096021187 | -0.4174476565 |
| H | 1.2984421519 | 1.3449019452 | -1.2449981828 |
| O | 0.9334541111 | 1.4336591023 | 0.7777233724 |
| C | 1.8689993125 | 2.4987920605 | 0.8606788605 |
| H | 1.4318789981 | 3.4512928685 | 0.5313528427 |
| H | 2.1511555047 | 2.5783993329 | 1.9126671775 |
| H | 2.7662020222 | 2.2851244556 | 0.2612737026 |
| N | 1.2955688479 | -1.1582871128 | -0.1884306225 |
| O | 1.0673233410 | -2.1144208685 | 0.5296976750 |
| O | 2.3877195756 | -0.8264007486 | -0.6380477107 |
| C | -3.2474762668 | -1.8117226573 | 0.1115786270 |
| H | -3.7599066886 | -0.9298695009 | 0.5177776331 |
| H | -3.9249868682 | -2.3559863820 | -0.5502689137 |
| H | -2.9982729181 | -2.4419928493 | 0.9758096706 |

12a

0 1

| | | | |
|---|---|---|---|
| C | -1.8934385019 | 0.0018789734 | -0.7336289305 |
| C | 0.2991917465 | 0.6604612000 | -0.1907233033 |
| N | -0.4106225871 | -0.3000281234 | -0.7658590602 |
| C | -2.5936507676 | -1.0528728367 | 0.1465788597 |
| C | -1.4454412551 | -1.8714064539 | 2.2241140926 |
| C | -2.2368830816 | -0.9914163567 | 1.6089150673 |
| O | -0.0448524631 | -1.3871945533 | -1.2737224034 |
| H | -3.6752783362 | -0.9142081088 | 0.0129308678 |
| H | -2.3350345913 | -2.0340767922 | -0.2646342792 |
| H | -1.0046384659 | -2.7101458246 | 1.6899246283 |
| H | -1.2082313091 | -1.7857821295 | 3.2812023927 |
| H | -2.6558252471 | -0.1634584424 | 2.1821727967 |
| C | -1.9579392305 | 1.4683190506 | -0.2779975418 |
| C | -0.5670042714 | 1.7676513663 | 0.3348737744 |
| H | -2.1365549562 | 2.1168916524 | -1.1408943751 |
| H | -0.5866942870 | 1.7499212641 | 1.4321899760 |
| H | -0.1797395355 | 2.7503037972 | 0.0484167067 |
| H | -2.7702789756 | 1.6425369934 | 0.4328662425 |
| C | 1.7541153746 | 0.6782160942 | -0.0028428763 |
| O | 2.3035497772 | 1.5943659304 | 0.5891703153 |
| O | 2.4039800282 | -0.3736240764 | -0.5319933478 |
| C | 3.8278420157 | -0.3581214735 | -0.3495596988 |
| H | 4.0806171890 | -0.3524029775 | 0.7143121936 |
| H | 4.1884100884 | -1.2698924111 | -0.8261429779 |
| H | 4.2684600139 | 0.5244249254 | -0.8217475183 |

| | | | |
|---|---|---|---|
| H | -2.2275353713 | -0.1386566873 | -1.7652806010 |

12b

0 1

| | | | |
|---|---|---|---|
| C | -1.5957451755 | 0.8310216008 | -1.4045616403 |
| C | -0.0601179298 | 0.2675664644 | 0.2802352314 |
| N | -0.6390146461 | -0.0043586207 | -1.1028154526 |
| C | -2.6686568312 | -1.9851948440 | 0.8384201261 |
| C | -0.4118005025 | -0.9419939849 | 1.1768592080 |
| C | -1.8855550211 | -1.0963630550 | 1.4520051598 |
| O | -0.1876829499 | -0.9851416037 | -1.7652601190 |
| H | -3.7285998717 | -2.0666874673 | 1.0649858932 |
| H | -2.2740446393 | -2.6617961468 | 0.0837202983 |
| H | -0.0261119956 | -1.8330502898 | 0.6761074968 |
| H | 0.1400767713 | -0.8306899009 | 2.1181825531 |
| H | -2.3205785013 | -0.4328044233 | 2.2004617324 |
| C | -1.8838886046 | 1.8192705172 | -0.3261722599 |
| C | -0.7052575257 | 1.6160467350 | 0.6645291252 |
| H | -1.9261948443 | 2.8484281069 | -0.7033970834 |
| H | -1.0332409809 | 1.6228569930 | 1.7070879615 |
| H | 0.0306615085 | 2.4104507732 | 0.5300474138 |
| H | -2.8552669031 | 1.6149365330 | 0.1458880882 |
| C | 1.4579903469 | 0.4234074403 | 0.0445240269 |
| O | 1.9515887841 | 1.4143710019 | -0.4477205667 |
| O | 2.1596146607 | -0.6490047966 | 0.4344853899 |
| C | 3.5719048316 | -0.5931977300 | 0.1631341881 |
| H | 4.0242753232 | 0.2644307704 | 0.6674562540 |
| H | 3.9781524482 | -1.5284550734 | 0.5480987802 |
| H | 3.7432608346 | -0.5106229255 | -0.9127055143 |
| H | -2.1063605867 | 0.7123259259 | -2.3514222909 |

13a

0 1

| | | | |
|---|---|---|---|
| C | 1.3200990867 | -0.2186086454 | 0.4309943325 |
| C | -0.8753630130 | 0.5536850071 | 0.0935108456 |
| N | 0.2221829112 | -0.4320047504 | -0.2400578804 |
| C | 0.8440101266 | 2.0009812533 | -2.5874946967 |
| C | -1.1384716867 | 1.4076447768 | -1.1680041837 |
| C | 0.0245853199 | 2.2767337100 | -1.5713928795 |
| O | 0.0237063339 | -1.3169090537 | -1.1389287342 |
| H | 1.6718978997 | 2.6551906330 | -2.8484272414 |
| H | 0.7144679670 | 1.1062809969 | -3.1923126813 |
| H | -1.3887876616 | 0.7167958289 | -1.9765671642 |
| H | -2.0255025041 | 2.0241646327 | -0.9774600629 |
| H | 0.1909139816 | 3.1822153177 | -0.9866188433 |
| C | 2.5029797061 | -1.0843494619 | 0.1530444019 |
| C | 1.2195496693 | 0.9533629979 | 1.3499981228 |

| | | | |
|---|---|---|---|
| C | -0.2941053153 | 1.2993402537 | 1.3172302314 |
| C | 4.7087180377 | -1.5023699485 | 0.8357812564 |
| O | 3.5448001594 | -0.7283714722 | 1.0373181617 |
| H | 2.2152893393 | -2.1452490561 | 0.2625733785 |
| H | 2.8091716264 | -0.9633690937 | -0.9030700751 |
| H | 1.5718288627 | 0.7135632095 | 2.3588080205 |
| H | -0.4722629415 | 2.3758758636 | 1.2532371980 |
| H | 4.5174903787 | -2.5743524754 | 1.0021846633 |
| H | 5.4529767823 | -1.1562988374 | 1.5573622885 |
| H | 5.1091191794 | -1.3760145721 | -0.1828514408 |
| H | -0.7775167813 | 0.9257444257 | 2.2214995502 |
| H | 1.8433017861 | 1.7823884771 | 0.9894914304 |
| C | -2.0841937434 | -0.3187285111 | 0.4968372068 |
| O | -2.1389512645 | -0.9269200889 | 1.5430639793 |
| O | -3.0532062655 | -0.3324372339 | -0.4292343725 |
| C | -4.1720818637 | -1.1886391910 | -0.1357199431 |
| H | -4.6514143398 | -0.8839819037 | 0.7981061835 |
| H | -4.8545436452 | -1.0731183260 | -0.9776909833 |
| H | -3.8379601286 | -2.2250567624 | -0.0473580690 |

13b

0 1

| | | | |
|---|---|---|---|
| C | 1.2314398679 | 0.2403429041 | -0.0538934485 |
| C | -1.0126057138 | 0.0098818771 | 0.6464520958 |
| N | -0.1318728193 | -0.4067211156 | -0.2507143541 |
| C | 1.4744946082 | 1.1973594901 | -1.2468848488 |
| C | -0.4200176014 | 2.5680533191 | -2.1637088465 |
| C | 0.5798585762 | 2.4069354489 | -1.2952567344 |
| O | -0.2759644932 | -1.1989046195 | -1.2166971941 |
| H | 2.5265161368 | 1.5006479517 | -1.1851581155 |
| H | 1.3511618079 | 0.6063676690 | -2.1609887381 |
| H | -0.6538348204 | 1.8111348770 | -2.9093352455 |
| H | -1.0395892644 | 3.4608963727 | -2.1634140830 |
| H | 0.7795743150 | 3.1938193961 | -0.5670127929 |
| C | 2.2666298892 | -0.8930303853 | -0.0706067943 |
| C | 1.0984072247 | 0.9139229241 | 1.3253983533 |
| C | -0.4207505421 | 1.0063706458 | 1.5984112168 |
| C | 4.5710491612 | -1.2691532722 | 0.2273454839 |
| O | 3.5222467270 | -0.3207510121 | 0.2361251130 |
| H | 1.9910528772 | -1.6544092070 | 0.6788393317 |
| H | 2.2761834624 | -1.3796264248 | -1.0555952224 |
| H | 1.5847242499 | 0.2922642195 | 2.0830270690 |
| H | -0.8188757399 | 2.0085411025 | 1.3954024941 |
| H | 4.4109782889 | -2.0570063419 | 0.9801182728 |
| H | 5.4919059990 | -0.7307106325 | 0.4648174905 |
| H | 4.6766897654 | -1.7471190495 | -0.7588864619 |
| H | -0.6811096778 | 0.7729010810 | 2.6354063267 |
| H | 1.5910613419 | 1.8884293483 | 1.3500142943 |
| C | -2.4294276123 | -0.3682458116 | 0.7203974251 |

| | | | |
|---|---|---|---|
| O | -3.1692631300 | 0.1181594003 | 1.5612304117 |
| O | -2.8202789828 | -1.2759284941 | -0.1917662337 |
| C | -4.2056725226 | -1.6457267135 | -0.1204417177 |
| H | -4.8465134214 | -0.7744176284 | -0.2817178994 |
| H | -4.3481327850 | -2.3799835644 | -0.9135737067 |
| H | -4.4398961723 | -2.0804887550 | 0.8552650588 |

TS01

0 1

| | | | |
|---|---|---|---|
| C | -1.1852290000 | 0.6631950000 | 0.33108800 |
| C | 1.1851910000 | 0.6629340000 | 0.33116100 |
| N | -0.0000320000 | -0.0156360000 | -0.02631300 |
| C | -1.2136280000 | 1.9940930000 | -0.85311500 |
| C | 1.2136990000 | 1.9940060000 | -0.85330500 |
| C | 2.5003800000 | -0.0557050000 | 0.27231500 |
| O | -0.0000980000 | -0.7012110000 | -1.12943500 |
| C | 2.8604440000 | -0.9494880000 | -0.74930300 |
| C | 4.1202570000 | -1.5495560000 | -0.74023200 |
| C | 5.0416460000 | -1.2614460000 | 0.26764100 |
| C | 4.6944980000 | -0.3672770000 | 1.28176600 |
| C | 3.4341600000 | 0.2273770000 | 1.28221200 |
| H | -1.0568220000 | 1.1389340000 | 1.30206500 |
| H | 1.0568450000 | 1.1388240000 | 1.30206300 |
| H | 2.1399080000 | -1.1823940000 | -1.52159600 |
| H | 4.3820210000 | -2.2475180000 | -1.53126000 |
| H | 6.0221110000 | -1.7303320000 | 0.26430500 |
| H | 5.4004580000 | -0.1365620000 | 2.07515100 |
| H | 3.1683970000 | 0.9185620000 | 2.07948400 |

| | | | |
|---|---|---|---|
| C | 0.0001250000 | 2.7238300000 | -0.70235900 |
| H | -2.1429890000 | 2.5391270000 | -0.68572000 |
| H | -1.2602660000 | 1.3443780000 | -1.73711000 |
| H | 0.0002100000 | 3.6707860000 | -0.16600400 |
| H | 1.2600750000 | 1.3440210000 | -1.73711500 |
| H | 2.1432000000 | 2.5388290000 | -0.68604000 |
| C | -2.5004130000 | -0.0555090000 | 0.27232800 |
| C | -2.8608940000 | -0.9484010000 | -0.74992200 |
| C | -3.4337520000 | 0.2266340000 | 1.28288400 |
| C | -4.1206760000 | -1.5485280000 | -0.74083600 |
| H | -2.1406960000 | -1.1806320000 | -1.52273200 |
| C | -4.6940610000 | -0.3680930000 | 1.28248000 |
| H | -3.1676710000 | 0.9171250000 | 2.08065100 |
| C | -5.0416240000 | -1.2613690000 | 0.26771400 |
| H | -4.3827650000 | -2.2457910000 | -1.53237500 |
| H | -5.3996750000 | -0.1381170000 | 2.07638800 |
| H | -6.0220660000 | -1.7303010000 | 0.26440000 |

TS02

0 1

| | | | |
|---|---|---|---|
| C | -0.1116700000 | 0.9855540000 | 0.27136100 |
| C | 2.2357910000 | 0.6702630000 | 0.32093000 |
| N | 0.9761980000 | 0.1445570000 | -0.04150200 |
| C | 0.0511790000 | 2.2773580000 | -0.95718800 |
| C | 2.4561820000 | 1.9537410000 | -0.89930700 |
| C | 3.4446830000 | -0.2163160000 | 0.30235700 |

| | | | |
|---|---|---|---|
| O | 0.9011490000 | -0.5700500000 | -1.12299500 |
| C | 3.7111480000 | -1.1570830000 | -0.70569500 |
| C | 4.8788940000 | -1.9191810000 | -0.65798400 |
| C | 5.8008800000 | -1.7491200000 | 0.37591400 |
| C | 5.5475660000 | -0.8094490000 | 1.37648700 |
| C | 4.3784800000 | -0.0520530000 | 1.33822000 |
| H | 0.0596340000 | 1.4730750000 | 1.22926300 |
| H | 2.1569590000 | 1.1860240000 | 1.27662300 |
| H | 2.9908290000 | -1.2994130000 | -1.49987900 |
| H | 5.0679540000 | -2.6510560000 | -1.43883200 |
| H | 6.7096020000 | -2.3444460000 | 0.40287100 |
| H | 6.2549640000 | -0.6691090000 | 2.18930800 |
| H | 4.1848940000 | 0.6738310000 | 2.12534500 |
| C | 1.3474110000 | 2.8404770000 | -0.79172400 |
| H | -0.8004050000 | 2.9452010000 | -0.82501400 |
| H | -0.0634600000 | 1.6114360000 | -1.82235400 |
| H | 1.4621010000 | 3.7930850000 | -0.27859900 |
| H | 2.4317910000 | 1.2789560000 | -1.76476000 |
| H | 3.4459950000 | 2.3757300000 | -0.72502400 |
| C | -1.5069910000 | 0.4445860000 | 0.20156800 |
| C | -1.9461920000 | -0.4684290000 | -0.77319100 |
| C | -2.4297800000 | 0.9234360000 | 1.14894100 |
| C | -3.2700800000 | -0.8995150000 | -0.78620300 |
| H | -1.2356430000 | -0.8482580000 | -1.49410000 |
| C | -3.7555910000 | 0.5077980000 | 1.13923800 |

| | | | |
|---|---|---|---|
| H | -2.1037750000 | 1.6291560000 | 1.90885600 |
| C | -4.1580930000 | -0.4043710000 | 0.16565800 |
| H | -3.6219490000 | -1.6093470000 | -1.52476400 |
| H | -4.4721750000 | 0.8682080000 | 1.86663800 |
| N | -5.5578500000 | -0.8540430000 | 0.14571900 |
| O | -5.8889970000 | -1.6580840000 | -0.72526000 |
| O | -6.3169820000 | -0.3984730000 | 1.00183200 |

TS03

0 1

| | | | |
|---|---|---|---|
| C | -0.3785380000 | 0.8752140000 | 0.31911300 |
| C | 1.9790940000 | 0.6255090000 | 0.39452800 |
| N | 0.7481580000 | 0.1118060000 | -0.06176800 |
| C | -0.2192490000 | 2.3037810000 | -0.73061700 |
| C | 2.1936700000 | 2.0522470000 | -0.66122900 |
| C | 3.2168380000 | -0.2164530000 | 0.30848400 |
| O | 0.7216400000 | -0.4705140000 | -1.22272400 |
| C | 3.5216610000 | -1.0557770000 | -0.77537800 |
| C | 4.7144770000 | -1.7799690000 | -0.78423200 |
| C | 5.6250540000 | -1.6703490000 | 0.26775400 |
| C | 5.3336590000 | -0.8309870000 | 1.34434800 |
| C | 4.1392460000 | -0.1134530000 | 1.36252800 |
| H | -0.2458870000 | 1.2430270000 | 1.33557500 |
| H | 1.8604800000 | 1.0254780000 | 1.40020600 |
| H | 2.8088310000 | -1.1495670000 | -1.58348000 |
| H | 4.9324780000 | -2.4339850000 | -1.62459300 |

| | | | |
|---|---|---|---|
| H | 6.5537250000 | -2.2346800000 | 0.25014900 |
| H | 6.0314110000 | -0.7385420000 | 2.17245500 |
| H | 3.9162880000 | 0.5336640000 | 2.20847400 |
| C | 1.0559250000 | 2.8873550000 | -0.47703200 |
| H | -1.0942060000 | 2.9255540000 | -0.53911200 |
| H | -0.2972920000 | 1.7496970000 | -1.67534800 |
| H | 1.1310680000 | 3.7740010000 | 0.14968200 |
| H | 2.2081480000 | 1.4810670000 | -1.59880600 |
| H | 3.1669700000 | 2.4769510000 | -0.41432500 |
| C | -1.7511240000 | 0.3017480000 | 0.14650000 |
| C | -2.1679060000 | -0.4240890000 | -0.98598300 |
| C | -2.6919660000 | 0.5454270000 | 1.15390400 |
| C | -3.4705320000 | -0.8930310000 | -1.08362300 |
| H | -1.4512180000 | -0.6362490000 | -1.76788600 |
| C | -4.0069520000 | 0.0861950000 | 1.06272600 |
| H | -2.3975040000 | 1.1053070000 | 2.03927800 |
| C | -4.4019270000 | -0.6414680000 | -0.06545700 |
| H | -3.7940300000 | -1.4624810000 | -1.94952300 |
| H | -4.7004630000 | 0.2940390000 | 1.86953500 |
| O | -5.6545310000 | -1.1436350000 | -0.26793900 |
| C | -6.6376640000 | -0.9195780000 | 0.72932900 |
| H | -7.5478830000 | -1.4002840000 | 0.36615100 |
| H | -6.3495440000 | -1.3684440000 | 1.68924900 |
| H | -6.8279270000 | 0.1520610000 | 0.87602400 |

TS04

0 1

| | | | |
|---|---|---|---|
| C | -2.4133940000 | -0.3337860000 | 0.77296300 |
| C | -0.2061620000 | 0.4523840000 | 0.35897900 |
| N | -1.1388650000 | -0.6278130000 | 0.29241800 |
| C | -3.0181520000 | 0.8310310000 | -0.51005300 |
| C | -0.7178310000 | 1.5014330000 | -0.88449800 |
| C | 1.2559980000 | 0.1323230000 | 0.19730100 |
| O | -1.0484760000 | -1.4581940000 | -0.69925000 |
| C | -3.3876040000 | -1.4906970000 | 0.80391100 |
| C | 1.7563890000 | -0.6989590000 | -0.81710600 |
| C | 3.1300500000 | -0.9189760000 | -0.92641700 |
| C | 4.0227810000 | -0.3090280000 | -0.04354800 |
| C | 3.5333430000 | 0.5251430000 | 0.96253800 |
| C | 2.1609020000 | 0.7409610000 | 1.07990100 |
| H | -2.3731700000 | 0.2413880000 | 1.69697100 |
| H | -0.3632660000 | 0.9873960000 | 1.29613900 |
| H | -3.0610140000 | -2.2392860000 | 1.53471800 |
| H | -3.4387300000 | -1.9731350000 | -0.17334900 |
| H | -4.3819010000 | -1.1376750000 | 1.09355100 |
| H | 1.0621470000 | -1.1850560000 | -1.48974300 |
| H | 3.5035010000 | -1.5725390000 | -1.71055000 |
| H | 5.0915010000 | -0.4827520000 | -0.13798800 |
| H | 4.2166220000 | 1.0040560000 | 1.65893100 |
| H | 1.7863570000 | 1.3886200000 | 1.87008600 |
| C | -2.0811020000 | 1.8811650000 | -0.61367300 |
| H | -4.0416430000 | 1.0913560000 | -0.23664600 |

| | | | |
|---|---|---|---|
| H | -2.9614150000 | 0.0694700000 | -1.29999400 |
| H | -2.2928330000 | 2.8511940000 | -0.16868100 |
| H | -0.6005760000 | 0.8195160000 | -1.73806200 |
| H | 0.0029800000 | 2.3197340000 | -0.93009700 |

TS05

0 1

| | | | |
|---|---|---|---|
| C | -1.6954810000 | 0.1562530000 | 0.50713000 |
| C | 0.6184530000 | 0.6221230000 | 0.41150200 |
| N | -0.4689730000 | -0.2249990000 | 0.00572400 |
| C | -2.0287880000 | 1.7378240000 | -0.45991100 |
| C | 0.3843240000 | 2.0245310000 | -0.50656100 |
| C | 2.0163230000 | 0.0958690000 | 0.22143100 |
| O | -0.4255550000 | -0.7692960000 | -1.16351100 |
| C | -2.8772600000 | -0.7615860000 | 0.24739900 |
| C | 2.4685320000 | -0.4728320000 | -0.97945500 |
| C | 3.7907530000 | -0.9034770000 | -1.09307500 |
| C | 4.6799320000 | -0.7637410000 | -0.02618000 |
| C | 4.2390310000 | -0.1923770000 | 1.16818300 |
| C | 2.9165750000 | 0.2323850000 | 1.28837700 |
| H | -1.6698020000 | 0.4413650000 | 1.55481100 |
| H | 0.4693720000 | 0.8996920000 | 1.45562300 |
| H | 1.7762390000 | -0.5983940000 | -1.80130100 |
| H | 4.1264270000 | -1.3510040000 | -2.02478200 |
| H | 5.7091020000 | -1.0986840000 | -0.12426400 |
| H | 4.9201620000 | -0.0810110000 | 2.00750100 |

| | | | |
|---|---|---|---|
| H | 2.5782390000 | 0.6726440000 | 2.22410300 |
| C | -0.9228260000 | 2.5654560000 | -0.20560000 |
| H | -3.0177520000 | 2.0815530000 | -0.15926100 |
| H | -2.0192810000 | 1.1914110000 | -1.41003300 |
| H | -1.0130740000 | 3.4024660000 | 0.48334900 |
| H | 0.4677550000 | 1.5969740000 | -1.51485100 |
| H | 1.2254140000 | 2.6851710000 | -0.28939900 |
| F | -3.2361390000 | -0.8611450000 | -1.03816700 |
| F | -3.9431670000 | -0.2649750000 | 0.92697700 |
| F | -2.6432460000 | -2.0022560000 | 0.71420000 |

TS06

0 1

| | | | |
|---|---|---|---|
| C | -1.7543430000 | 0.0363620000 | 0.40928200 |
| C | 0.5689950000 | 0.5273300000 | 0.37648400 |
| N | -0.4955940000 | -0.3381270000 | -0.02684600 |
| C | -2.0526830000 | 1.5752270000 | -0.56387700 |
| C | 0.3489730000 | 1.9165610000 | -0.58114600 |
| C | 1.9786030000 | 0.0168710000 | 0.23825000 |
| O | -0.4180970000 | -0.9336040000 | -1.17916000 |
| C | -2.8708900000 | -0.9218470000 | 0.04570000 |
| C | 2.4616830000 | -0.6061370000 | -0.92315800 |
| C | 3.7931010000 | -1.0178930000 | -0.99302400 |
| C | 4.6622040000 | -0.8058970000 | 0.07853900 |
| C | 4.1909950000 | -0.1802590000 | 1.23359500 |
| C | 2.8593370000 | 0.2257380000 | 1.30996500 |

| | | | |
|---|---|---|---|
| H | -1.7801140000 | 0.3384890000 | 1.45430100 |
| H | 0.3893300000 | 0.8350760000 | 1.40717600 |
| H | -2.6253730000 | -1.9277840000 | 0.42650700 |
| H | -2.9675370000 | -1.0024670000 | -1.04512600 |
| H | 1.7821590000 | -0.7860420000 | -1.74560500 |
| H | 4.1523600000 | -1.5074370000 | -1.89453300 |
| H | 5.6987500000 | -1.1264710000 | 0.01467500 |
| H | 4.8559780000 | -0.0119750000 | 2.07657400 |
| H | 2.4974800000 | 0.7089260000 | 2.21528600 |
| C | -0.9693440000 | 2.4392830000 | -0.31285800 |
| H | -3.0555730000 | 1.9019930000 | -0.29308600 |
| H | -2.0101370000 | 1.0321480000 | -1.51749300 |
| H | -1.0854040000 | 3.2814720000 | 0.36641000 |
| H | 0.4606730000 | 1.4652100000 | -1.57619300 |
| H | 1.1796160000 | 2.5890280000 | -0.35921100 |
| C | -5.1786690000 | -1.2378330000 | 0.36168200 |
| H | -6.0376720000 | -0.7778830000 | 0.85677200 |
| H | -5.3769500000 | -1.2987700000 | -0.72002800 |
| H | -5.0476430000 | -2.2612210000 | 0.74779700 |
| O | -4.0554750000 | -0.4277000000 | 0.64129700 |

TS07E

0 1

| | | | |
|---|---|---|---|
| C | -0.6551880000 | -0.1503430000 | 0.47442300 |
| C | 1.6793520000 | 0.2791060000 | 0.50212600 |
| N | 0.6259240000 | -0.4492870000 | -0.03803400 |

| | | | |
|---|---|---|---|
| C | -1.0403500000 | 1.4662650000 | -0.39394600 |
| C | 1.3780220000 | 1.9155240000 | -0.37658800 |
| C | 3.1005980000 | -0.0976890000 | 0.25617600 |
| O | 0.7137580000 | -0.8679420000 | -1.26331300 |
| C | 3.5723860000 | -0.6855830000 | -0.92981900 |
| C | 4.9292140000 | -0.9787930000 | -1.07188700 |
| C | 5.8362370000 | -0.6812640000 | -0.05392300 |
| C | 5.3770560000 | -0.0891360000 | 1.12432000 |
| C | 4.0224860000 | 0.1970360000 | 1.27589500 |
| H | -0.5773290000 | 0.1276090000 | 1.52328000 |
| H | 1.4993560000 | 0.5071120000 | 1.54954500 |
| H | 2.8649630000 | -0.9252590000 | -1.71174700 |
| H | 5.2782510000 | -1.4430260000 | -1.99065000 |
| H | 6.8918210000 | -0.9093230000 | -0.17586600 |
| H | 6.0710120000 | 0.1442990000 | 1.92746000 |
| H | 3.6715260000 | 0.6522410000 | 2.19985300 |
| C | 0.0684140000 | 2.2998070000 | -0.04048500 |
| H | -0.0683080000 | 3.0664320000 | 0.72039800 |
| H | 1.5254250000 | 1.4665960000 | -1.36428200 |
| H | 2.1948000000 | 2.5637750000 | -0.06191700 |
| C | -1.7045770000 | -1.2033670000 | 0.28641100 |
| C | -1.9512500000 | -1.8518380000 | -0.93516200 |
| C | -2.4654640000 | -1.5722550000 | 1.40823000 |
| C | -2.9393190000 | -2.8328890000 | -1.02071000 |
| H | -1.3556860000 | -1.5934130000 | -1.79952200 |

| | | | |
|---|---|---|---|
| C | -3.4571460000 | -2.5476730000 | 1.31686400 |
| H | -2.2735480000 | -1.0945670000 | 2.36649500 |
| C | -3.6992780000 | -3.1816790000 | 0.09713800 |
| H | -3.1163310000 | -3.3272700000 | -1.97236900 |
| H | -4.0324530000 | -2.8163100000 | 2.19888800 |
| H | -4.4699910000 | -3.9440440000 | 0.02029200 |
| H | -0.9419500000 | 1.0265820000 | -1.39554800 |
| C | -3.5740440000 | 1.2040580000 | -0.79010800 |
| H | -4.5239210000 | 1.7510930000 | -0.74821100 |
| H | -3.3555840000 | 1.0015030000 | -1.84534600 |
| H | -3.7205170000 | 0.2474690000 | -0.28803200 |
| C | -2.4608010000 | 2.0563950000 | -0.14593400 |
| C | -2.5097240000 | 3.4451590000 | -0.83379800 |
| H | -1.7959970000 | 4.1481130000 | -0.39325100 |
| H | -2.2791140000 | 3.3624800000 | -1.90244300 |
| H | -3.5136780000 | 3.8776800000 | -0.73950200 |
| C | -2.7601220000 | 2.2338830000 | 1.35619000 |
| H | -3.7286190000 | 2.7294850000 | 1.49261800 |
| H | -2.8101010000 | 1.2700800000 | 1.87356200 |
| H | -2.0045620000 | 2.8508500000 | 1.85656100 |

TS07Z

0 1

| | | | |
|---|---|---|---|
| C | 0.7906810000 | -0.4822530000 | -0.78186800 |
| C | -1.5556040000 | -0.3035250000 | -0.79122100 |
| N | -0.4074940000 | -0.6028030000 | -0.09315800 |

| | | | |
|---|---|---|---|
| C | 1.1117700000 | 1.6586400000 | -0.86097600 |
| C | -1.3770930000 | 1.8111690000 | -0.91452500 |
| C | -2.9141200000 | -0.5284610000 | -0.26776300 |
| O | -0.4237210000 | -0.7201910000 | 1.19772400 |
| C | -3.2659260000 | -0.5278580000 | 1.09579200 |
| C | -4.5957490000 | -0.7109180000 | 1.47476200 |
| C | -5.5967780000 | -0.8894970000 | 0.51899000 |
| C | -5.2598980000 | -0.8860200000 | -0.83668300 |
| C | -3.9352120000 | -0.7065910000 | -1.22269400 |
| H | 0.6307090000 | -0.5295160000 | -1.85269200 |
| H | -1.4555270000 | -0.4837650000 | -1.85454900 |
| H | -2.4887480000 | -0.4038090000 | 1.83608000 |
| H | -4.8490860000 | -0.7143450000 | 2.53179600 |
| H | -6.6296100000 | -1.0315130000 | 0.82574900 |
| H | -6.0275810000 | -1.0275050000 | -1.59281600 |
| H | -3.6816040000 | -0.7075720000 | -2.28092300 |
| C | -0.1221170000 | 1.9379500000 | -1.49945100 |
| H | -0.1059000000 | 1.9025190000 | -2.59099100 |
| H | -1.5203970000 | 1.9355590000 | 0.15251600 |
| H | -2.2613270000 | 2.0070730000 | -1.51576700 |
| C | 2.0149310000 | -1.2053070000 | -0.37223000 |
| C | 2.2064820000 | -1.8261810000 | 0.87577500 |
| C | 3.0372060000 | -1.3106160000 | -1.33523100 |
| C | 3.3838760000 | -2.5275660000 | 1.13592000 |
| H | 1.4216240000 | -1.7571360000 | 1.61691600 |

| | | | |
|---|---|---|---|
| C | 4.2166060000 | -1.9974570000 | -1.06271600 |
| H | 2.9004140000 | -0.8505890000 | -2.31200400 |
| C | 4.3964250000 | -2.6112750000 | 0.17898900 |
| H | 3.5102220000 | -3.0092890000 | 2.10219100 |
| H | 4.9907600000 | -2.0614100000 | -1.82292000 |
| H | 5.3140760000 | -3.1517030000 | 0.39522900 |
| H | 1.9460850000 | 1.5903160000 | -1.56319200 |
| C | 1.6084570000 | 2.3194010000 | 0.44514300 |
| C | 0.6123410000 | 2.3439170000 | 1.62068500 |
| H | 1.1150630000 | 2.7519020000 | 2.50628500 |
| H | -0.2442930000 | 2.9943730000 | 1.41502600 |
| H | 0.2483030000 | 1.3411930000 | 1.85861700 |
| C | 2.9255600000 | 1.6780190000 | 0.92571300 |
| H | 3.6460220000 | 1.5656170000 | 0.10666400 |
| H | 3.3857180000 | 2.3162780000 | 1.68940900 |
| H | 2.7629240000 | 0.6934780000 | 1.36670400 |
| C | 1.9218370000 | 3.7876460000 | 0.04182800 |
| H | 2.3235140000 | 4.3311390000 | 0.90611200 |
| H | 2.6691000000 | 3.8367280000 | -0.75978400 |
| H | 1.0197230000 | 4.3038330000 | -0.30278800 |

TS08E

0 1

| | | | |
|---|---|---|---|
| C | -0.8692920000 | 0.1521500000 | 0.54239100 |
| C | 1.4826840000 | 0.3523220000 | 0.53488900 |
| N | 0.3600770000 | -0.2904400000 | 0.03526300 |

| | | | |
|---|---|---|---|
| C | -1.1265890000 | 1.7562200000 | -0.40335000 |
| C | 1.2957600000 | 2.0203440000 | -0.44566900 |
| C | 2.8607170000 | -0.1404670000 | 0.27824200 |
| O | 0.3919600000 | -0.7803100000 | -1.16787000 |
| C | 3.2594760000 | -0.8317480000 | -0.87928100 |
| C | 4.5868360000 | -1.2348220000 | -1.02964600 |
| C | 5.5382480000 | -0.9476420000 | -0.05007300 |
| C | 5.1531130000 | -0.2530900000 | 1.09862100 |
| C | 3.8281740000 | 0.1433950000 | 1.25941600 |
| H | -0.7617130000 | 0.5007200000 | 1.56673500 |
| H | 1.3375180000 | 0.6590520000 | 1.56655700 |
| H | 2.5187940000 | -1.0622410000 | -1.63254500 |
| H | 4.8776940000 | -1.7777380000 | -1.92528200 |
| H | 6.5704720000 | -1.2623810000 | -0.17864200 |
| H | 5.8817760000 | -0.0256850000 | 1.87221100 |
| H | 3.5348420000 | 0.6775960000 | 2.16086900 |
| C | 0.0327120000 | 2.5328570000 | -0.12026600 |
| H | -0.0733370000 | 3.3363780000 | 0.60580500 |
| H | 1.3955700000 | 1.4902030000 | -1.39679200 |
| H | 2.1718400000 | 2.6026100000 | -0.16714100 |
| C | -2.1039230000 | -0.6690100000 | 0.37664500 |
| C | -2.3456780000 | -1.5368290000 | -0.70260100 |
| C | -3.0983260000 | -0.5243550000 | 1.36004200 |
| C | -3.5446840000 | -2.2479030000 | -0.77343000 |
| H | -1.5813900000 | -1.6573140000 | -1.45845900 |

| | | | |
|---|---|---|---|
| C | -4.2981320000 | -1.2272980000 | 1.27686800 |
| H | -2.9283210000 | 0.1503290000 | 2.19501300 |
| C | -4.5258270000 | -2.0957860000 | 0.20765800 |
| H | -3.7115930000 | -2.9245630000 | -1.60765200 |
| H | -5.0514900000 | -1.1004330000 | 2.04992200 |
| H | -5.4587690000 | -2.6493790000 | 0.14113900 |
| H | -1.1677880000 | 1.2667310000 | -1.38468300 |
| O | -2.3267690000 | 2.2843490000 | 0.04362800 |
| C | -3.3874820000 | 2.2534690000 | -0.90493500 |
| H | -4.2724320000 | 2.6366770000 | -0.39187200 |
| H | -3.1635080000 | 2.8961920000 | -1.76786300 |
| H | -3.5869520000 | 1.2331710000 | -1.25335000 |

TS08Z

0 1

| | | | |
|---|---|---|---|
| C | 1.0007800000 | -0.0457650000 | -0.78479800 |
| C | -1.3693110000 | 0.1035360000 | -0.74009900 |
| N | -0.1976420000 | -0.3394040000 | -0.09734100 |
| C | 1.1558580000 | 1.7861840000 | -0.63283900 |
| C | -1.2647270000 | 1.9279560000 | -0.56354700 |
| C | -2.7042850000 | -0.3432450000 | -0.24319900 |
| O | -0.2031660000 | -0.5825880000 | 1.15884600 |
| C | -3.0581840000 | -0.4030130000 | 1.11553300 |
| C | -4.3483070000 | -0.7838340000 | 1.48599300 |
| C | -5.3048810000 | -1.1008640000 | 0.52044800 |
| C | -4.9648610000 | -1.0351680000 | -0.83192400 |

| | | | |
|---|---|---|---|
| C | -3.6771850000 | -0.6579010000 | -1.20685900 |
| H | 0.8497090000 | -0.1668280000 | -1.85498600 |
| H | -1.2818790000 | -0.0290120000 | -1.81566700 |
| H | -2.3150140000 | -0.1738530000 | 1.86642600 |
| H | -4.6051450000 | -0.8332250000 | 2.54094700 |
| H | -6.3071450000 | -1.3971970000 | 0.81845100 |
| H | -5.6988370000 | -1.2811010000 | -1.59473400 |
| H | -3.4191890000 | -0.6112260000 | -2.26290900 |
| C | -0.0495510000 | 2.3127010000 | -1.18730400 |
| H | -0.0524950000 | 2.5280280000 | -2.25500800 |
| H | -1.2841960000 | 1.9663880000 | 0.52807600 |
| H | -2.1925460000 | 2.2808140000 | -1.01292900 |
| C | 2.2803890000 | -0.6795320000 | -0.34780700 |
| C | 2.6623230000 | -0.8395320000 | 0.99484400 |
| C | 3.1662210000 | -1.0940460000 | -1.35629500 |
| C | 3.8938980000 | -1.4167900000 | 1.30426900 |
| H | 1.9862680000 | -0.5220430000 | 1.77447700 |
| C | 4.3984840000 | -1.6637700000 | -1.04141800 |
| H | 2.8838190000 | -0.9738900000 | -2.40043600 |
| C | 4.7656580000 | -1.8293000000 | 0.29486500 |
| H | 4.1742450000 | -1.5424750000 | 2.34694400 |
| H | 5.0656500000 | -1.9824710000 | -1.83796100 |
| H | 5.7235080000 | -2.2768720000 | 0.54710900 |
| H | 2.0794520000 | 1.9910510000 | -1.18910600 |
| O | 1.3377990000 | 1.9007840000 | 0.75183800 |

| | | | |
|---|---|---|---|
| C | 1.8651180000 | 3.1615960000 | 1.13090400 |
| H | 1.1931900000 | 3.9799420000 | 0.83696400 |
| H | 1.9692540000 | 3.1426500000 | 2.21840600 |
| H | 2.8531740000 | 3.3363130000 | 0.67725200 |

TS09E

0 1

| | | | |
|---|---|---|---|
| C | -0.8155860000 | 0.0725610000 | 0.43980200 |
| C | 1.5342670000 | 0.3770460000 | 0.42853000 |
| N | 0.4397060000 | -0.3731270000 | -0.00982000 |
| C | -1.0279510000 | 1.5354140000 | -0.65076500 |
| C | 1.3584790000 | 1.8712390000 | -0.69427800 |
| C | 2.9307350000 | -0.1220290000 | 0.27147200 |
| O | 0.5086730000 | -0.9691340000 | -1.15744300 |
| C | 3.3780130000 | -0.8999160000 | -0.80988400 |
| C | 4.7131350000 | -1.2995630000 | -0.87476900 |
| C | 5.6212130000 | -0.9223690000 | 0.11555400 |
| C | 5.1864470000 | -0.1411520000 | 1.18802900 |
| C | 3.8529860000 | 0.2532470000 | 1.26390500 |
| H | -0.7350080000 | 0.5191160000 | 1.42798600 |
| H | 1.3573240000 | 0.7714580000 | 1.42561500 |
| H | 2.6712520000 | -1.2004650000 | -1.57109800 |
| H | 5.0443180000 | -1.9100030000 | -1.71060700 |
| H | 6.6601070000 | -1.2348550000 | 0.05349500 |
| H | 5.8821680000 | 0.1559690000 | 1.96792900 |
| H | 3.5195660000 | 0.8560940000 | 2.10599800 |

| | | | |
|---|---|---|---|
| C | 0.0796370000 | 2.3986970000 | -0.43278600 |
| H | -0.0583060000 | 3.2632980000 | 0.20913400 |
| H | 1.4704290000 | 1.2674190000 | -1.60099100 |
| H | 2.2181570000 | 2.5032030000 | -0.47949100 |
| C | -2.0170200000 | -0.8092690000 | 0.33344000 |
| C | -2.2562170000 | -1.6948270000 | -0.73069200 |
| C | -2.9696650000 | -0.7065550000 | 1.36159600 |
| C | -3.4149090000 | -2.4707690000 | -0.74168000 |
| H | -1.5254090000 | -1.7810950000 | -1.52289700 |
| C | -4.1293810000 | -1.4774420000 | 1.33805300 |
| H | -2.8023940000 | -0.0136390000 | 2.18239300 |
| C | -4.3539530000 | -2.3658690000 | 0.28549000 |
| H | -3.5852410000 | -3.1589090000 | -1.56524000 |
| H | -4.8539860000 | -1.3844440000 | 2.14210200 |
| H | -5.2569970000 | -2.9699620000 | 0.26449000 |
| H | -1.0930740000 | 0.9974330000 | -1.59837900 |
| N | -2.3489500000 | 2.1195710000 | -0.31172400 |
| O | -3.2615530000 | 1.9447750000 | -1.11123000 |
| O | -2.4433180000 | 2.7123090000 | 0.76545500 |

TS09Z

0 1

| | | | |
|---|---|---|---|
| C | -0.9292360000 | -0.0817630000 | 0.81705500 |
| C | 1.4462910000 | 0.0706410000 | 0.76810900 |
| N | 0.2737280000 | -0.3486780000 | 0.12383500 |
| C | -1.0920400000 | 1.7803900000 | 0.82061400 |

| | | | |
|---|---|---|---|
| C | 1.3532860000 | 1.9382000000 | 0.58302300 |
| C | 2.7791740000 | -0.3610100000 | 0.26339500 |
| O | 0.2708380000 | -0.6059510000 | -1.12746200 |
| C | 3.1263110000 | -0.4127250000 | -1.09774500 |
| C | 4.4167840000 | -0.7853630000 | -1.47406400 |
| C | 5.3779360000 | -1.1019080000 | -0.51278800 |
| C | 5.0440560000 | -1.0433570000 | 0.84159700 |
| C | 3.7568390000 | -0.6732230000 | 1.22366100 |
| H | -0.7725210000 | -0.2066050000 | 1.88550000 |
| H | 1.3584390000 | -0.0495500000 | 1.84400300 |
| H | 2.3810840000 | -0.1811420000 | -1.84591300 |
| H | 4.6702180000 | -0.8281210000 | -2.52987500 |
| H | 6.3804170000 | -1.3917220000 | -0.81594000 |
| H | 5.7827200000 | -1.2883470000 | 1.59990700 |
| H | 3.5030750000 | -0.6307370000 | 2.28085100 |
| C | 0.1603400000 | 2.3082730000 | 1.24199600 |
| H | -1.9488500000 | 1.9572420000 | 1.46409100 |
| H | 0.2128110000 | 2.5971570000 | 2.28980600 |
| H | 1.3564730000 | 1.9457710000 | -0.50575100 |
| H | 2.2868730000 | 2.2878640000 | 1.02153700 |
| C | -2.1962750000 | -0.7383330000 | 0.38018400 |
| C | -2.5744370000 | -0.9172770000 | -0.96129000 |
| C | -3.0681550000 | -1.1704470000 | 1.39443600 |
| C | -3.7892230000 | -1.5317660000 | -1.26475000 |
| H | -1.9147360000 | -0.5886910000 | -1.75178500 |

| | | | |
|---|---|---|---|
| C | -4.2814300000 | -1.7789610000 | 1.08455800 |
| H | -2.7884150000 | -1.0344470000 | 2.43727700 |
| C | -4.6443950000 | -1.9640220000 | -0.25087300 |
| H | -4.0682120000 | -1.6678390000 | -2.30589400 |
| H | -4.9383460000 | -2.1117680000 | 1.88346700 |
| H | -5.5892460000 | -2.4403260000 | -0.49839200 |
| N | -1.5705870000 | 2.0811550000 | -0.57238800 |
| O | -2.7818460000 | 2.2498590000 | -0.68934200 |
| O | -0.7575830000 | 2.1410690000 | -1.49016000 |

TS10

0 1

| | | | |
|---|---|---|---|
| C | -1.2273080000 | 0.2125620000 | 0.35717400 |
| C | 1.1323340000 | 0.1835310000 | 0.27878900 |
| N | -0.0908470000 | -0.5070820000 | -0.01260200 |
| C | -1.2149990000 | 1.5839100000 | -0.89203100 |
| C | 1.2129100000 | 1.3974950000 | -0.87577500 |
| C | 2.4126790000 | -0.6166110000 | 0.26316900 |
| O | -0.1246310000 | -1.1339940000 | -1.17748200 |
| C | 2.7051920000 | -1.5878210000 | -0.70652200 |
| C | 3.9269130000 | -2.2615720000 | -0.67354300 |
| C | 4.8771100000 | -1.9702640000 | 0.30672900 |
| C | 4.5957110000 | -1.0008800000 | 1.27045100 |
| C | 3.3712780000 | -0.3338750000 | 1.24770200 |
| H | -1.0734320000 | 0.7155180000 | 1.31157400 |
| H | 1.0338090000 | 0.6678860000 | 1.25335100 |

| | | | |
|---|---|---|---|
| H | 1.9546760000 | -1.8193970000 | -1.45145500 |
| H | 4.1364430000 | -3.0203270000 | -1.42324500 |
| H | 5.8281150000 | -2.4963320000 | 0.32210100 |
| H | 5.3231380000 | -0.7688280000 | 2.04399800 |
| H | 3.1545900000 | 0.4128980000 | 2.00949600 |
| C | 0.0300720000 | 2.2321250000 | -0.75145000 |
| H | 1.1759090000 | 0.7837840000 | -1.78498800 |
| H | 2.1739010000 | 1.9015850000 | -0.77899900 |
| C | -2.5854050000 | -0.4110340000 | 0.29500600 |
| C | -2.9426520000 | -1.4234730000 | -0.61015700 |
| C | -3.5563880000 | 0.0710350000 | 1.18895400 |
| C | -4.2378630000 | -1.9428970000 | -0.59960000 |
| H | -2.1876330000 | -1.7993660000 | -1.28845300 |
| C | -4.8516390000 | -0.4418290000 | 1.18590100 |
| H | -3.2915260000 | 0.8536860000 | 1.89731800 |
| C | -5.1970180000 | -1.4550220000 | 0.28929300 |
| H | -4.4980780000 | -2.7352860000 | -1.29680700 |
| H | -5.5867970000 | -0.0563730000 | 1.88752300 |
| H | -6.2051210000 | -1.8613530000 | 0.28582500 |
| H | -1.2710290000 | 0.8872720000 | -1.73501200 |
| H | -2.1035580000 | 2.1918370000 | -0.73316800 |
| O | 0.0189180000 | 3.4613350000 | -0.17185500 |
| C | 1.2544070000 | 4.1214760000 | 0.07839100 |
| H | 0.9901750000 | 5.1105670000 | 0.45612300 |
| H | 1.8517060000 | 3.5945480000 | 0.83312300 |

| | | | |
|---|---|---|---|
| H | 1.8443480000 | 4.2273980000 | -0.84027300 |

TS11E

0 1

| | | | |
|---|---|---|---|
| C | 0.1527310000 | -0.4633750000 | -0.53340200 |
| C | -2.1238230000 | 0.1367940000 | -0.51768200 |
| N | -1.1340610000 | -0.7006990000 | 0.01536300 |
| C | 0.7065190000 | 0.9835420000 | 0.05635200 |
| C | -1.6228410000 | 1.7137030000 | 0.16779200 |
| O | -1.1940910000 | -1.0087940000 | 1.27072600 |
| H | 0.1495200000 | -0.4458300000 | -1.61970000 |
| H | -2.0327820000 | 0.2172680000 | -1.60075900 |
| C | -0.2950500000 | 1.9909770000 | -0.23492100 |
| H | -0.0633640000 | 2.7363260000 | -0.99052400 |
| H | -1.7295740000 | 1.3611570000 | 1.20652200 |
| H | -2.3779060000 | 2.4491980000 | -0.11179600 |
| H | 0.7777250000 | 0.7124700000 | 1.12407200 |
| O | 1.9534180000 | 1.1840470000 | -0.55325500 |
| C | 2.9897850000 | 1.5647920000 | 0.34658800 |
| H | 3.9000020000 | 1.6519090000 | -0.25088400 |
| H | 2.7721510000 | 2.5335730000 | 0.81738400 |
| H | 3.1354980000 | 0.8053270000 | 1.12494300 |
| C | -3.5321620000 | -0.1461840000 | -0.03864700 |
| H | -4.2154470000 | 0.6253320000 | -0.40505800 |
| H | -3.8693040000 | -1.1159970000 | -0.42040900 |
| H | -3.5638330000 | -0.1785050000 | 1.05152900 |

| | | | |
|---|---|---|---|
| N | 1.1213260000 | -1.5617120000 | -0.14170000 |
| O | 1.7139660000 | -1.4761770000 | 0.92286700 |
| O | 1.2211470000 | -2.4736830000 | -0.95501800 |

TS11Z

0 1

| | | | |
|---|---|---|---|
| C | 0.3617930000 | -0.6166620000 | -0.68259900 |
| C | -1.9826890000 | -0.3994440000 | -0.47206100 |
| N | -0.7894590000 | -0.9148840000 | 0.09529400 |
| C | 0.6225380000 | 1.0945520000 | -0.60711900 |
| C | -1.7863630000 | 1.3331900000 | -0.35696700 |
| O | -0.7401700000 | -1.1240300000 | 1.34688500 |
| H | 0.2143490000 | -0.8568860000 | -1.73164100 |
| H | -2.0263560000 | -0.6183920000 | -1.53956700 |
| C | -0.5992840000 | 1.7035990000 | -1.06488000 |
| H | -0.6669120000 | 1.9617340000 | -2.11963200 |
| H | -1.7215640000 | 1.4144620000 | 0.73429600 |
| H | -2.7203090000 | 1.7515350000 | -0.73754900 |
| H | 1.5004940000 | 1.2594120000 | -1.24277600 |
| O | 0.9412320000 | 1.2154900000 | 0.76077600 |
| C | 1.5091330000 | 2.4767540000 | 1.08167000 |
| H | 0.8158190000 | 3.2964770000 | 0.84934600 |
| H | 1.7153880000 | 2.4584390000 | 2.15391800 |
| H | 2.4496150000 | 2.6417460000 | 0.53444200 |
| N | 1.6452650000 | -1.2927140000 | -0.28243600 |
| O | 1.6698790000 | -2.0884340000 | 0.63633700 |

| | | | |
|---|---|---|---|
| O | 2.5975650000 | -0.9824040000 | -1.00063800 |
| C | -3.2347830000 | -0.8057950000 | 0.27978200 |
| H | -3.2065910000 | -0.4389360000 | 1.30727000 |
| H | -4.1153340000 | -0.4017090000 | -0.22805000 |
| H | -3.3193610000 | -1.8968270000 | 0.31610300 |

TS12

0 1

| | | | |
|---|---|---|---|
| C | -1.9366760000 | 0.1692960000 | -0.93006900 |
| C | 0.0340390000 | 0.3313620000 | 0.20718500 |
| N | -0.5719850000 | -0.1273290000 | -1.01550900 |
| C | -2.4911860000 | -1.1457570000 | 0.15066100 |
| C | -0.3636960000 | -0.9057540000 | 1.29965900 |
| C | -1.8053940000 | -0.9699590000 | 1.38078600 |
| O | -0.1486980000 | -1.1689210000 | -1.62771600 |
| H | -3.5819770000 | -1.1156130000 | 0.18197900 |
| H | -2.1014620000 | -1.9487150000 | -0.49442500 |
| H | 0.0907380000 | -1.7541410000 | 0.77078800 |
| H | 0.1488400000 | -0.7083440000 | 2.24433600 |
| H | -2.3237570000 | -0.5812620000 | 2.25347300 |
| C | -2.0463230000 | 1.5238760000 | -0.25083900 |
| C | -0.7026130000 | 1.6382870000 | 0.52828400 |
| H | -2.1481790000 | 2.3267640000 | -0.98835200 |
| H | -0.8576750000 | 1.7410510000 | 1.60561900 |
| H | -0.1079630000 | 2.4885270000 | 0.18915500 |
| H | -2.9140360000 | 1.5675020000 | 0.41322800 |

| | | | |
|---|---|---|---|
| C | 1.5512730000 | 0.4859600000 | 0.10251500 |
| O | 2.1033820000 | 1.5602160000 | -0.00991500 |
| O | 2.1947760000 | -0.6859340000 | 0.15121600 |
| C | 3.6221600000 | -0.6146400000 | -0.01396300 |
| H | 4.0687920000 | -0.0057590000 | 0.77644000 |
| H | 3.9714960000 | -1.6451710000 | 0.04560500 |
| H | 3.8682990000 | -0.1792720000 | -0.98543100 |
| H | -2.4743920000 | -0.0131680000 | -1.85783400 |

TS13

0 1

| | | | |
|---|---|---|---|
| C | 1.2460120000 | 0.0353570000 | 0.12040100 |
| C | -1.0086200000 | 0.4069260000 | 0.29285300 |
| N | -0.0265090000 | -0.5553600000 | -0.07457100 |
| C | 1.3526650000 | 1.0776240000 | -1.28452600 |
| C | -1.0398830000 | 1.4463070000 | -1.11801700 |
| C | 0.2710160000 | 2.0098140000 | -1.22402700 |
| O | -0.2135210000 | -1.4185600000 | -1.00498700 |
| H | 2.3608500000 | 1.4919810000 | -1.29128300 |
| H | 1.2193620000 | 0.2811610000 | -2.03292800 |
| H | -1.2728000000 | 0.6661290000 | -1.85425700 |
| H | -1.8626570000 | 2.1544480000 | -0.99798900 |
| H | 0.4497490000 | 3.0429950000 | -0.93718400 |
| C | 2.3953710000 | -0.9539100000 | 0.03572000 |
| C | 1.1137120000 | 0.8672710000 | 1.39292700 |
| C | -0.4136020000 | 1.1371490000 | 1.49964200 |

| | | | |
|---|---|---|---|
| C | 4.7411430000 | -1.0712250000 | 0.14193200 |
| O | 3.6027550000 | -0.2353970000 | 0.19722500 |
| H | 2.2802790000 | -1.7056090000 | 0.83571600 |
| H | 2.3626810000 | -1.4841200000 | -0.92599900 |
| H | 1.4804420000 | 0.3040200000 | 2.25794100 |
| H | -0.6496810000 | 2.2038330000 | 1.46105900 |
| H | 4.7278410000 | -1.8277270000 | 0.94251400 |
| H | 5.6164870000 | -0.4299560000 | 0.27205600 |
| H | 4.8160800000 | -1.5915440000 | -0.82573200 |
| H | -0.8380280000 | 0.7354410000 | 2.42199700 |
| H | 1.7002950000 | 1.7860970000 | 1.32689800 |
| C | -2.4081840000 | -0.1493700000 | 0.51840800 |
| O | -2.9558270000 | -0.1442360000 | 1.60168600 |
| O | -2.9682460000 | -0.6194250000 | -0.60245500 |
| C | -4.2745340000 | -1.1999160000 | -0.44370500 |
| H | -4.9812630000 | -0.4554920000 | -0.06736900 |
| H | -4.5619810000 | -1.5392580000 | -1.43862700 |
| H | -4.2339780000 | -2.0400890000 | 0.25379200 |
